# The Impact of Dental Implant Length on Failure Rates: A Systematic Review and Meta-Analysis

**DOI:** 10.3390/ma14143972

**Published:** 2021-07-16

**Authors:** Maha Abdel-Halim, Dalia Issa, Bruno Ramos Chrcanovic

**Affiliations:** 1 Faculty of Odontology, Malmö University, 214 21 Malmö, Sweden; abdelhalimmaha@gmail.com (M.A.-H.); dalia.issa97@hotmail.com (D.I.); 2 Department of Prosthodontics, Faculty of Odontology, Malmö University, 214 21 Malmö, Sweden

**Keywords:** dental implant, failure, implant length, systematic review, meta-analysis

## Abstract

The present review aimed to evaluate the impact of implant length on failure rates between short (<10 mm) and long (≥10 mm) dental implants. An electronic search was undertaken in three databases, as well as a manual search of journals. Implant failure was the outcome evaluated. Meta-analysis was performed in addition to a meta-regression in order to verify how the risk ratio (RR) was associated with the follow-up time. The review included 353 publications. Altogether, there were 25,490 short and 159,435 long implants. Pairwise meta-analysis showed that short implants had a higher failure risk than long implants (RR 2.437, *p* < 0.001). There was a decrease in the probability of implant failure with longer implants when implants of different length groups were compared. A sensitivity analysis, which plotted together only studies with follow-up times of 7 years or less, resulted in an estimated increase of 0.6 in RR for every additional month of follow-up. In conclusion, short implants showed a 2.5 times higher risk of failure than long implants. Implant failure is multifactorial, and the implant length is only one of the many factors contributing to the loss of an implant. A good treatment plan and the patient’s general health should be taken into account when planning for an implant treatment.

## 1. Introduction

The placement of a dental implant of a certain length is not always possible due to anatomical limitations. These limitations usually exist due to two main factors. First, horizontal and vertical reduction of the alveolar bone is expected after tooth extractions [1]. This alveolar bone resorption will lead to insufficient bone height for the placement of implants of regular and long lengths. Second, anatomical structures, including inferior alveolar canal, mental foramina, and the presence of a low located maxillary sinus, might also hinder the use of longer implants [2].

To overcome these limitations there are a few options, such as sinus elevations, vertical bone augmentation, alveolar nerve transposition, and zygomatic implants [2,3]. These options allow the placement of longer implants which in turn enables a longer bone-to-implant contact area. However, these are all invasive surgical options associated with an increased risk of complications. Sinus perforation, mandibular paresthesia, graft failure, increased risk of infection, and donor site morbidity have been reported for these procedures [3,4,5,6]. Shorter dental implants can predictably be placed to minimize or even avoid the risks that come with the mentioned invasive surgical treatments. Reduced interventions, costs, and surgical time are other advantages that come with the placement of shorter implants compared to the other mentioned options for placing longer implants [2,7].

Some previous reviews [8,9] on the subject observed that short implants do not present a statistically significant difference in survival rate in comparison to long implants. Other reviews [10,11] have pointed out, however, that short implants may present a greater risk for failure compared to longer implants. To better assess this issue, the purpose of the present systematic review was to evaluate the impact of implant length on failure rates between short (<10 mm) and long (≥10 mm) dental implants, without the restriction of the inclusion criterion to randomized clinical trials.

## 2. Materials and Methods

This study followed the PRISMA Statement guidelines. Registration in PROSPERO was undertaken with the registration number CRD42021238272.

### 2.1. Objective

The purpose of this systematic review was to evaluate the impact of implant length on failure rates between short (<10 mm) and long (≥10 mm) dental implants.

The focused question was elaborated by using the PICO format (participants, interventions, comparisons, and outcomes): In patients undergoing implant-prosthetic rehabilitation (participants), how do short implants (intervention) compared with long implants (comparison) affect the implant failure rate (outcome)?

### 2.2. Search Strategies

An electronic search without time or language restrictions was undertaken in January 2020 in the following databases: PubMed/Medline, Web of Science, and Science Direct. A last complementary search was performed in December 2020. The following terms were used in the search strategy:

(dental implant OR oral implant) AND (“implant length”)

A manual search of dental-implants-related journals was performed, including the following: Clinical Implant Dentistry and Related Research, Clinical Oral Implants Research, European Journal of Oral Implantology, Implant Dentistry, International Journal of Implant Dentistry, International Journal of Oral and Maxillofacial Implants, International Journal of Oral Implantology, International Journal of Prosthodontics, Journal of Clinical Periodontology, Journal of Oral Implantology, Journal of Periodontology, Journal of Prosthetic Dentistry, Journal of Prosthodontics, and Journal of Prosthodontic Research. The reference list of the identified studies and the relevant reviews on the subject was also checked for possible additional studies.

### 2.3. Inclusion and Exclusion Criteria

Eligibility criteria included clinical human studies, either randomized or not, comparing implant failure rates in any group of patients receiving implants rehabilitated with long and short dental implants. No follow-up time restrictions were imposed, since most dental implant failures usually happen in the early postoperative period, regardless of a long follow-up [12,13].

Exclusion criteria included case reports, technical reports, biomechanical studies, finite element analysis (FEA) studies, animal studies, in vitro studies, reviews papers, and grey literature.

Other reasons for the exclusion of a publication were: (a) when there were implants in only one of the groups defined for the present review, either short (<10 mm) or long (≥10 mm) implants; (b) when the number of implant failures and/or the total number of implants per group of different lengths was not reported; (c) when implant failure was analyzed in an effect size (for example, in odds ratio); (d) when the report of implant failures included one group with implant lengths that encompassed both groups of the present review (example: a study that reported failures of a group that consisted of implants with lengths between 9 and 11.5 mm, which would make distinguishing between the precise number of <10 mm and ≥10 mm implants impossible); (e) when there was no reply from the authors of the publications that were contacted by e-mail in order to ask for additional information (the aforementioned cases ‘b’, ‘c’, and ‘d’); and (f) when a longer follow-up article of the same study was available, the shorter follow-up article was excluded.

For this review, implant failure represents the complete loss of the implant.

### 2.4. Study Selection

The titles and abstracts of all reports identified through electronic searches were read independently by the three authors. For studies appearing to meet the inclusion criteria, or for which there were insufficient data in the title and abstract to make a clear decision, the full report was obtained. Disagreements were resolved by discussion between the authors.

### 2.5. Quality Assessment

Quality assessment of the studies was executed according to the Quality Assessment Tool of the National Institutes of Health (NIH) [14]. Studies of “good” quality were judged to have at least 7 points.

### 2.6. Data Extraction

From the studies included in the final analysis, the following data were extracted: year of publication, study design, country, study setting (University, private practice, etc.), number of patients and sex distribution, patients’ age, prosthetic loading protocol, implant location (maxilla/mandible), number of failed and placed implants for each length category, implant system, presence of smokers in the patients’ study group, and follow-up time. Contact with authors for possible missing data was performed.

### 2.7. Analyses

A meta-analysis was performed having implant failure as the dichotomous outcome measure evaluated. The statistical unit for ‘implant failure’ was the implant. Whenever outcomes of interest were not clearly stated, the data were not used for analysis. The I^2^ statistic was used to express the percentage of total variation across the studies due to heterogeneity. The inverse variance method was used for random-effects or fixed-effects models. Where statistically significant (*p* < 0.10) heterogeneity was detected, a random-effects model was used to assess the significance of treatment effects. The estimate of the relative effect for dichotomous outcomes was expressed in risk ratio (RR), with a 95% confidence interval (CI). Only if there were studies with similar comparisons reporting the same outcome measures was meta-analysis attempted.

The untransformed proportion with random-effects DerSimonian–Laird method [15] of implant failure was calculated with consideration of specific groups of implant lengths (subgroup analyses). In order to explore the possible heterogeneity of effect between studies, a meta-regression was performed in order to verify how the RR was associated with the time of follow-up. The data were analyzed using the statistical software OpenMeta [Analyst] [16].

A funnel plot was drawn, and it was generated with the software OpenMEE [17].

## 3. Results

### 3.1. Literature Search

**Figure 1:**
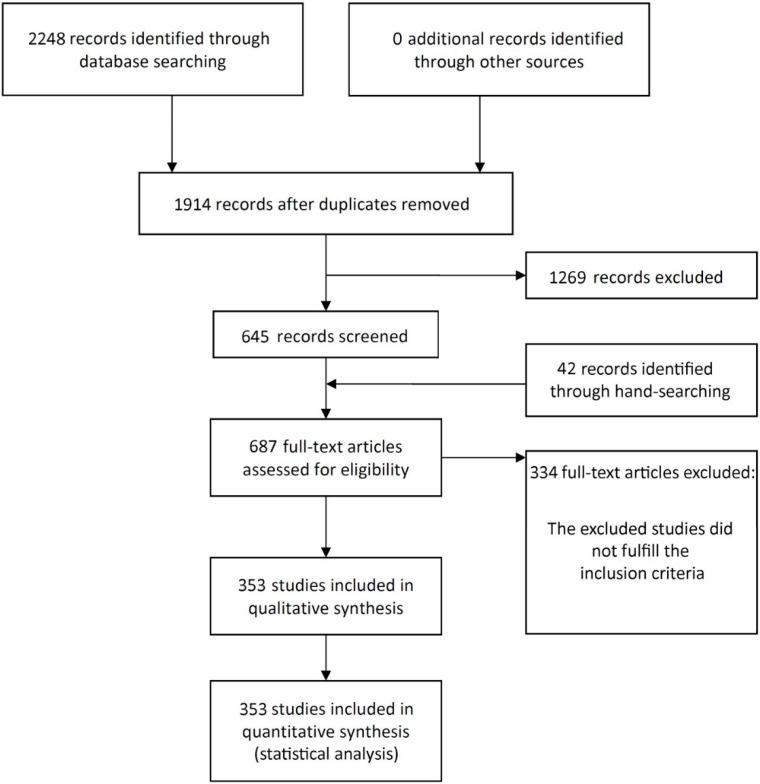
Study screening process.

The study selection process is summarized in Figure 1. The search strategy in the databases resulted in 2248 papers (516 in PubMed, 519 in Web of Science, 1213 in ScienceDirect). In the end, 353 publications were included in the review (see Supplementary Material for a list of included articles).

### 3.2. Description of the Studies

Table S1 presents detailed data of the 353 included studies. The articles were published between 1990 and 2020. A total of 245 studies were unicenter, 88 were multicenter, and 20 studies could not be categorized due to unclear information. In terms of study design, 60 studies were randomized clinical trials (RCT), 94 were prospective studies (without a pre-established control group), 6 were prospective controlled clinical trials, and 186 were retrospective observational studies. For 155 studies, at least one university was reported as the institution where the study was carried out; private dental practices accounted for 145 studies, hospitals for 29 studies, non-profit organizations for 5 studies, and veteran medical centers for 2 studies. Multicenter studies could include two or more different types of institutions. For 32 studies, it was not possible to get information on the type of institution where the study was performed. A total of 45 studies were carried out in Sweden (other countries could be included in multicenter studies). Other common places for the studies were Italy in 85 cases, USA in 45, Belgium in 20, Spain in 19, Switzerland in 17, Brazil and Germany in 14 cases each, Canada in 8, and Turkey in 6 cases, among others (the same observation for multicenter studies is applied here).

The mean follow-up ± standard deviation of 310 studies was 41.75 ± 35.49 months (min–max, 3–300). For the other 43 studies, there was neither information on the precise time of follow-up (for example, 12 months, or 60 months) nor the mean follow-up time. Information on follow-up in these 43 studies was usually reported, for example, in the following ways: “patients were followed up between the years 2002 to 2004”, or “patients were followed up for up to 84 months”.

Immediate prosthetic loading of the implants was applied in 91 studies, early loading in 27 studies, and delayed loading in 262 studies. These loading protocols (immediate, early, or delayed) could be either separately applied for all implants of a study, or a combination of them could be applied for different implants of the same study. For 4 studies, the implants were not loaded, and for 16 studies, this information was not available.

Most of the studies (n = 209) included implants installed in the maxilla and mandible; 92 studies included patients that received implants only in maxillae, and the other 52 studies included only implants placed in mandibles.

Smokers were excluded from 25 studies. Information on the presence or the absence of smokers among the patients was not available for 96 studies.

Altogether, there were 25,490 short implants (<10 mm) and 159,435 long implants (≥10 mm), and 941 and 4185 implant failures in these groups, respectively. Implants most commonly used were from the following manufacturers: Nobel Biocare (Göteborg, Sweden) in 126 studies, Straumann (Basel, Switzerland) in 87 studies, and Astra Tech (Mölndal, Sweden) in 37 studies. Information on which implant brand and/or system used was not available in 12 studies.

### 3.3. Quality Assessment

Almost all included studies (352 out of 353) were classified as “good” according to the quality assessment tool (Table S2). Only one study was classified as presenting a “fair” quality. However, it was deemed not sufficient to invalidate its results due to fact that the patients were not recruited consecutively, and the statistical methods were not well-described, although the outcome information necessary for the present review (implant failure) was clearly available.

### 3.4. Meta-Analyses

A random-effects model was used to evaluate the implant failure in the comparison between the implant length groups, since statistical heterogeneity was observed (τ^2^ = 0.284, Chi^2^ = 558.932, I^2^ = 37.023, *p* < 0.001).

The pairwise meta-analysis showed that short implants (<10 mm) had a higher risk of failure than long implants (≥10 mm), with an RR of 2.437 (95% CI 2.179, 2.725, *p* < 0.001; Figure S1). An RR of 2.437 implies that failures of short implants present a 2.437 higher risk of occurring than failures of long implants; i.e., short implants have a higher risk of failure by 143.7% in relation to long implants.

Table 1 lists the probability of implant failure for each implant length group. There was a decrease in the probability of implant failure with longer implants.

### 3.5. Meta-Regression

A total of 310 studies provided clear information about the follow-up time or mean follow-up time. For most of the other 43 studies, either one of the two forms of information was reported, from which no precise follow-up time could be obtained: (a) a survival analysis, either lifetable or Kaplan–Meier analysis, with no mean follow-up time provided; or (b) a follow-up time between a certain number of years with no mean follow-up time provided.

When a meta-regression that considered the follow-up period as a covariate in relation to RR was plotted for these 310 studies, it was observed that the follow-up time did not have any effect on the RR of implant failure between short and long implants. The first-degree equation resulted from the linear regression of this meta-regression was

y = 0.882 − 0.000x, where:

Intercept = 0.882 (0.690, 1.074), standard error 0.098, *p* < 0.001

Follow-up = 0.000 (−0.003, 0.003), standard error 0.002, *p* = 0.905

A sensitivity analysis of the meta-regression was performed plotting together only the studies (n = 285) with follow-up up until 7 years. The first-degree equation that resulted from the linear regression of this sensitivity analysis was

y = 0.667 + 0.006x, where:

Intercept = 0.667 (0.409, 0.924), standard error 0.131, *p* < 0.001

Follow-up = 0.006 (0.000, 0.013), standard error 0.003, *p* = 0.036

**Figure 2:**
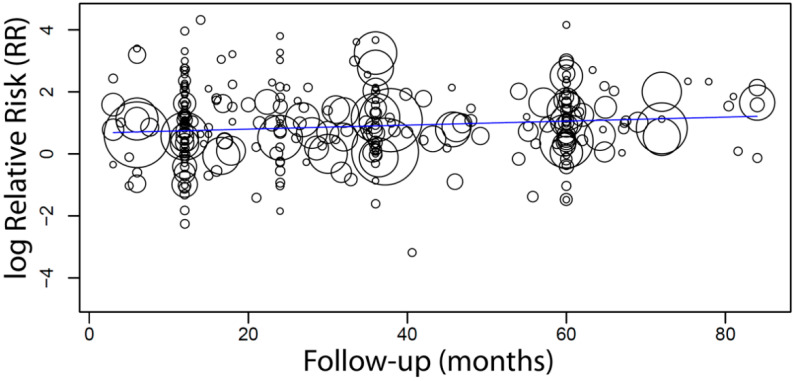
Scatter plot for the sensitivity analysis of the meta-regression, including only studies with a follow-up time of up to 7 years (n = 285), with the association between the relative risk (RR) of implant failure between short and long implants and the follow-up time (in months). Every circle represents a study, and the size of the circle represents the weight of the study in the analysis.

In this case, there was an estimated increase of 0.6 in RR for every additional month of follow-up (Figure 2).

### 3.6. Publication Bias

**Figure 3:**
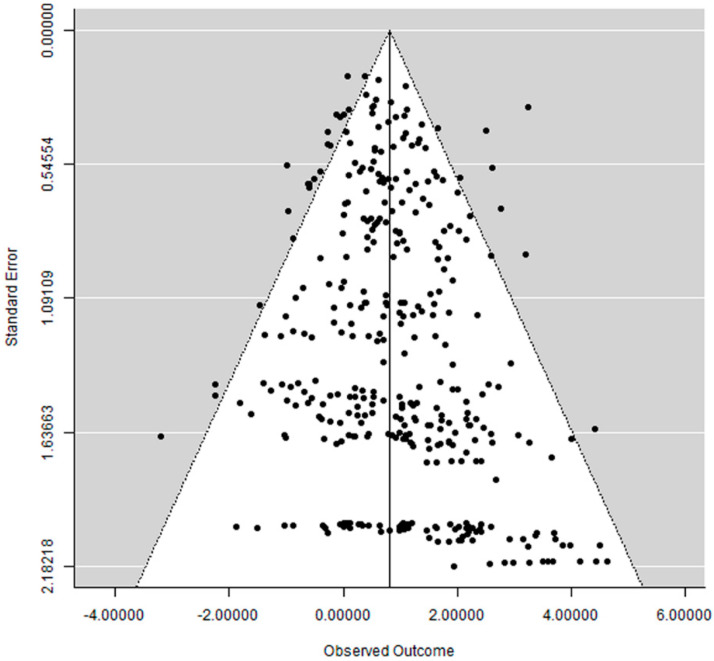
Funnel plot.

The funnel plot did not show a clear asymmetry (Figure 3), which indicates a possible absence of publication bias.

## 4. Discussion

To the best of our knowledge, this is the first meta-analysis comparing the clinical outcomes of short (<10 mm) and longer implants (≥10 mm) from all articles with available data. The results of the present study suggest that short dental implants have a higher risk of failure than longer implants. Previous meta-analyses on the subject reported no statistically significant difference in failure rates between short (≤8 or <10 mm) and long (≥10 mm) implants [8], and between extra-short (≤6 mm) and longer (≥10 mm) implants [9]; these previous results differ from the present review. The peculiarity of the present study lies in it including all data available and not limiting the inclusion criteria, as was done, for example, in a previous review that included only rough-surface dental implants [8], or in another which only included RCTs [9]. Two other reviews [10,11], evaluating only implants placed in the posterior area of the jaws, suggested that extra-short implants, namely ≤6 mm [11] and <8 mm [10], may present a greater risk of failure compared to implants longer than these lengths.

Some hypotheses have been proposed to try to explain the reasons behind a higher implant failure rate among short implants in comparison to longer implants.

One hypothesis relates to the implant location in the jaws. The posterior region of the jaws is usually limited in bone height available for dental implants due to the pneumatization of the maxillary sinus and due to the presence of the inferior alveolar neurovascular bundle. The anterior region of the jaws may also be affected in cases of alveolar ridge deficiencies due to advanced bone resorption when a long time after tooth extractions has passed, as the bony socket undergoes significant horizontal and vertical reduction following extraction [18]. When patients in need of implants are unwilling or cannot be submitted to surgical procedures to increase the available bone height, such as distraction osteogenesis [19], bone grafting [20], and alveolar nerve transposition [6], the planned implant site can present reduced bone left for the placement of dental implants. This fact, together with the usual low-density bone (especially for the posterior maxilla [21]) and occlusal overload (especially for the posterior region of the jaw, due to the greater bite force in the posterior dental arch [22]), can predispose the short implant to a higher risk of failure. This may mean that short implants placed in the posterior regions in sites with poor bone quality and submitted to higher occlusal forces are more likely to fail. This could result in excessive bone resorption and loss of osseointegration in the long term [23,24], yet the placement of short implants in jaw locations with less available bone is a less morbid alternative than to submit the patient to further and more complex surgical procedures, such as the aforementioned distraction osteogenesis, bone grafting, and alveolar nerve transposition.

The dimensional relationship between the implant-supported prosthetic restoration height and the implant length, the so-called crown-to-implant ratio (CIR), is another possible reason. Short implants are more likely to have an inappropriate CIR [25]. Some studies suggest that a deviation in the CIR may result in excessive occlusal and non-axial loading [26,27]; this can enhance marginal bone loss [28,29] and mechanical problems [30]. A high deviation factor between crown and implant length is one of the reasons suggested that may lead to short implant longevity [31].

Another hypothesis states that short implants would be more likely to fail if peri-implantitis occurs due to the lower quantity of bone support [8]. If implants with different lengths suffer the same quantity of marginal bone loss over time, it is logical to assume that the shorter implant will lose a higher proportion of supporting bone in relations to its total length than a longer implant over the same period of time.

Related to the previous hypothesis, it can be said that compared with longer implants with a comparable diameter, there is less bone to implant contact when short implants are used, simply because there is less implant surface [31].

Many of the studies in the present review include smokers. The registered number of cigarettes of each patient per day has to be interpreted with caution since patients usually under-register how many cigarettes they smoke per day. It is therefore hard to study smoking as a single possible reason affecting short implant failure. However, we know for sure that smoking is one of the main factors that have been shown to have an effect on implant failure [32], and it has a dose-related effect on osseointegration [33].

Patients that have lost their teeth due to periodontitis usually have lower bone height because of the bone resorption process that takes place compared to patients that lost their teeth due to trauma or caries. This means that patients treated for periodontitis are usually treated with shorter implants. Studies have shown that patients with a history of periodontitis have a higher potential risk of experiencing complications around implants, including higher bone loss and peri-implantitis. This, together with shorter implants in lower bone height, means a higher risk of implant failure [34].

A combination of two or more of the previous factors may magnify the risk of failure among short implants. 

### Limitations of the Present Study

The results of the present study have to be interpreted with caution because of its limitations. First of all, all confounding factors may have affected the long-term outcomes and not just the fact that implants were short or long. The included studies have a considerable number of confounding factors, and most of the studies, if not all, did not state how many implants were inserted, survived, or were lost in several different conditions. Due to the inclusion of a massive number of studies, it was decided to collect data only on a couple of confounding factors, namely the patients’ age and sex, the presence of smokers, the prosthetic loading protocol, the placement of implants in one or both jaws, and the implant system(s) used. Other factors such as the use of grafting, irradiation of the head and neck region [35], the insertion of implants in fresh extraction sockets [36], different prosthetic configurations, implants of different diameters, type of opposing dentition, different implant angulation ranges, splinting of the implants, the intake of medicaments by the patients [37,38], or the presence of patients with bruxism [39,40,41] diabetes [42] were not always reported. Moreover, several professionals were involved in the treatment of these patients, and there was a considerable variability of surgical and prosthetic approaches applied by these different professionals; therefore, the influence of different surgeons on the implant failure rate must be taken into account [43]. The impact of these variables on the implant survival rate is difficult to estimate if these factors are not identified separately between the different implant groups in order to perform a meta-regression analysis. It is a fact that individual patients sometimes present with more than one risk factor [44], and groups of patients are typically heterogeneous with respect to risk factors and susceptibilities, so the specific effect of an individual risk factor could be isolated neither for individual studies nor for the present review. This is understandable and expected because study populations are typically representative of normal populations with various risk factors [45]. The lack of control of the confounding factors, therefore, limited the potential for drawing robust conclusions.

Second, most of the included studies had a retrospective design, and the nature of a retrospective study inherently results in flaws, which manifest in gaps in information and incomplete records.

Third, much of the research in the field is limited by small cohort size and short follow-up periods. A longer follow-up period can lead to an increase in the failure rate, especially if it extended beyond functional loading, because other prosthetic factors can influence implant failure from that point onward. This might have led to an underestimation of actual failures in some studies.

Fourth, some included studies are characterized by a low level of specificity, where the assessment of the implant length as a complicating factor for dental implants was not the main focus of the investigation.

## 5. Conclusions

As a conclusion, the present study suggests that short implants (<10 mm) had approximately a 2.5 times higher risk of failure than long implants (≥10 mm). Since implant failure is multifactorial and the implant length is only one of many factors contributing to the loss of the implant, short dental implants are still an alternative for implant placement; however, a good treatment plan and the patient’s general health should be taken into account when planning for an implant treatment.

## Figures and Tables

**Table 1:** Probability of implant failure for each implant length group.

Length Group (mm)	Studies (n)	Failure/Total of Implants	Probability of Failure * (95% CI), SE, *p* Value	Heterogeneity
4–6.5	72	108/2549	4.6% (3.3, 6.0), 0.7%, *p* < 0.001	τ^2^ = 0001, Chi^2^ = 157.702, I^2^ = 54.98%, *p* < 0.001
7–9.5	307	573/11,940	3.8% (3.3, 4.3), 0.3%, *p* < 0.001	τ^2^ = 0.000, Chi^2^ = 646.944, I^2^ = 52.70%, *p* < 0.001
10–12	316	1588/47,196	2.7% (2.4, 2.9), 0.1%, *p* < 0.001	τ^2^ = 0.000, Chi^2^ = 1273.409, I^2^ = 75.26%, *p* < 0.001
13–15	225	1312/40,033	2.5% (2.2, 2.8), 0.2%, *p* < 0.001	τ^2^ = 0.000, Chi^2^ = 970.310, I^2^ = 76.92%, *p* < 0.001
16–20	119	212/8486	1.8% (1.4, 2.1), 0.2%, *p* < 0.001	τ^2^ = 0.000, Chi^2^ = 136.446, I^2^ = 13.52%, *p* = 0.118

95% CI—95% confidence interval, SE—standard error. * Untransformed proportion, random-effects DerSimonian–Laird method.

## Data Availability

The data presented in this study are available within the article and supplementary material.

## Supplementary Materials

The following are available online at https://www.mdpi.com/article/10.3390/ma14143972/s1, Reference list of the included articles, Table S1: Detailed data of the included studies, Table S2: Quality assessment tool, according to the National Institutes of Health (NIH), Figure S1: Forest plot for the event ‘implant failure’.
