# Supplementary material for: The Impact of Dental Implant Length on Failure Rates: A Systematic Review and Meta-Analysis
Source: Materials (Basel). 2021 Jul 16;14(14):3972. doi: 10.3390/ma14143972 (PMC8307721; doi:10.3390/ma14143972)

SUPPLEMENTARY MATERIAL

The impact of dental implant length on failure rates: a systematic review and meta-analysis

Reference list of the included studies

1. Adánez MH, Brezavšček M, Vach K, Fonseca M, Att W. Clinical and Radiographic Evaluation of Short Implants Placed in the Posterior Mandible: A 1-Year Pilot Split-Mouth Study. J Oral Implantol. 2018 Aug;44(4):250-259.
2. Alayan J, Ivanovski S. Biological and technical outcomes of restored implants after maxillary sinus augmentation-Results at 1-year loading. Clin Oral Implants Res. 2019 Sep;30(9):849-860.
3. Al-Nawas B, Domagala P, Fragola G, Freiberger P, Ortiz-Vigón A, Rousseau P, Tondela J. A Prospective Noninterventional Study to Evaluate Survival and Success of Reduced Diameter Implants Made From Titanium-Zirconium Alloy. J Oral Implantol. 2015 Aug;41(4):e118-25.
4. Alsaadi G, Quirynen M, Komárek A, van Steenberghe D. Impact of local and systemic factors on the incidence of oral implant failures, up to abutment connection. J Clin Periodontol. 2007 Jul;34(7):610-7.
5. Alsaadi G, Quirynen M, Komárek A, van Steenberghe D. Impact of local and systemic factors on the incidence of late oral implant loss. Clin Oral Implants Res. 2008 Jul;19(7):670-6.
6. Amato F, Polara G, Spedicato GA. Immediate Loading of Fixed Partial Dental Prostheses on Extra-Short and Short Implants in Patients with Severe Atrophy of the Posterior Maxilla or Mandible: An Up-to-4-year Clinical Study. Int J Oral Maxillofac Implants. 2020 May/Jun;35(3):607-615.
7. Amorfini L, Migliorati M, Drago S, Silvestrini-Biavati A. Immediately Loaded Implants in Rehabilitation of the Maxilla: A Two-Year Randomized Clinical Trial of Guided Surgery versus Standard Procedure. Clin Implant Dent Relat Res. 2017 Apr;19(2):280-295.
8. Andersson P, Degasperi W, Verrocchi D, Sennerby L. A Retrospective Study on Immediate Placement of Neoss Implants with Early Loading of Full-Arch Bridges. Clin Implant Dent Relat Res. 2015 Aug;17(4):646-57.
9. Anitua E, Flores C, Flores J, Alkhraisat MH. Clinical Effectiveness of 6.5-mm-Long Implants to Support Two-Implant Fixed Prostheses in Premolar-Molar Region: The Influence of Immediate Loading and the Length of Splinting Implant. J Prosthodont. 2019 Feb;28(2):e688-e693.
10. Anitua E, Flores J, Flores C, Alkhraisat MH. Long-term Outcomes of Immediate Loading of Short Implants: A Controlled Retrospective Cohort Study. Int J Oral Maxillofac Implants. 2016 Nov/Dec;31(6):1360-1366.
11. Anitua E, Orive G, Aguirre JJ, Ardanza B, Andía I. 5-year clinical experience with BTI dental implants: risk factors for implant failure. J Clin Periodontol. 2008 Aug;35(8):724-32.
12. Aparicio C, Orozco P. Use of 5-mm-diameter implants: Periotest values related to a clinical and radiographic evaluation. Clin Oral Implants Res. 1998 Dec;9(6):398-406.
13. Arlin ML. Short dental implants as a treatment option: results from an observational study in a single private practice. Int J Oral Maxillofac Implants. 2006 Sep-Oct;21(5):769-76.
14. Arora H, Ivanovski S. Immediate and early implant placement in single-tooth gaps in the anterior maxilla: A prospective study on ridge dimensional, clinical, and aesthetic changes. Clin Oral Implants Res. 2018 Nov;29(11):1143-1154.
15. Arosio P, Greco GB, Zaniol T, Iezzi G, Perrotti V, Di Stefano DA. Sinus augmentation and concomitant implant placement in low bone-density sites. A retrospective study on an undersized drilling protocol and primary stability. Clin Implant Dent Relat Res. 2018 Apr;20(2):151-159.
16. Artzi Z, Kohen J, Carmeli G, Karmon B, Lor A, Ormianer Z. The efficacy of full-arch immediately restored implant-supported reconstructions in extraction and healed sites: a 36-month retrospective evaluation. Int J Oral Maxillofac Implants. 2010 Mar-Apr;25(2):329-35.
17. Arunjaroensuk S, Panmekiate S, Pimkhaokham A. The Stability of Augmented Bone Between Two Different Membranes Used for Guided Bone Regeneration Simultaneous with Dental Implant Placement in the Esthetic Zone. Int J Oral Maxillofac Implants. 2018 January/February;33(1):206–216.
18. Atieh MA, Alsabeeha NH, Payne AG, de Silva RK, Schwass DS, Duncan WJ. The prognostic accuracy of resonance frequency analysis in predicting failure risk of immediately restored implants. Clin Oral Implants Res. 2014 Jan;25(1):29-35.
19. Babbush CA, Brokloff J. A single-center retrospective analysis of 1001 consecutively placed NobelActive implants. Implant Dent. 2012 Feb;21(1):28-35.
20. Bae OY, Kim YS, Shin SY, Kim WK, Lee YK, Kim SH. Clinical Outcomes of Reamer- vs Osteotome-Mediated Sinus Floor Elevation with Simultaneous Implant Placement: A 2-Year Retrospective Study. Int J Oral Maxillofac Implants. 2015 Jul-Aug;30(4):925-30.
21. Bahat O, Handelsman M. Use of wide implants and double implants in the posterior jaw: a clinical report. Int J Oral Maxillofac Implants. 1996 May-Jun;11(3):379-86.

22. Bahat O. Brånemark system implants in the posterior maxilla: clinical study of 660 implants followed for 5 to 12 years. Int J Oral Maxillofac Implants. 2000 Sep-Oct;15(5):646-53.
23. Bahat O. Osseointegrated implants in the maxillary tuberosity: report on 45 consecutive patients. Int J Oral Maxillofac Implants. 1992 Winter;7(4):459-67.
24. Bain CA, Moy PK. The association between the failure of dental implants and cigarette smoking. Int J Oral Maxillofac Implants. 1993;8(6):609-15.
25. Balleri P, Cozzolino A, Ghelli L, Momicchioli G, Varriale A. Stability measurements of osseointegrated implants using Osstell in partially edentulous jaws after 1 year of loading: a pilot study. Clin Implant Dent Relat Res. 2002;4(3):128-32.
26. Balleri P, Ferrari M, Veltri M. One-year outcome of implants strategically placed in the retrocanine bone triangle. Clin Implant Dent Relat Res. 2010 Dec;12(4):324-30.
27. Balshi SF, Wolfinger GJ, Balshi TJ. A prospective study of immediate functional loading, following the Teeth in a Day protocol: a case series of 55 consecutive edentulous maxillas. Clin Implant Dent Relat Res. 2005;7(1):24-31.
28. Balshi TJ, Ekfeldt A, Stenberg T, Vrielinck L. Three-year evaluation of Brånemark implants connected to angulated abutments. Int J Oral Maxillofac Implants. 1997 Jan-Feb;12(1):52-8.
29. Balshi TJ, Wolfinger GJ, Balshi SF 2nd. Analysis of 356 pterygomaxillary implants in edentulous arches for fixed prosthesis anchorage. Int J Oral Maxillofac Implants. 1999 May-Jun;14(3):398-406.
30. Balshi TJ, Wolfinger GJ, Stein BE, Balshi SF. A long-term retrospective analysis of survival rates of implants in the mandible. Int J Oral Maxillofac Implants. 2015 Nov-Dec;30(6):1348-54.
31. Bechara S, Kubilius R, Veronesi G, Pires JT, Shibli JA, Mangano FG. Short (6-mm) dental implants versus sinus floor elevation and placement of longer (≥10-mm) dental implants: a randomized controlled trial with a 3-year follow-up. Clin Oral Implants Res. 2017 Sep;28(9):1097-1107.
32. Becker W, Becker BE, Hujoel P, Abu Ras Z, Goldstein M, Smidt A. Prospective clinical trial evaluating a new implant system for implant survival, implant stability and radiographic bone changes. Clin Implant Dent Relat Res. 2013 Feb;15(1):15-21.
33. Becker W, Becker BE, Ricci A, Bahat O, Rosenberg E, Rose LF, Handelsman M, Israelson H. A prospective multicenter clinical trial comparing one- and two-stage titanium screw-shaped fixtures with one-stage plasma-sprayed solid-screw fixtures. Clin Implant Dent Relat Res. 2000;2(3):159-65.
34. Becker W, Goldstein M, Becker BE, Sennerby L. Minimally invasive flapless implant surgery: a prospective multicenter study. Clin Implant Dent Relat Res. 2005;7 Suppl 1:S21-7.
35. Becktor JP, Eckert SE, Isaksson S, Keller EE. The influence of mandibular dentition on implant failures in bone-grafted edentulous maxillae. Int J Oral Maxillofac Implants. 2002 Jan-Feb;17(1):69-77.
36. Becktor JP, Isaksson S, Billström C. A prospective multicenter study using two different surgical approaches in the mandible with turned Brånemark implants: conventional loading using fixed prostheses. Clin Implant Dent Relat Res. 2007 Dec;9(4):179-85.
37. Becktor JP, Isaksson S, Sennerby L. Survival analysis of endosseous implants in grafted and nongrafted edentulous maxillae. Int J Oral Maxillofac Implants. 2004 Jan-Feb;19(1):107-15.
38. Benlidayi ME, Ucar Y, Tatli U, Ekren O, Evlice B, Kisa HI, Baksi U. Short Implants Versus Standard Implants: Midterm Outcomes of a Clinical Study. Implant Dent. 2018 Feb;27(1):95-100.
39. Bergendal T, Engquist B. Implant-supported overdentures: a longitudinal prospective study. Int J Oral Maxillofac Implants. 1998 Mar-Apr;13(2):253-62.
40. Bernard L, Vercruyssen M, Duyck J, Jacobs R, Teughels W, Quirynen M. A randomized controlled clinical trial comparing guided with nonguided implant placement: A 3-year follow-up of implant-centered outcomes. J Prosthet Dent. 2019 Jun;121(6):904-910.
41. Bernardi S, Gatto R, Severino M, Botticelli G, Caruso S, Rastelli C, Lupi E, Roias AQ, Iacomino E, Falisi G. Short Versus Longer Implants in Mandibular Alveolar Ridge Augmented Using Osteogenic Distraction: One-Year Follow-up of a Randomized Split-Mouth Trial. J Oral Implantol. 2018 Jun;44(3):184-191.
42. Bianco G, Di Raimondo R, Luongo G, Paoleschi C, Piccoli P, Piccoli C, Rangert B. Osseointegrated implant for single-tooth replacement: a retrospective multicenter study on routine use in private practice. Clin Implant Dent Relat Res. 2000;2(3):152-8.
43. Bischof M, Nedir R, Abi Najm S, Szmukler-Moncler S, Samson J. A five-year life-table analysis on wide neck ITI implants with prosthetic evaluation and radiographic analysis: results from a private practice. Clin Oral Implants Res. 2006 Oct;17(5):512-20.
44. Blomqvist JE, Alberius P, Isaksson S. Two-stage maxillary sinus reconstruction with endosseous implants: a prospective study. Int J Oral Maxillofac Implants. 1998 Nov-Dec;13(6):758-66.
45. Blus C, Szmukler-Moncler S. Split-crest and immediate implant placement with ultra-sonic bone surgery: a 3-year life-table analysis with 230 treated sites. Clin Oral Implants Res. 2006 Dec;17(6):700-7.

46. Bogaerde L, Pedretti G, Dellacasa P, Mozzati M, Rangert B, Wendelhag I. Early function of splinted implants in maxillas and posterior mandibles, using Brånemark System Tiunite implants: an 18-month prospective clinical multicenter study. Clin Implant Dent Relat Res. 2004;6(3):121-9.
47. Bogaerde L, Pedretti G, Dellacasa P, Mozzati M, Rangert B. Early function of splinted implants in maxillas and posterior mandibles using Brånemark system machined-surface implants: an 18-month prospective clinical multicenter study. Clin Implant Dent Relat Res. 2003;5 Suppl 1:21-8.
48. Bogaerde L, Rangert B, Wendelhag I. Immediate/early function of Brånemark System TiUnite implants in fresh extraction sockets in maxillae and posterior mandibles: an 18-month prospective clinical study. Clin Implant Dent Relat Res. 2005;7 Suppl 1:S121-30.
49. Bolle C, Felice P, Barausse C, Pistilli V, Trullenque-Eriksson A, Esposito M. 4 mm long vs longer implants in augmented bone in posterior atrophic jaws: 1-year post-loading results from a multicentre randomised controlled trial. Eur J Oral Implantol. 2018;11(1):31-47.
50. Boon L, De Mars G, Favril C, Duyck J, Quirynen M, Vandamme K. Esthetic evaluation of single implant restorations, adjacent single implant restorations, and implant-supported fixed partial dentures: A 1-year prospective study. Clin Implant Dent Relat Res. 2020 Feb;22(1):128-137.
51. Borges T, Fernandes D, Almeida B, Pereira M, Martins D, Azevedo L, Marques T. Correlation between alveolar bone morphology and volumetric dimensional changes in immediate maxillary implant placement: A 1-year prospective cohort study. J Periodontol. 2020 Sep;91(9):1167-1176.
52. Bornstein MM, Halbritter S, Harnisch H, Weber HP, Buser D. A retrospective analysis of patients referred for implant placement to a specialty clinic: indications, surgical procedures, and early failures. Int J Oral Maxillofac Implants. 2008 Nov-Dec;23(6):1109-16.
53. Bouhy A, Rompen E, Lamy M, Legros C, Lecloux G, Lambert F. Maxillary implant overdenture retained by four unsplinted attachments and opposed by a natural or fixed dentition: One-year clinical outcomes. Clin Oral Implants Res. 2020 Aug;31(8):747-767.
54. Brånemark PI, Svensson B, van Steenberghe D. Ten-year survival rates of fixed prostheses on four or six implants ad modum Brånemark in full edentulism. Clin Oral Implants Res. 1995 Dec;6(4):227-31.
55. Bressan E, Lops D. Conometric retention for complete fixed prosthesis supported by four implants: 2-years prospective study. Clin Oral Implants Res. 2014 May;25(5):546-52.
56. Browaeys H, Defrancq J, Dierens MC, Miremadi R, Vandeweghe S, Van de Velde T, De Bruyn H. A retrospective analysis of early and immediately loaded osseotite implants in cross-arch rehabilitations in edentulous maxillas and mandibles up to 7 years. Clin Implant Dent Relat Res. 2013 Jun;15(3):380-9.
57. Brügger OE, Bornstein MM, Kuchler U, Janner SF, Chappuis V, Buser D. Implant therapy in a surgical specialty clinic: an analysis of patients, indications, surgical procedures, risk factors, and early failures. Int J Oral Maxillofac Implants. 2015 Jan-Feb;30(1):151-60.
58. Buser D, Mericske-Stern R, Bernard JP, Behneke A, Behneke N, Hirt HP, Belser UC, Lang NP. Long-term evaluation of non-submerged ITI implants. Part 1: 8-year life table analysis of a prospective multi-center study with 2359 implants. Clin Oral Implants Res. 1997 Jun;8(3):161-72.
59. Caban J, Fermergård R, Abtahi J. Long-term evaluation of osteotome sinus floor elevation and simultaneous placement of implants without bone grafts: 10-Year radiographic and clinical follow-up. Clin Implant Dent Relat Res. 2017 Dec;19(6):1023-1033.
60. Calandriello R, Tomatis M. Immediate occlusal loading of single lower molars using Brånemark System® Wide Platform TiUnite™ implants: a 5-year follow-up report of a prospective clinical multicenter study. Clin Implant Dent Relat Res. 2011 Dec;13(4):311-8.
61. Calvo-Guirado JL, López Torres JA, Dard M, Javed F, Pérez-Albacete Martínez C, Maté Sánchez de Val JE. Evaluation of extrashort 4-mm implants in mandibular edentulous patients with reduced bone height in comparison with standard implants: a 12-month results. Clin Oral Implants Res. 2016 Jul;27(7):867-874.
62. Campos CG, Francischone CE, Souza Picorelli Assis NM, Devito KL, Sotto-Maior BS. Neurosensory Function and Implant Survival Rate Following Implant Placement With or Without an Interposed Bone Graft Between the Implant and Nerve: Prospective Clinical Trial. Int J Oral Maxillofac Implants. 2019 Nov/Dec;33(6):1450-1456.
63. Cannizzaro G, Felice P, Ippolito DR, Velasco-Ortega E, Esposito M. Immediate loading of fixed cross-arch prostheses supported by flapless-placed 5 mm or 11.5 mm long implants: 5-year results from a randomised controlled trial. Eur J Oral Implantol. 2018;11(3):295-306
64. Cannizzaro G, Felice P, Minciarelli AF, Leone M, Viola P, Esposito M. Early implant loading in the atrophic posterior maxilla: 1-stage lateral versus crestal sinus lift and 8 mm hydroxyapatite-coated implants. A 5-year randomised controlled trial. Eur J Oral Implantol. 2013 Spring;6(1):13-25.

65. Cannizzaro G, Leone M, Ferri V, Viola P, Gelpi F, Esposito M. Immediate loading of single implants inserted flapless with medium or high insertion torque: a 6-month follow-up of a split-mouth randomised controlled trial. Eur J Oral Implantol. 2012 Winter;5(4):333-42.
66. Cannizzaro G, Loi I, Viola P, Ferri V, Leone M, Trullenque-Eriksson A, Esposito M. Immediate loading of two (fixed-on-2) versus three (fixed-on-3) implants placed flapless supporting cross-arch fixed prostheses: One-year results from a randomised controlled trial. Eur J Oral Implantol. 2016;9 Suppl 1(2):143-53.
67. Carr AB, Arwani N, Lohse CM, Gonzalez RLV, Muller OM, Salinas TJ. Early Implant Failure Associated With Patient Factors, Surgical Manipulations, and Systemic Conditions. J Prosthodont. 2019 Jul;28(6):623-633.
68. Carr AB, Choi YG, Eckert SE, Desjardins RP. Retrospective cohort study of the clinical performance of 1-stage dental implants. Int J Oral Maxillofac Implants. 2003 May-Jun;18(3):399-405.
69. Cecchinato D, Lops D, Salvi GE, Sanz M. A prospective, randomized, controlled study using OsseoSpeed™ implants placed in maxillary fresh extraction socket: soft tissues response. Clin Oral Implants Res. 2015;26(1):20-7.
70. Checchi L, Felice P, Antonini ES, Cosci F, Pellegrino G, Esposito M. Crestal sinus lift for implant rehabilitation: a randomised clinical trial comparing the Cosci and the Summers techniques. A preliminary report on complications and patient preference. Eur J Oral Implantol. 2010 Autumn;3(3):221-32.
71. Chiapasco M, Casentini P, Zaniboni M. Implants in reconstructed bone: a comparative study on the outcome of Straumann® tissue level and bone level implants placed in vertically deficient alveolar ridges treated by means of autogenous onlay bone grafts. Clin Implant Dent Relat Res. 2014 Feb;16(1):32-50.
72. Clelland N, Chaudhry J, Rashid RG, McGlumphy E. Split-Mouth Comparison of Splinted and Nonsplinted Prostheses on Short Implants: 3-Year Results. Int J Oral Maxillofac Implants. 2016 Sep-Oct;31(5):1135-41.
73. Cochran D, Oates T, Morton D, Jones A, Buser D, Peters F. Clinical field trial examining an implant with a sand-blasted, acid-etched surface. J Periodontol. 2007 Jun;78(6):974-82.
74. Cochran DL, Buser D, ten Bruggenkate CM, Weingart D, Taylor TM, Bernard JP, Peters F, Simpson JP. The use of reduced healing times on ITI implants with a sandblasted and acid-etched (SLA) surface: early results from clinical trials on ITI SLA implants. Clin Oral Implants Res. 2002 Apr;13(2):144-53.
75. Collaert B, De Bruyn H. Early loading of four or five Astra Tech fixtures with a fixed cross-arch restoration in the mandible. Clin Implant Dent Relat Res. 2002;4(3):133-5.
76. Collaert B, Wijnen L, De Bruyn H. A 2-year prospective study on immediate loading with fluoride-modified implants in the edentulous mandible. Clin Oral Implants Res. 2011 Oct;22(10):1111-1116.
77. Correia F, Gouveia S, Felino AC, Costa AL, Almeida RF. Survival Rate of Dental Implants in Patients with History of Periodontal Disease: A Retrospective Cohort Study. Int J Oral Maxillofac Implants. 2017 Jul/Aug;32(4):927-934.
78. Cosyn J, Vandenbulcke E, Browaeys H, Van Maele G, De Bruyn H. Factors associated with failure of surface-modified implants up to four years of function. Clin Implant Dent Relat Res. 2012 Jun;14(3):347-58.
79. Cucchi A, Vignudelli E, Franco S, Ghensi P, Malchiodi L, Corinaldesi G. Evaluation of Crestal Bone Loss Around Straight and Tilted Implants in Patients Rehabilitated by Immediate-Loaded Full-Arch All-on-4 or All-on-6: A Prospective Study. J Oral Implantol. 2019 Dec;45(6):434-443.
80. Dagorne C, Malet J, Bizouard G, Mora F, Rangé H, Bouchard P. Clinical evaluation of two dental implant macrostructures on peri-implant bone loss: a comparative, retrospective study. Clin Oral Implants Res. 2015 Mar;26(3):307-13.
81. Dahlin C, Widmark G, Bergkvist G, Fürst B, Widbom T, Kashani H. One-year results of a clinical and radiological prospective multicenter study on NEOSS® dental implants. Clin Implant Dent Relat Res. 2013 Apr;15(2):303-8.
82. Davarpanah M, Martinez H, Etienne D, Zabalegui I, Mattout P, Chiche F, Michel JF. A prospective multicenter evaluation of 1,583 3i implants: 1- to 5-year data. Int J Oral Maxillofac Implants. 2002 Nov-Dec;17(6):820-8.
83. De Bruyn H, Collaert B, Lindén U, Flygare L. A comparative study of the clinical efficacy of Screw Vent implants versus Brånemark fixtures, installed in a periodontal clinic. Clin Oral Implants Res. 1992 Mar;3(1):32-41.
84. De Bruyn H, Collaert B. Early loading of machined-surface Brånemark implants in completely edentulous mandibles: healed bone versus fresh extraction sites. Clin Implant Dent Relat Res. 2002;4(3):136-42.
85. Degasperi W, Andersson P, Verrocchi D, Sennerby L. One-year clinical and radiographic results with a novel hydrophilic titanium dental implant. Clin Implant Dent Relat Res. 2014 Aug;16(4):511-9.
86. Degidi M, Daprile G, Piattelli A. Primary stability determination by means of insertion torque and RFA in a sample of 4,135 implants. Clin Implant Dent Relat Res. 2012 Aug;14(4):501-7.
87. Degidi M, Iezzi G, Perrotti V, Piattelli A. Comparative analysis of immediate functional loading and immediate nonfunctional loading to traditional healing periods: a 5-year follow-up of 550 dental implants. Clin Implant Dent Relat Res. 2009 Dec;11(4):257-66.
88. Degidi M, Piattelli A, Carinci F. Parallel screw cylinder implants: comparative analysis between immediate loading and two-stage healing of 1,005 dental implants with a 2-year follow up. Clin Implant Dent Relat Res. 2006;8(3):151-60.

89. Degidi M, Piattelli A. Comparative analysis study of 702 dental implants subjected to immediate functional loading and immediate nonfunctional loading to traditional healing periods with a follow-up of up to 24 months. Int J Oral Maxillofac Implants. 2005 Jan-Feb;20(1):99-107.
90. Deporter D, Pharoah M, Yeh S, Todescan R, Atenafu EG. Performance of titanium alloy sintered porous-surfaced (SPS) implants supporting mandibular overdentures during a 20-year prospective study. Clin Oral Implants Res. 2014 Feb;25(2):e189-95.
91. Deporter DA, Todescan R, Watson PA, Pharoah M, Pilliar RM, Tomlinson G. A prospective human clinical trial of Endopore dental implants in restoring the partially edentulous maxilla using fixed prostheses. Int J Oral Maxillofac Implants. 2001 Jul-Aug;16(4):527-36.
92. Díaz-Sánchez RM, Delgado-Muñoz JM, Hita-Iglesias P, Pullen KT, Serrera-Figallo MÁ, Torres-Lagares D. Improvement in the Initial Implant Stability Quotient Through Use of a Modified Surgical Technique. J Oral Implantol. 2017 Jun;43(3):186-193.
93. Ducommun J, El Kholy K, Rahman L, Schimmel M, Chappuis V, Buser D. Analysis of trends in implant therapy at a surgical specialty clinic: Patient pool, indications, surgical procedures, and rate of early failures-A 15-year retrospective analysis. Clin Oral Implants Res. 2019 Nov;30(11):1097-1106.
94. Eckert SE, Meraw SJ, Weaver AL, Lohse CM. Early experience with Wide-Platform Mk II implants. Part I: Implant survival. Part II: Evaluation of risk factors involving implant survival. Int J Oral Maxillofac Implants. 2001 Mar-Apr;16(2):208-16.
95. Eliasson A, Blomqvist F, Wennerberg A, Johansson A. A retrospective analysis of early and delayed loading of full-arch mandibular prostheses using three different implant systems: clinical results with up to 5 years of loading. Clin Implant Dent Relat Res. 2009 Jun;11(2):134-48.
96. Engstrand P, Gröndahl K, Ohrnell LO, Nilsson P, Nannmark U, Brånemark PI. Prospective follow-up study of 95 patients with edentulous mandibles treated according to the Brånemark Novum concept. Clin Implant Dent Relat Res. 2003;5(1):3-10.
97. Esposito M, Barausse C, Pistilli R, Piattelli M, Di Simone S, Ippolito DR, Felice P. Posterior atrophic jaws rehabilitated with prostheses supported by 5 × 5 mm implants with a nanostructured calcium-incorporated titanium surface or by longer implants in augmented bone. Five-year results from a randomised controlled trial. Int J Oral Implantol (Berl). 2019;12(1):39-54.
98. Esposito M, Barausse C, Pistilli R, Sammartino G, Grandi G, Felice P. Short implants versus bone augmentation for placing longer implants in atrophic maxillae: One-year post-loading results of a pilot randomised controlled trial. Eur J Oral Implantol. 2015 Autumn;8(3):257-68.
99. Esposito M, Dojcinovic I, Buchini S, Péchy P, Aronsson BO. Safety and efficacy of a biomimetic monolayer of permanently bound multiphosphonic acid molecules on dental implants: 3 years post-loading results from a pilot quadruple-blinded randomised controlled trial. Eur J Oral Implantol. 2017;10(1):43-54.
100. Esposito M, Maghaireh H, Pistilli R, Grusovin MG, Lee ST, Trullenque-Eriksson A, Gualini F. Dental implants with internal versus external connections: 5-year post-loading results from a pragmatic multicenter randomised controlled trial. Eur J Oral Implantol. 2016;9 Suppl 1(2):129-41.
101. Evian CI. A comparison of hydroxyapatite-coated Micro-Vent and pure titanium Swede-Vent implants. Int J Oral Maxillofac Implants. 1996 Sep-Oct;11(5):639-44.
102. Felice P, Barausse C, Blasone R, Favaretto G, Stacchi C, Calvo M, Marin C, Buti J, Esposito M. A comparison of two dental implant systems in partially edentulous patients: 1-year post-loading results from a pragmatic multicentre randomised controlled trial. Eur J Oral Implantol. 2014 Winter;7(4):397-409.
103. Felice P, Barausse C, Pistilli R, Ippolito DR, Esposito M. Five-year results from a randomised controlled trial comparing prostheses supported by 5-mm long implants or by longer implants in augmented bone in posterior atrophic edentulous jaws. Int J Oral Implantol (Berl). 2019;12(1):25-37.
104. Felice P, Pistilli R, Barausse C, Piattelli M, Buti J, Esposito M. Posterior atrophic jaws rehabilitated with prostheses supported by 6-mm-long 4-mm-wide implants or by longer implants in augmented bone. Five-year post-loading results from a within-person randomised controlled trial. Int J Oral Implantol (Berl). 2019;12(1):57-72.
105. Felice P, Pistilli R, Barausse C, Trullenque-Eriksson A, Esposito M. Immediate non-occlusal loading of immediate post-extractive versus delayed placement of single implants in preserved sockets of the anterior maxilla: 1-year post-loading outcome of a randomised controlled trial. Eur J Oral Implantol. 2015 Winter;8(4):361-72.
106. Feng Y, Tang Y, Liu Y, Chen F, Li D. Maxillary sinus floor elevation using the osteotome technique in the presence of antral pseudocysts: a retrospective study with an average follow-up of 27 months. Int J Oral Maxillofac Implants. 2014 Mar-Apr;29(2):408-13.
107. Fenlon MR, Palmer RM, Palmer P, Newton JT, Sherriff M. A prospective study of single stage surgery for implant supported overdentures. Clin Oral Implants Res. 2002 Aug;13(4):365-70.
108. Fermergård R, Åstrand P. Osteotome sinus floor elevation without bone grafts--a 3-year retrospective study with Astra Tech implants. Clin Implant Dent Relat Res. 2012 Apr;14(2):198-205.

109. Ferrigno N, Laureti M, Fanali S, Grippaudo G. A long-term follow-up study of non-submerged ITI implants in the treatment of totally edentulous jaws. Part I: Ten-year life table analysis of a prospective multicenter study with 1286 implants. Clin Oral Implants Res. 2002 Jun;13(3):260-73.
110. Ferrigno N, Laureti M, Fanali S. Dental implants placement in conjunction with osteotome sinus floor elevation: a 12-year life-table analysis from a prospective study on 588 ITI implants. Clin Oral Implants Res. 2006 Apr;17(2):194-205.
111. Fischer K, Stenberg T, Hedin M, Sennerby L. Five-year results from a randomized, controlled trial on early and delayed loading of implants supporting full-arch prosthesis in the edentulous maxilla. Clin Oral Implants Res. 2008 May;19(5):433-41.
112. Fortin Y, Sullivan RM, Rangert BR. The Marius implant bridge: surgical and prosthetic rehabilitation for the completely edentulous upper jaw with moderate to severe resorption: a 5-year retrospective clinical study. Clin Implant Dent Relat Res. 2002;4(2):69-77.
113. Fortin Y, Sullivan RM. Terminal Posterior Tilted Implants Planned as a Sinus Graft Alternative for Fixed Full-Arch Implant-Supported Maxillary Restoration: A Case Series with 10- to 19-Year Results on 44 Consecutive Patients Presenting for Routine Maintenance. Clin Implant Dent Relat Res. 2017 Feb;19(1):56-68.
114. Friberg B, Dahlin C, Widmark G, Ostman PO, Billström C. One-year results of a prospective multicenter study on Brånemark System implants with a TiUnite surface. Clin Implant Dent Relat Res. 2005;7 Suppl 1:S70-5.
115. Friberg B, Ekestubbe A, Sennerby L. Clinical outcome of Brånemark System implants of various diameters: a retrospective study. Int J Oral Maxillofac Implants. 2002 Sep-Oct;17(5):671-7.
116. Friberg B, Grondahl K, Lekholm U. A new self-tapping Brånemark implant: clinical and radiographic evaluation. Int J Oral Maxillofac Implants. 1992 Spring;7(1):80-5.
117. Friberg B, Jemt T. Turned Brånemark System implants in wide and narrow edentulous maxillae: a retrospective clinical study. Clin Implant Dent Relat Res. 2008 May;10(2):78-85.
118. Friberg B, Jisander S, Widmark G, Lundgren A, Ivanoff CJ, Sennerby L, Thorén C. One-year prospective three-center study comparing the outcome of a "soft bone implant" (prototype Mk IV) and the standard Brånemark implant. Clin Implant Dent Relat Res. 2003;5(2):71-7.
119. Friberg B, Nilson H, Olsson M, Palmquist C. Mk II: the self-tapping Brånemark implant: 5-year results of a prospective 3-center study. Clin Oral Implants Res. 1997 Aug;8(4):279-85.
120. Fugazzotto PA. Immediate implant placement following a modified trephine/osteotome approach: success rates of 116 implants to 4 years in function. Int J Oral Maxillofac Implants. 2002 Jan-Feb;17(1):113-20.
121. Gallucci GO, Bernard JP, Bertosa M, Belser UC. Immediate loading with fixed screw-retained provisional restorations in edentulous jaws: the pickup technique. Int J Oral Maxillofac Implants. 2004 Jul-Aug;19(4):524-33.
122. Gallucci GO, Doughtie CB, Hwang JW, Fiorellini JP, Weber HP. Five-year results of fixed implant-supported rehabilitations with distal cantilevers for the edentulous mandible. Clin Oral Implants Res. 2009 Jun;20(6):601-7.
123. Garcez-Filho J, Tolentino L, Sukekava F, Seabra M, Cesar-Neto JB, Araújo MG. Long-term outcomes from implants installed by using split-crest technique in posterior maxillae: 10 years of follow-up. Clin Oral Implants Res. 2015 Mar;26(3):326-31.
124. Garlini G, Bianchi C, Chierichetti V, Sigurtà D, Maiorana C, Santoro F. Retrospective clinical study of Osseotite implants: zero- to 5-year results. Int J Oral Maxillofac Implants. 2003 Jul-Aug;18(4):589-93.
125. Gastaldi G, Felice P, Pistilli R, Barausse C, Trullenque-Eriksson A, Esposito M. Short implants as an alternative to crestal sinus lift: a 3-year multicentre randomised controlled trial. Eur J Oral Implantol. 2017;10(4):391-400.
126. Geckili O, Bilhan H, Geckili E, Cilingir A, Mumcu E, Bural C. Evaluation of possible prognostic factors for the success, survival, and failure of dental implants. Implant Dent. 2014 Feb;23(1):44-50.
127. Gholami H, Mericske-Stern R, Kessler-Liechti G, Katsoulis J. Radiographic bone level changes of implant-supported restorations in edentulous and partially dentate patients: 5-year results. Int J Oral Maxillofac Implants. 2014 Jul-Aug;29(4):898-904.
128. Glauser R, Lundgren AK, Gottlow J, Sennerby L, Portmann M, Ruhstaller P, Hämmerle CH. Immediate occlusal loading of Brånemark TiUnite implants placed predominantly in soft bone: 1-year results of a prospective clinical study. Clin Implant Dent Relat Res. 2003;5 Suppl 1:47-56.
129. Glauser R, Rée A, Lundgren A, Gottlow J, Hämmerle CH, Schärer P. Immediate occlusal loading of Brånemark implants applied in various jawbone regions: a prospective, 1-year clinical study. Clin Implant Dent Relat Res. 2001;3(4):204-13.
130. Glibert M, Vervaeke S, De Bruyn H, Östman PO. Clinical and Radiographic Comparison between Platform-Shifted and Nonplatform-Shifted Implant: A One-Year Prospective Study. Clin Implant Dent Relat Res. 2016 Feb;18(1):129-37.
131. Golab KG, Balouch A, Mirtorabi S. One-Year Multicenter Prospective Evaluation of Survival Rates and Bone Resorption in One-Piece Implants. Clin Implant Dent Relat Res. 2016 Apr;18(2):392-400.
132. Gopalakrishnan D, Joshi V, Romanos GE. Soft and hard tissue changes around laser microtexture single tooth implants--a clinical and radiographic evaluation. Implant Dent. 2014 Oct;23(5):570-5.
133. Gotfredsen K, Holm B, Sewerin I, Harder F, Hjörting-Hansen E, Pedersen CS, Christensen K. Marginal tissue response adjacent to Astra Dental Implants supporting overdentures in the mandible. Clin Oral Implants Res. 1993 Jun;4(2):83-9.

134. Goto M, Jin-Nouchi S, Ihara K, Katsuki T. Longitudinal follow-up of osseointegrated implants in patients with resected jaws. Int J Oral Maxillofac Implants. 2002 Mar-Apr;17(2):225-30.
135. Grunder U, Polizzi G, Goené R, Hatano N, Henry P, Jackson WJ, Kawamura K, Köhler S, Renouard F, Rosenberg R, Triplett G, Werbitt M, Lithner B. A 3-year prospective multicenter follow-up report on the immediate and delayed-immediate placement of implants. Int J Oral Maxillofac Implants. 1999 Mar-Apr;14(2):210-6.
136. Guarnieri R, Belleggia F, Grande M. Immediate versus Delayed Treatment in the Anterior Maxilla Using Single Implants with a Laser-Microtextured Collar: 3-Year Results of a Case Series on Hard- and Soft-Tissue Response and Esthetics. J Prosthodont. 2016 Feb;25(2):135-45.
137. Guarnieri R, Di Nardo D, Di Giorgio G, Miccoli G, Testarelli L. Clinical and radiographics results at 3 years of RCT with split-mouth design of submerged vs. nonsubmerged single laser-microgrooved implants in posterior areas. Int J Implant Dent. 2019 Dec 18;5(1):44.
138. Guarnieri R, Di Nardo D, Gaimari G, Miccoli G, Testarelli L. Short vs. Standard Laser-Microgrooved Implants Supporting Single and Splinted Crowns: A Prospective Study with 3 Years Follow-Up. J Prosthodont. 2019 Feb;28(2):e771-e779.
139. Guarnieri R, Placella R, Testarelli L, Iorio-Siciliano V, Grande M. Clinical, radiographic, and esthetic evaluation of immediately loaded laser microtextured implants placed into fresh extraction sockets in the anterior maxilla: a 2-year retrospective multicentric study. Implant Dent. 2014 Apr;23(2):144-54.
140. Guida L, Annunziata M, Esposito U, Sirignano M, Torrisi P, Cecchinato D. 6-mm-short and 11-mm-long implants compared in the full-arch rehabilitation of the edentulous mandible: A 3-year multicenter randomized controlled trial. Clin Oral Implants Res. 2020 Jan;31(1):64-73.
141. Guljé F, Abrahamsson I, Chen S, Stanford C, Zadeh H, Palmer R. Implants of 6 mm vs. 11 mm lengths in the posterior maxilla and mandible: a 1-year multicenter randomized controlled trial. Clin Oral Implants Res. 2013 Dec;24(12):1325-31.
142. Guljé FL, Raghoebar GM, Vissink A, Meijer HJA. Single crowns in the resorbed posterior maxilla supported by either 11-mm implants combined with sinus floor elevation or 6-mm implants:A 5-year randomised controlled trial. Int J Oral Implantol (Berl). 2019;12(3):315-326.
143. Haas R, Mensdorff-Pouilly N, Mailath G, Watzek G. Survival of 1,920 IMZ implants followed for up to 100 months. Int J Oral Maxillofac Implants. 1996 Sep-Oct;11(5):581-8.
144. Hallman M, Mordenfeld A, Strandkvist T. A retrospective 5-year follow-up study of two different titanium implant surfaces used after interpositional bone grafting for reconstruction of the atrophic edentulous maxilla. Clin Implant Dent Relat Res. 2005;7(3):121-6.
145. Hallman M, Nordin T. Sinus floor augmentation with bovine hydroxyapatite mixed with fibrin glue and later placement of nonsubmerged implants: a retrospective study in 50 patients. Int J Oral Maxillofac Implants. 2004 Mar-Apr;19(2):222-7.
146. Hallman M. A prospective study of treatment of severely resorbed maxillae with narrow nonsubmerged implants: results after 1 year of loading. Int J Oral Maxillofac Implants. 2001 Sep-Oct;16(5):731-6.
147. Han HJ, Kim S, Han DH. Multifactorial evaluation of implant failure: a 19-year retrospective study. Int J Oral Maxillofac Implants. 2014 Mar-Apr;29(2):303-10.
148. Hatano N, Yamaguchi M, Yaita T, Ishibashi T, Sennerby L. New approach for immediate prosthetic rehabilitation of the edentulous mandible with three implants: a retrospective study. Clin Oral Implants Res. 2011 Nov;22(11):1265-9.
149. Hattingh A, De Bruyn H, Vandeweghe S. A retrospective study on ultra-wide diameter dental implants for immediate molar replacement. Clin Implant Dent Relat Res. 2019 Oct;21(5):879-887.
150. Hayacibara RM, Gonçalves CS, Garcez-Filho J, Magro-Filho O, Esper H, Hayacibara MF. The success rate of immediate implant placement of mandibular molars: a clinical and radiographic retrospective evaluation between 2 and 8 years. Clin Oral Implants Res. 2013 Jul;24(7):806-11.
151. He J, Zhao B, Deng C, Shang D, Zhang C. Assessment of implant cumulative survival rates in sites with different bone density and related prognostic factors: an 8-year retrospective study of 2,684 implants. Int J Oral Maxillofac Implants. 2015 Mar-Apr;30(2):360-71.
152. Hentenaar DF, De Waal YC, Van Winkelhoff AJ, Raghoebar GM, Meijer HJ. Influence of Cervical Crown Contour on Marginal Bone Loss Around Platform-Switched Bone-Level Implants: A 5-Year Cross-Sectional Study. Int J Prosthodont. 2020 Jul/Aug;33(4):373-379.
153. Hentschel A, Herrmann J, Glauche I, Vollmer A, Schlegel KA, Lutz R. Survival and patient satisfaction of short implants during the first 2 years of function: a retrospective cohort study with 694 implants in 416 patients. Clin Oral Implants Res. 2016 May;27(5):591-6.
154. Herrero-Climent M, Romero Ruiz MM, Díaz-Castro CM, Bullón P, Ríos-Santos JV. Influence of two different machined-collar heights on crestal bone loss. Int J Oral Maxillofac Implants. 2014 Nov-Dec;29(6):1374-9.
155. Herrmann I, Lekholm U, Holm S, Kultje C. Evaluation of patient and implant characteristics as potential prognostic factors for oral implant failures. Int J Oral Maxillofac Implants. 2005 Mar-Apr;20(2):220-30.
156. Higuchi KW, Folmer T, Kultje C. Implant survival rates in partially edentulous patients: a 3-year prospective multicenter study. J Oral Maxillofac Surg. 1995 Mar;53(3):264-8.

157. Hoffmann O, Beaumont C, Tatakis DN, Zafiropoulos GG. Telescopic crowns as attachments for implant supported restorations: a case series. J Oral Implantol. 2006;32(6):291-9.
158. Hopp M, de Araújo Nobre M, Maló P. Comparison of marginal bone loss and implant success between axial and tilted implants in maxillary All-on-4 treatment concept rehabilitations after 5 years of follow-up. Clin Implant Dent Relat Res. 2017 Oct;19(5):849-859.
159. Horikawa T, Odatsu T, Itoh T, Soejima Y, Morinaga H, Abe N, Tsuchiya N, Iijima T, Sawase T. Retrospective cohort study of rough-surface titanium implants with at least 25 years' function. Int J Implant Dent. 2017 Sep 5;3(1):42.
160. Hu C, Lang NP, Ong MM, Lim LP, Tan WC. Influence of periodontal maintenance and periodontitis susceptibility on implant success: A 5-year retrospective cohort on moderately rough surfaced implants. Clin Oral Implants Res. 2020 Aug;31(8):727-736.
161. Hussaini S, Weiner S, Ahmad M. Implant survival rates in a condensed surgical and prosthetic training program for general practitioners in dental implants. Implant Dent. 2010 Feb;19(1):73-80.
162. Imburgia M, Del Fabbro M. Long-Term Retrospective Clinical and Radiographic Follow-up of 205 Brånemark System Mk III TiUnite Implants Submitted to Either Immediate or Delayed Loading. Implant Dent. 2015 Oct;24(5):533-40.
163. Ivanoff CJ, Gröndahl K, Sennerby L, Bergström C, Lekholm U. Influence of variations in implant diameters: a 3- to 5-year retrospective clinical report. Int J Oral Maxillofac Implants. 1999 Mar-Apr;14(2):173-80.
164. Jang HW, Kang JK, Lee K, Lee YS, Park PK. A retrospective study on related factors affecting the survival rate of dental implants. J Adv Prosthodont. 2011 Dec;3(4):204-15.
165. Jemt T, Johansson J. Implant treatment in the edentulous maxillae: a 15-year follow-up study on 76 consecutive patients provided with fixed prostheses. Clin Implant Dent Relat Res. 2006;8(2):61-9.
166. Jemt T, Laney WR, Harris D, Henry PJ, Krogh PH Jr, Polizzi G, Zarb GA, Herrmann I. Osseointegrated implants for single tooth replacement: a 1-year report from a multicenter prospective study. Int J Oral Maxillofac Implants. 1991 Spring;6(1):29-36.
167. Jonker BP, Wolvius EB, van der Tas JT, Pijpe J. The effect of resorbable membranes on one-stage ridge augmentation in anterior single-tooth replacement: A randomized, controlled clinical trial. Clin Oral Implants Res. 2018 Feb;29(2):235-247.
168. Kahnberg KE, Vannas-Löfqvist L. Sinus lift procedure using a 2-stage surgical technique: I. Clinical and radiographic report up to 5 years. Int J Oral Maxillofac Implants. 2008 Sep-Oct;23(5):876-84.
169. Kaneda K, Kondo Y, Masaki C, Mukaibo T, Tsuka S, Tamura A, Aonuma F, Shinmyouzu K, Iwasaki M, Ansai T, Hosokawa R. Ten-year survival of immediate-loading implants in fully edentulous mandibles in the Japanese population: a multilevel analysis. J Prosthodont Res. 2019 Jan;63(1):35-39.
170. Karlsson U, Gotfredsen K, Olsson C. A 2-year report on maxillary and mandibular fixed partial dentures supported by Astra Tech dental implants. A comparison of 2 implants with different surface textures. Clin Oral Implants Res. 1998 Aug;9(4):235-42.
171. Keller EE, Tolman DE, Eckert SE. Maxillary antral-nasal inlay autogenous bone graft reconstruction of compromised maxilla: a 12-year retrospective study. Int J Oral Maxillofac Implants. 1999 Sep-Oct;14(5):707-21.
172. Kennedy KS, Jones EM, Kim DG, McGlumphy EA, Clelland NL. A prospective clinical study to evaluate early success of short implants. Int J Oral Maxillofac Implants. 2013 Jan-Feb;28(1):170-7.
173. Khayat PG, Hallage PG, Toledo RA. An investigation of 131 consecutively placed wide screw-vent implants. Int J Oral Maxillofac Implants. 2001 Nov-Dec;16(6):827-32.
174. Khouly I, Pardiñas López S, Aliaga I, Froum SJ. Long-Term Implant Survival After 100 Maxillary Sinus Augmentations Using Plasma Rich in Growth Factors. Implant Dent. 2017 Apr;26(2):199-208.
175. Kim S, Jung UW, Cho KS, Lee JS. Retrospective radiographic observational study of 1692 Straumann tissue-level dental implants over 10 years: I. Implant survival and loss pattern. Clin Implant Dent Relat Res. 2018 Oct;20(5):860-866.
176. Kim SY, Dodson TB, Do DT, Wadhwa G, Chuang SK. Factors Associated With Crestal Bone Loss Following Dental Implant Placement in a Longitudinal Follow-up Study. J Oral Implantol. 2015 Oct;41(5):579-85.
177. Kinsel RP, Liss M. Retrospective analysis of 56 edentulous dental arches restored with 344 single-stage implants using an immediate loading fixed provisional protocol: statistical predictors of implant failure. Int J Oral Maxillofac Implants. 2007 Sep-Oct;22(5):823-30.
178. Kokovic V, Jung R, Feloutzis A, Todorovic VS, Jurisic M, Hämmerle CH. Immediate vs. early loading of SLA implants in the posterior mandible: 5-year results of randomized controlled clinical trial. Clin Oral Implants Res. 2014 Feb;25(2):e114-9.
179. Koo KT, Wikesjö UM, Park JY, Kim TI, Seol YJ, Ku Y, Rhyu IC, Chung CP, Lee YM. Evaluation of single-tooth implants in the second molar region: a 5-year life-table analysis of a retrospective study. J Periodontol. 2010 Sep;81(9):1242-9.
180. Kovács AF. Clinical analysis of implant losses in oral tumor and defect patients. Clin Oral Implants Res. 2000 Oct;11(5):494-504.
181. Lago L, da Silva L, Martinez-Silva I, Rilo B. Crestal Bone Level Around Tissue-Level Implants Restored with Platform Matching and Bone-Level Implants Restored with Platform Switching: A 5-Year Randomized Controlled Trial. Int J Oral Maxillofac Implants. 2018 Mar/Apr;33(2):448-456.

182. Lai HC, Zhang ZY, Wang F, Zhuang LF, Liu X. Resonance frequency analysis of stability on ITI implants with osteotome sinus floor elevation technique without grafting: a 5-month prospective study. Clin Oral Implants Res. 2008 May;19(5):469-75.
183. Lai HC, Zhang ZY, Zhuang LF, Wang F, Liu X, Pu YP. Early loading of ITI implants supporting maxillary fixed full-arch prostheses. Clin Oral Implants Res. 2008 Nov;19(11):1129-34.
184. Lai HC, Zhuang LF, Lv XF, Zhang ZY, Zhang YX, Zhang ZY. Osteotome sinus floor elevation with or without grafting: a preliminary clinical trial. Clin Oral Implants Res. 2010 May;21(5):520-6.
185. Lee CT, Tran D, Jeng MD, Shen YT. Survival rates of hybrid rough surface implants and their alveolar bone level alterations. J Periodontol. 2018 Dec;89(12):1390-1399.
186. Lee DW, Lee DW, Park KH, Moon IS. The effects of off-axial loading on periimplant marginal bone loss in a single implant. J Prosthet Dent. 2014 Sep;112(3):501-7.
187. Lee JS, Kim HM, Kim CS, Choi SH, Chai JK, Jung UW. Long-term retrospective study of narrow implants for fixed dental prostheses. Clin Oral Implants Res. 2013 Aug;24(8):847-52.
188. Lekholm U, Gunne J, Henry P, Higuchi K, Lindén U, Bergström C, van Steenberghe D. Survival of the Brånemark implant in partially edentulous jaws: a 10-year prospective multicenter study. Int J Oral Maxillofac Implants. 1999 Sep-Oct;14(5):639-45.
189. Levine RA, Clem DS 3rd, Wilson TG Jr, Higginbottom F, Saunders SL. A multicenter retrospective analysis of the ITI implant system used for single-tooth replacements: preliminary results at 6 or more months of loading. Int J Oral Maxillofac Implants. 1997 Mar-Apr;12(2):237-42.
190. Levine RA, Ganeles J, Jaffin RA, Clem DS 3rd, Beagle JR, Keller GW. Multicenter retrospective analysis of wide-neck dental implants for single molar replacement. Int J Oral Maxillofac Implants. 2007 Sep-Oct;22(5):736-42.
191. Lin G, Ye S, Liu F, He F. A retrospective study of 30,959 implants: Risk factors associated with early and late implant loss. J Clin Periodontol. 2018 Jun;45(6):733-743.
192. Lindgren C, Mordenfeld A, Hallman M. A prospective 1-year clinical and radiographic study of implants placed after maxillary sinus floor augmentation with synthetic biphasic calcium phosphate or deproteinized bovine bone. Clin Implant Dent Relat Res. 2012 Mar;14(1):41-50.
193. Lini F, Poli PP, Beretta M, Cortinovis I, Maiorana C. Long-term retrospective observational cohort study on the survival rate of stepped screw titanium implants followed up to 20 years. Int J Oral Maxillofac Implants. 2019 July/August;34(4):999–1006.
194. Lobato RPB, Kinalski MA, Martins TM, Agostini BA, Bergoli CD, Dos Santos MBF. Influence of low-level laser therapy on implant stability in implants placed in fresh extraction sockets: A randomized clinical trial. Clin Implant Dent Relat Res. 2020 Jun;22(3):261-269.
195. Lopez-Cedrun JL. Implant rehabilitation of the edentulous posterior atrophic mandible: the sandwich osteotomy revisited. Int J Oral Maxillofac Implants. 2011 Jan-Feb;26(1):195-202.
196. Lops D, Romeo E, Chiapasco M, Procopio RM, Oteri G. Behaviour of soft tissues healing around single bone-level-implants placed immediately after tooth extraction A 1 year prospective cohort study. Clin Oral Implants Res. 2013 Nov;24(11):1206-13.
197. Ma W, Duan Y, Sun B, Liu Y, Xie C, Li D. A 5-year retrospective study on postsurgical periimplant infection during initial bone healing period: clinical characteristics, management, and prognosis. Implant Dent. 2013 Feb;22(1):20-5.
198. Makkonen TA, Holmberg S, Niemi L, Olsson C, Tammisalo T, Peltola J. A 5-year prospective clinical study of Astra Tech dental implants supporting fixed bridges or overdentures in the edentulous mandible. Clin Oral Implants Res. 1997 Dec;8(6):469-75.
199. Malchiodi L, Caricasulo R, Cucchi A, Vinci R, Agliardi E, Gherlone E. Evaluation of Ultrashort and Longer Implants with Microrough Surfaces: Results of a 24- to 36-Month Prospective Study. Int J Oral Maxillofac Implants. 2017 Jan/Feb;32(1):171-179.
200. Malchiodi L, Ghensi P, Cucchi A, Corrocher G. A comparative retrospective study of immediately loaded implants in postextraction sites versus healed sites: results after 6 to 7 years in the maxilla. Int J Oral Maxillofac Implants. 2011 Mar-Apr;26(2):373-84.
201. Malmstrom H, Gupta B, Ghanem A, Cacciato R, Ren Y, Romanos GE. Success rate of short dental implants supporting single crowns and fixed bridges. Clin Oral Implants Res. 2016 Sep;27(9):1093-8.
202. Maló P, de Araújo Nobre M, Lopes A, Ferro A, Gravito I. Single-Tooth Rehabilitations Supported by Dental Implants Used in an Immediate-Provisionalization Protocol: Report on Long-Term Outcome with Retrospective Follow-Up. Clin Implant Dent Relat Res. 2015 Oct;17 Suppl 2:e511-9.
203. Maló P, de Araújo Nobre M, Lopes A, Queridinha B, Ferro A, Gravito I. Axial Implants in Immediate Function for Partial Rehabilitation in the Maxilla and Mandible: A Retrospective Clinical Study Evaluating the Long-Term Outcome (Up to 10 Years). Implant Dent. 2015 Oct;24(5):557-64.

204. Mangano F, Macchi A, Caprioglio A, Sammons RL, Piattelli A, Mangano C. Survival and complication rates of fixed restorations supported by locking-taper implants: a prospective study with 1 to 10 years of follow-up. J Prosthodont. 2014 Aug;23(6):434-44.
205. Mangano F, Shibli JA, Sammons RL, Veronesi G, Piattelli A, Mangano C. Clinical outcome of narrow-diameter (3.3-mm) locking-taper implants: a prospective study with 1 to 10 years of follow-up. Int J Oral Maxillofac Implants. 2014 Mar-Apr;29(2):448-55.
206. Manni LL, Lecloux G, Rompen E, Aouini W, Shapira L, Lambert F. Clinical and radiographic assessment of circular versus triangular cross-section neck Implants in the posterior maxilla: A 1-year randomized controlled trial. Clin Oral Implants Res. 2020 Sep;31(9):814-824.
207. Mattsson T, Köndell PA, Gynther GW, Fredholm U, Bolin A. Implant treatment without bone grafting in severely resorbed edentulous maxillae. J Oral Maxillofac Surg. 1999 Mar;57(3):281-7.
208. McGlumphy EA, Peterson LJ, Larsen PE, Jeffcoat MK. Prospective study of 429 hydroxyapatite-coated cylindric omniloc implants placed in 121 patients. Int J Oral Maxillofac Implants. 2003 Jan-Feb;18(1):82-92.
209. Mei DM, Zhao B, Xu H, Wang Y. Radiographic and clinical outcomes of rooted, platform-switched, microthreaded implants with a sandblasted, large-grid, and acid-etched surface: A 5-year prospective study. Clin Implant Dent Relat Res. 2017 Dec;19(6):1074-1081.
210. Meloni SM, Baldoni E, Duvina M, Pisano M, De Riu G, Tallarico M. Immediate non-occlusal versus delayed loading of mandibular first molars. Five-year results from a randomised controlled trial. Eur J Oral Implantol. 2018;11(4):409-418.
211. Mendonça JA, Francischone CE, Senna PM, Matos de Oliveira AE, Sotto-Maior BS. A retrospective evaluation of the survival rates of splinted and non-splinted short dental implants in posterior partially edentulous jaws. J Periodontol. 2014 Jun;85(6):787-94.
212. Mendonça JA, Senna PM, Francischone CE, Francischone Junior CE, de Souza Picorelli Assis NM, Sotto-Maior BS. Retrospective Evaluation of the Influence of the Collar Surface Topography on Peri-implant Bone Preservation. Int J Oral Maxillofac Implants. 2017 Jul/Aug;32(4):858-863.
213. Mendonça JA, Senna PM, Francischone CE, Francischone Junior CE, Sotto-Maior BS. Influence of the Diameter of Dental Implants Replacing Single Molars: 3- to 6-Year Follow-Up. Int J Oral Maxillofac Implants. 2017 Sep/Oct;32(5):1111-1115.
214. Mericske-Stern R, Oetterli M, Kiener P, Mericske E. A follow-up study of maxillary implants supporting an overdenture: clinical and radiographic results. Int J Oral Maxillofac Implants. 2002 Sep-Oct;17(5):678-86.
215. Merli M, Merli A, Bernardelli F, Lombardini F, Esposito M. Immediate versus early non-occlusal loading of dental implants placed flapless in partially edentulous patients. One-year results from a randomised controlled trial. Eur J Oral Implantol. 2008 Autumn;1(3):207-20.
216. Mertens C, Steveling HG, Stucke K, Pretzl B, Meyer-Bäumer A. Fixed implant-retained rehabilitation of the edentulous maxilla: 11-year results of a prospective study. Clin Implant Dent Relat Res. 2012 Dec;14(6):816-27.
217. Mertens C, Steveling HG. Early and immediate loading of titanium implants with fluoride-modified surfaces: results of 5-year prospective study. Clin Oral Implants Res. 2011 Dec;22(12):1354-60.
218. Mijiritsky E, Mazor Z, Lorean A, Levin L. Implant diameter and length influence on survival: interim results during the first 2 years of function of implants by a single manufacturer. Implant Dent. 2013 Aug;22(4):394-8.
219. Mo A, Hjortsjö C, Olsen-Bergem H, Jokstad A. Maxillary 3-implant removable prostheses without palatal coverage on Locator abutments - a case series. Clin Oral Implants Res. 2016 Oct;27(10):1193-1199.
220. Mongardini C, Zeza B, Pelagalli P, Blasone R, Scilla M, Berardini M. Radiographic bone level around particular laser-treated dental implants: 1 to 6 years multicenter retrospective study. Int J Implant Dent. 2020 Jul 28;6(1):29.
221. Mozzati M, Gallesio G, Del Fabbro M. Long-Term (9-12 Years) Outcomes of Titanium Implants With an Oxidized Surface: A Retrospective Investigation on 209 Implants. J Oral Implantol. 2015 Aug;41(4):437-43.
222. Mumcu E, Dereci Ö. Assessment of the Effect of Clinical Independent Risk Factors on Marginal Bone Loss in 2-Implant-Supported Locator-Retained Mandibular Overdentures. J Oral Implantol. 2019 Jun;45(3):207-212.
223. Munakata M, Tachikawa N, Yamaguchi Y, Sanda M, Kasugai S. The Maxillary Sinus Floor Elevation Using a Poly-L-Lactic Acid Device to Create Space Without Bone Graft: Case Series Study of Five Patients. J Oral Implantol. 2016 Jun;42(3):278-84.
224. Nedir R, Bischof M, Briaux JM, Beyer S, Szmukler-Moncler S, Bernard JP. A 7-year life table analysis from a prospective study on ITI implants with special emphasis on the use of short implants. Results from a private practice. Clin Oral Implants Res. 2004 Apr;15(2):150-7.
225. Nevins M, Langer B. The successful application of osseointegrated implants to the posterior jaw: a long-term retrospective study. Int J Oral Maxillofac Implants. 1993;8(4):428-32.
226. Niedermaier R, Stelzle F, Riemann M, Bolz W, Schuh P, Wachtel H. Implant-Supported Immediately Loaded Fixed Full-Arch Dentures: Evaluation of Implant Survival Rates in a Case Cohort of up to 7 Years. Clin Implant Dent Relat Res. 2017 Feb;19(1):4-19.
227. Niimi A, Ueda M, Keller EE, Worthington P. Experience with osseointegrated implants placed in irradiated tissues in Japan and the United States. Int J Oral Maxillofac Implants. 1998 May-Jun;13(3):407-11.

228. Nogueira TE, Aguiar FMO, de Barcelos BA, Leles CR. A 2-year prospective study of single-implant mandibular overdentures: Patient-reported outcomes and prosthodontic events. Clin Oral Implants Res. 2018 Jun;29(6):541-550.
229. Norton MR. The Influence of Low Insertion Torque on Primary Stability, Implant Survival, and Maintenance of Marginal Bone Levels: A Closed-Cohort Prospective Study. Int J Oral Maxillofac Implants. 2017 Jul/Aug;32(4):849-857.
230. Olate S, Lyrio MC, de Moraes M, Mazzonetto R, Moreira RW. Influence of diameter and length of implant on early dental implant failure. J Oral Maxillofac Surg. 2010 Feb;68(2):414-9.
231. Olson JW, Shernoff AF, Tarlow JL, Colwell JA, Scheetz JP, Bingham SF. Dental endosseous implant assessments in a type 2 diabetic population: a prospective study. Int J Oral Maxillofac Implants. 2000 Nov-Dec;15(6):811-8.
232. Olsson M, Friberg B, Nilson H, Kultje C. MkII--a modified self-tapping Brånemark implant: 3-year results of a controlled prospective pilot study. Int J Oral Maxillofac Implants. 1995 Jan-Feb;10(1):15-21.
233. Olsson M, Urde G, Andersen JB, Sennerby L. Early loading of maxillary fixed cross-arch dental prostheses supported by six or eight oxidized titanium implants: results after 1 year of loading, case series. Clin Implant Dent Relat Res. 2003;5 Suppl 1:81-7.
234. Ormianer Z, Palti A. Long-term clinical evaluation of tapered multi-threaded implants: results and influences of potential risk factors. J Oral Implantol. 2006;32(6):300-7.
235. Ormianer Z, Palti A. Retrospective clinical evaluation of tapered screw-vent implants: results after up to eight years of clinical function. J Oral Implantol. 2008;34(3):150-60.
236. Örtorp A, Jemt T. Laser-welded titanium frameworks supported by implants in the partially edentulous mandible: a 10-year comparative follow-up study. Clin Implant Dent Relat Res. 2008 Sep;10(3):128-39.
237. Östman PO, Hellman M, Sennerby L. Immediate occlusal loading of implants in the partially edentate mandible: a prospective 1-year radiographic and 4-year clinical study. Int J Oral Maxillofac Implants. 2008 Mar-Apr;23(2):315-22.
238. Östman PO, Hellman M, Sennerby L. Ten years later. Results from a prospective single-centre clinical study on 121 oxidized (TiUnite™) Brånemark implants in 46 patients. Clin Implant Dent Relat Res. 2012 Dec;14(6):852-60.
239. Palmqvist S, Sondell K, Swartz B. Implant-supported maxillary overdentures: outcome in planned and emergency cases. Int J Oral Maxillofac Implants. 1994 Mar-Apr;9(2):184-90.
240. Payne AG, Tawse-Smith A, Wismeijer D, De Silva RK, Ma S. Multicentre prospective evaluation of implant-assisted mandibular removable partial dentures: surgical and prosthodontic outcomes. Clin Oral Implants Res. 2017 Jan;28(1):116-125.
241. Peñarrocha-Diago M, Carrillo-Garcîa C, Boronat-Lopez A, García-Mira B. Comparative study of wide-diameter implants placed after dental extraction and implants positioned in mature bone for molar replacement. Int J Oral Maxillofac Implants. 2008 May-Jun;23(3):497-501.
242. Peñarrocha-Oltra D, Aloy-Prósper A, Cervera-Ballester J, Peñarrocha-Diago M, Canullo L, Peñarrocha-Diago M. Implant treatment in atrophic posterior mandibles: vertical regeneration with block bone grafts versus implants with 5.5-mm intrabony length. Int J Oral Maxillofac Implants. 2014 May-Jun;29(3):659-66.
243. Peñarrocha-Oltra D, Covani U, Peñarrocha M, Peñarrocha-Diago M. Immediate versus conventional loading with fixed full-arch prostheses in mandibles with failing dentition: a prospective controlled study. Int J Oral Maxillofac Implants. 2015 Mar-Apr;30(2):427-34.
244. Peñarrocha-Oltra D, Demarchi CL, Maestre-Ferrín L, Peñarrocha-Diago M, Peñarrocha-Diago M. Comparison of immediate and delayed implants in the maxillary molar region: a retrospective study of 123 implants. Int J Oral Maxillofac Implants. 2012 May-Jun;27(3):604-10.
245. Pettersson P, Sennerby L. A 5-year retrospective study on Replace Select Tapered dental implants. Clin Implant Dent Relat Res. 2015 Apr;17(2):286-95.
246. Piano S, Romeo E, Sbricoli L, Pisoni G, Cea N, Lops D. Simplified procedure for the immediate loading of a complete fixed prosthesis supported by four implants in the maxillary jaw: a 2-year prospective study. Clin Oral Implants Res. 2016 Dec;27(12):e154-e160.
247. Pico A, Martín-Lancharro P, Caneiro L, Nóvoa L, Batalla P, Blanco J. Influence of abutment height and implant depth position on interproximal peri-implant bone in sites with thin mucosa: A 1-year randomized clinical trial. Clin Oral Implants Res. 2019 Jul;30(7):595-602.
248. Pieri F, Caselli E, Forlivesi C, Corinaldesi G. Rehabilitation of the Atrophic Posterior Maxilla Using Splinted Short Implants or Sinus Augmentation with Standard-Length Implants: A Retrospective Cohort Study. Int J Oral Maxillofac Implants. 2016 Sep-Oct;31(5):1179-88.
249. Pinholt EM. Brånemark and ITI dental implants in the human bone-grafted maxilla: a comparative evaluation. Clin Oral Implants Res. 2003 Oct;14(5):584-92.
250. Pistilli R, Felice P, Cannizzaro G, Piatelli M, Corvino V, Barausse C, Buti J, Soardi E, Esposito M. Posterior atrophic jaws rehabilitated with prostheses supported by 6 mm long 4 mm wide implants or by longer implants in augmented bone. One-year post-loading results from a pilot randomised controlled trial. Eur J Oral Implantol. 2013 Winter;6(4):359-72.

251. Pistilli R, Felice P, Piattelli M, Gessaroli M, Soardi E, Barausse C, Buti J, Corvino V. Posterior atrophic jaws rehabilitated with prostheses supported by 5 x 5 mm implants with a novel nanostructured calcium-incorporated titanium surface or by longer implants in augmented bone. One-year results from a randomised controlled trial. Eur J Oral Implantol. 2013 Winter;6(4):343-57.
252. Pjetursson BE, Rast C, Brägger U, Schmidlin K, Zwahlen M, Lang NP. Maxillary sinus floor elevation using the (transalveolar) osteotome technique with or without grafting material. Part I: Implant survival and patients' perception. Clin Oral Implants Res. 2009 Jul;20(7):667-76.
253. Polizzi G, Rangert B, Lekholm U, Gualini F, Lindström H. Brånemark System Wide Platform implants for single molar replacement: clinical evaluation of prospective and retrospective materials. Clin Implant Dent Relat Res. 2000;2(2):61-9.
254. Pozzi A, Mura P. Clinical and radiologic experience with moderately rough oxidized titanium implants: up to 10 years of retrospective follow-up. Int J Oral Maxillofac Implants. 2014 Jan-Feb;29(1):152-61.
255. Pozzi A, Tallarico M, Marchetti M, Scarfò B, Esposito M. Computer-guided versus free-hand placement of immediately loaded dental implants: 1-year post-loading results of a multicentre randomised controlled trial. Eur J Oral Implantol. 2014 Autumn;7(3):229-42.
256. Priest G. Single-tooth implants and their role in preserving remaining teeth: a 10-year survival study. Int J Oral Maxillofac Implants. 1999 Mar-Apr;14(2):181-8.
257. Prosper L, Crespi R, Valenti E, Capparé P, Gherlone E. Five-year follow-up of wide-diameter implants placed in fresh molar extraction sockets in the mandible: immediate versus delayed loading. Int J Oral Maxillofac Implants. 2010 May-Jun;25(3):607-12.
258. Qian SJ, Mo JJ, Si MS, Qiao SC, Shi JY, Lai HC. Long-term outcomes of osteotome sinus floor elevation with or without bone grafting: The 10-year results of a randomized controlled trial. J Clin Periodontol. 2020 Aug;47(8):1016-1025.
259. Queiroz TP, Aguiar SC, Margonar R, de Souza Faloni AP, Gruber R, Luvizuto ER. Clinical study on survival rate of short implants placed in the posterior mandibular region: resonance frequency analysis. Clin Oral Implants Res. 2015 Sep;26(9):1036-42.
260. Queridinha BM, Almeida RF, Felino A, de Araújo Nobre M, Maló P. Partial Rehabilitation with Distally Tilted and Straight Implants in the Posterior Maxilla with Immediate Loading Protocol: A Retrospective Cohort Study with 5-Year Follow-up. Int J Oral Maxillofac Implants. 2016 Jul-Aug;31(4):891-9.
261. Rammelsberg P, Bernhart G, Lorenzo Bermejo J, Schmitter M, Schwarz S. Prognosis of implants and abutment teeth under combined tooth-implant-supported and solely implant-supported double-crown-retained removable dental prostheses. Clin Oral Implants Res. 2014 Jul;25(7):813-8.
262. Renouard F, Arnoux JP, Sarment DP. Five-mm-diameter implants without a smooth surface collar: report on 98 consecutive placements. Int J Oral Maxillofac Implants. 1999 Jan-Feb;14(1):101-7.
263. Riben C, Thor A. Follow-Up of the Sinus Membrane Elevation Technique for Maxillary Sinus Implants without the Use of Graft Material. Clin Implant Dent Relat Res. 2016 Oct;18(5):895-905.
264. Rocci A, Martignoni M, Gottlow J. Immediate loading in the maxilla using flapless surgery, implants placed in predetermined positions, and prefabricated provisional restorations: a retrospective 3-year clinical study. Clin Implant Dent Relat Res. 2003;5 Suppl 1:29-36.
265. Rocci A, Martignoni M, Gottlow J. Immediate loading of Brånemark System TiUnite and machined-surface implants in the posterior mandible: a randomized open-ended clinical trial. Clin Implant Dent Relat Res. 2003;5 Suppl 1:57-63.
266. Roccuzzo M, Bunino M, Prioglio F, Bianchi SD. Early loading of sandblasted and acid-etched (SLA) implants: a prospective split-mouth comparative study. Clin Oral Implants Res. 2001 Dec;12(6):572-8.
267. Roccuzzo M, Wilson T. A prospective study evaluating a protocol for 6 weeks' loading of SLA implants in the posterior maxilla: one year results. Clin Oral Implants Res. 2002 Oct;13(5):502-7.
268. Romanos GE, Gaertner K, Nentwig GH. Long-term evaluation of immediately loaded implants in the edentulous mandible using fixed bridges and platform shifting. Clin Implant Dent Relat Res. 2014 Aug;16(4):601-8.
269. Romanos GE, May S, May D. Immediate loading of tooth-implant-supported telescopic mandibular prostheses. Int J Oral Maxillofac Implants. 2012 Nov-Dec;27(6):1534-40.
270. Romanos GE, Nentwig GH. Single molar replacement with a progressive thread design implant system: a retrospective clinical report. Int J Oral Maxillofac Implants. 2000 Nov-Dec;15(6):831-6.
271. Romeo E, Lops D, Margutti E, Ghisolfi M, Chiapasco M, Vogel G. Long-term survival and success of oral implants in the treatment of full and partial arches: a 7-year prospective study with the ITI dental implant system. Int J Oral Maxillofac Implants. 2004 Mar-Apr;19(2):247-59.
272. Romeo E, Tomasi C, Finini I, Casentini P, Lops D. Implant-supported fixed cantilever prosthesis in partially edentulous jaws: a cohort prospective study. Clin Oral Implants Res. 2009 Nov;20(11):1278-85.
273. Roos J, Sennerby L, Lekholm U, Jemt T, Gröndahl K, Albrektsson T. A qualitative and quantitative method for evaluating implant success: a 5-year retrospective analysis of the Brånemark implant. Int J Oral Maxillofac Implants. 1997 Jul-Aug;12(4):504-14.

274. Rosenberg ES, Cho SC, Elian N, Jalbout ZN, Froum S, Evian CI. A comparison of characteristics of implant failure and survival in periodontally compromised and periodontally healthy patients: a clinical report. Int J Oral Maxillofac Implants. 2004 Nov-Dec;19(6):873-9.
275. Rossi F, Botticelli D, Cesaretti G, De Santis E, Storelli S, Lang NP. Use of short implants (6 mm) in a single-tooth replacement: a 5-year follow-up prospective randomized controlled multicenter clinical study. Clin Oral Implants Res. 2016 Apr;27(4):458-64.
276. Salvi GE, Gallini G, Lang NP. Early loading (2 or 6 weeks) of sandblasted and acid-etched (SLA) ITI implants in the posterior mandible. A 1-year randomized controlled clinical trial. Clin Oral Implants Res. 2004 Apr;15(2):142-9.
277. Santis D, Cucchi A, Rigoni G, Longhi C, Nocini PF. Relationship Between Primary Stability and Crestal Bone Loss of Implants Placed with High Insertion Torque: A 3-Year Prospective Study. Int J Oral Maxillofac Implants. 2016 Sep-Oct;31(5):1126-34.
278. Sbordone C, Toti P, Martuscelli R, Guidetti F, Sbordone L, Ramaglia L. A 5-Year Implant Follow-Up in Maxillary and Mandibular Horizontal Osseous Onlay Grafts and Native Bone. J Oral Implantol. 2015 Oct;41(5):570-8.
279. Schincaglia GP, Marzola R, Giovanni GF, Chiara CS, Scotti R. Replacement of mandibular molars with single-unit restorations supported by wide-body implants: immediate versus delayed loading. A randomized controlled study. Int J Oral Maxillofac Implants. 2008 May-Jun;23(3):474-80.
280. Schincaglia GP, Marzola R, Scapoli C, Scotti R. Immediate loading of dental implants supporting fixed partial dentures in the posterior mandible: a randomized controlled split-mouth study--machined versus titanium oxide implant surface. Int J Oral Maxillofac Implants. 2007 Jan-Feb;22(1):35-46.
281. Schnitman PA, Hwang JW. To immediately load, expose, or submerge in partial edentulism: a study of primary stability and treatment outcome. Int J Oral Maxillofac Implants. 2011 Jul-Aug;26(4):850-9.
282. Schnitman PA, Wöhrle PS, Rubenstein JE, DaSilva JD, Wang NH. Ten-year results for Brånemark implants immediately loaded with fixed prostheses at implant placement. Int J Oral Maxillofac Implants. 1997 Jul-Aug;12(4):495-503
283. Scurria MS, Morgan ZV 4th, Guckes AD, Li S, Koch G. Prognostic variables associated with implant failure: a retrospective effectiveness study. Int J Oral Maxillofac Implants. 1998 May-Jun;13(3):400-6.
284. Şener-Yamaner ID, Yamaner G, Sertgöz A, Çanakçi CF, Özcan M. Marginal Bone Loss Around Early-Loaded SLA and SLActive Implants: Radiological Follow-Up Evaluation Up to 6.5 Years. Implant Dent. 2017 Aug;26(4):592-599.
285. Sennerby L, Andersson P, Verrocchi D, Viinamäki R. One-year outcomes of Neoss bimodal implants. A prospective clinical, radiographic, and RFA study. Clin Implant Dent Relat Res. 2012 Jun;14(3):313-20.
286. Sethi A, Kaus T. Maxillary ridge expansion with simultaneous implant placement: 5-year results of an ongoing clinical study. Int J Oral Maxillofac Implants. 2000 Jul-Aug;15(4):491-9.
287. Shi JY, Li Y, Qiao SC, Gu YX, Xiong YY, Lai HC. Short versus longer implants with osteotome sinus floor elevation for moderately atrophic posterior maxillae: A 1-year randomized clinical trial. J Clin Periodontol. 2019 Aug;46(8):855-862.
288. Shi JY, Zhang XM, Qiao SC, Qian SJ, Mo JJ, Lai HC. Hardware complications and failure of three-unit zirconia-based and porcelain-fused-metal implant-supported fixed dental prostheses: a retrospective cohort study with up to 8 years. Clin Oral Implants Res. 2017 May;28(5):571-575.
289. Shigehara S, Ohba S, Nakashima K, Takanashi Y, Asahina I. Immediate Loading of Dental Implants Inserted in Edentulous Maxillas and Mandibles: 5-Year Results of a Clinical Study. J Oral Implantol. 2015 Dec;41(6):701-5.
290. Si MS, Shou YW, Shi YT, Yang GL, Wang HM, He FM. Long-term outcomes of osteotome sinus floor elevation without bone grafts: a clinical retrospective study of 4-9 years. Clin Oral Implants Res. 2016 Nov;27(11):1392-1400.
291. Smedberg JI, Lothigius E, Nilner K, De Buck V. A new design for a hybrid prosthesis supported by osseointegrated implants: 2. Preliminary clinical aspects. Int J Oral Maxillofac Implants. 1991 Summer;6(2):154-9.
292. Sohn DS, Kim WS, Lee WH, Jung HS, Shin IH. A retrospective study of sintered porous-surfaced dental implants in restoring the edentulous posterior mandible: up to 9 years of functioning. Implant Dent. 2010 Oct;19(5):409-18.
293. Souza AB, Sukekava F, Tolentino L, César-Neto JB, Garcez-Filho J, Araújo MG. Narrow- and regular-diameter implants in the posterior region of the jaws to support single crowns: A 3-year split-mouth randomized clinical trial. Clin Oral Implants Res. 2018 Jan;29(1):100-107.
294. Souza CSV, Ortega-Lopes R, Barreno AC, de Moraes M, Albergaria-Barbosa JR, Nóia CF. Analysis of the Survival of Dental Implants Installed in Reconstructed Maxilla With Autogenous Iliac Crest Graft: 7- to 9-Year Follow-Up. J Oral Implantol. 2019 Dec;45(6):427-436.
295. Stanford CM, Barwacz C, Raes S, De Bruyn H, Cecchinato D, Bittner N, Brandt J. Multicenter Clinical Randomized Controlled Trial Evaluation of an Implant System Designed for Enhanced Primary Stability. Int J Oral Maxillofac Implants. 2016 Jul-Aug;31(4):906-15.
296. Steveling H, Roos J, Rasmusson L. Maxillary implants loaded at 3 months after insertion: results with Astra Tech implants after up to 5 years. Clin Implant Dent Relat Res. 2001;3(3):120-4.
297. Storelli S, Abbà A, Scanferla M, Botticelli D, Romeo E. 6 mm vs 10 mm-long implants in the rehabilitation of posterior jaws: A 10-year follow-up of a randomised controlled trial. Eur J Oral Implantol. 2018;11(3):283-292.

298. Tallarico M, Esposito M, Xhanari E, Caneva M, Meloni SM. Computer-guided vs freehand placement of immediately loaded dental implants: 5-year postloading results of a randomised controlled trial. Eur J Oral Implantol. 2018;11(2):203-213.
299. Tallarico M, Xhanari E, Pisano M, Gatti F, Meloni SM. Molar replacement with 7 mm-wide diameter implants: to place the implant immediately or to wait 4 months after socket preservation? 1 year after loading results from a randomised controlled trial. Eur J Oral Implantol. 2017;10(2):169-178.
300. Tang YL, Yuan J, Song YL, Ma W, Chao X, Li DH. Ridge expansion alone or in combination with guided bone regeneration to facilitate implant placement in narrow alveolar ridges: a retrospective study. Clin Oral Implants Res. 2015 Feb;26(2):204-11.
301. Taschieri S, Corbella S, Del Fabbro M. Mini-invasive osteotome sinus floor elevation in partially edentulous atrophic maxilla using reduced length dental implants: interim results of a prospective study. Clin Implant Dent Relat Res. 2014 Apr;16(2):185-93.
302. Taschieri S, Lolato A, Testori T, Francetti L, Del Fabbro M. Short dental implants as compared to maxillary sinus augmentation procedure for the rehabilitation of edentulous posterior maxilla: Three-year results of a randomized clinical study. Clin Implant Dent Relat Res. 2018 Feb;20(1):9-20.
303. Tawil G, Mawla M, Gottlow J. Clinical and radiographic evaluation of the 5-mm diameter regular-platform Brånemark fixture: 2- to 5-year follow-up. Clin Implant Dent Relat Res. 2002;4(1):16-26.
304. Tawil G, Mawla M. Sinus floor elevation using a bovine bone mineral (Bio-Oss) with or without the concomitant use of a bilayered collagen barrier (Bio-Gide): a clinical report of immediate and delayed implant placement. Int J Oral Maxillofac Implants. 2001 Sep-Oct;16(5):713-21.
305. Tawil G, Younan R. Clinical evaluation of short, machined-surface implants followed for 12 to 92 months. Int J Oral Maxillofac Implants. 2003 Nov-Dec;18(6):894-901.
306. Temmerman A, Keestra JA, Coucke W, Teughels W, Quirynen M. The outcome of oral implants placed in bone with limited bucco-oral dimensions: a 3-year follow-up study. J Clin Periodontol. 2015 Mar;42(3):311-8.
307. Testori T, Del Fabbro M, Feldman S, Vincenzi G, Sullivan D, Rossi R Jr, Anitua E, Bianchi F, Francetti L, Weinstein RL. A multicenter prospective evaluation of 2-months loaded Osseotite implants placed in the posterior jaws: 3-year follow-up results. Clin Oral Implants Res. 2002 Apr;13(2):154-61.
308. Testori T, Del Fabbro M, Galli F, Francetti L, Taschieri S, Weinstein R. Immediate occlusal loading the same day or the after implant placement: comparison of 2 different time frames in total edentulous lower jaws. J Oral Implantol. 2004;30(5):307-13.
309. Testori T, Galli F, Fumagalli L, Capelli M, Zuffetti F, Deflorian M, Parenti A, Del Fabbro M. Assessment of Long-Term Survival of Immediately Loaded Tilted Implants Supporting a Maxillary Full-Arch Fixed Prosthesis. Int J Oral Maxillofac Implants. 2017 Jul/Aug;32(4):904-911
310. Testori T, Wiseman L, Woolfe S, Porter SS. A prospective multicenter clinical study of the Osseotite implant: four-year interim report. Int J Oral Maxillofac Implants. 2001 Mar-Apr;16(2):193-200.
311. Thoma DS, Haas R, Sporniak-Tutak K, Garcia A, Taylor TD, Hämmerle CHF. Randomized controlled multicentre study comparing short dental implants (6 mm) versus longer dental implants (11-15 mm) in combination with sinus floor elevation procedures: 5-Year data. J Clin Periodontol. 2018 Dec;45(12):1465-1474.
312. Thomé G, Cartelli CA, Vianna CP, Trojan LC. Retrospective Clinical Study of 453 Novel Tapered Implants Placed in All Bone Types: Survival Rate Analysis Up to 2 Years of Follow-Up. Int J Oral Maxillofac Implants. 2020 Jul/Aug;35(4):757-761.
313. Thöne-Mühling M, Pricope L, Mogk M, Mengel R. Turned surface implants in patients treated for periodontitis: Preliminary 10- to 20-year results of a long-term cohort study. Int J Oral Implantol (Berl). 2020;13(2):173-184.
314. Todisco M, Sbricoli L, Ippolito DR, Esposito M. Do we need abutments at immediately loaded implants supporting cross-arch fixed prostheses? Results from a 5-year randomised controlled trial. Eur J Oral Implantol. 2018;11(4):397-407.
315. Trbakovic A, Bongenhielm U, Thor A. A clinical and radiological long-term follow-up study of narrow diameter implants in the aesthetic area. Clin Implant Dent Relat Res. 2018 Aug;20(4):598-605.
316. Uraz A, Isler SC, Cula S, Tunc S, Yalim M, Cetiner D. Platform-switched implants vs platform-matched implants placed in different implant-abutment interface positions: A prospective randomized clinical and microbiological study. Clin Implant Dent Relat Res. 2020 Feb;22(1):59-68.
317. van Steenberghe D, Lekholm U, Bolender C, Folmer T, Henry P, Herrmann I, Higuchi K, Laney W, Linden U, Astrand P. Applicability of osseointegrated oral implants in the rehabilitation of partial edentulism: a prospective multicenter study on 558 fixtures. Int J Oral Maxillofac Implants. 1990 Fall;5(3):272-81.
318. Vandeweghe S, De Bruyn H. The effect of smoking on early bone remodeling on surface modified Southern Implants®. Clin Implant Dent Relat Res. 2011 Sep;13(3):206-14.
319. Vasak C, Kohal RJ, Lettner S, Rohner D, Zechner W. Clinical and radiological evaluation of a template-guided (NobelGuide™) treatment concept. Clin Oral Implants Res. 2014 Jan;25(1):116-23.
320. Veltri M, Ferrari M, Balleri P. One-year outcome of narrow diameter blasted implants for rehabilitation of maxillas with knife-edge resorption. Clin Oral Implants Res. 2008 Oct;19(10):1069-73.

321. Vercruyssen M, Marcelis K, Coucke W, Naert I, Quirynen M. Long-term, retrospective evaluation (implant and patient-centred outcome) of the two-implants-supported overdenture in the mandible. Part 1: survival rate. Clin Oral Implants Res. 2010 Apr 1;21(4):357-65.
322. Verdugo F, Uribarri A, Laksmana T, D'addona A. Long-term stable vertical bone regeneration after sinus floor elevation and simultaneous implant placement with and without grafting. Clin Implant Dent Relat Res. 2017 Dec;19(6):1054-1060.
323. Vervaeke S, Collaert B, Cosyn J, Deschepper E, De Bruyn H. A multifactorial analysis to identify predictors of implant failure and peri-implant bone loss. Clin Implant Dent Relat Res. 2015 Jan;17 Suppl 1:e298-307.
324. Vervaeke S, Collaert B, De Bruyn H. Immediate loading of implants in the maxilla: survival and bone loss after at least 2 years in function. Int J Oral Maxillofac Implants. 2013 Jan-Feb;28(1):216-21.
325. Vervaeke S, Collaert B, Vandeweghe S, Cosyn J, Deschepper E, De Bruyn H. The effect of smoking on survival and bone loss of implants with a fluoride-modified surface: a 2-year retrospective analysis of 1106 implants placed in daily practice. Clin Oral Implants Res. 2012 Jun;23(6):758-766.
326. Vigolo P, Givani A, Majzoub Z, Cordioli G. Clinical evaluation of small-diameter implants in single-tooth and multiple-implant restorations: a 7-year retrospective study. Int J Oral Maxillofac Implants. 2004 Sep-Oct;19(5):703-9.
327. Villa R, Rangert B. Early loading of interforaminal implants immediately installed after extraction of teeth presenting endodontic and periodontal lesions. Clin Implant Dent Relat Res. 2005;7 Suppl 1:S28-35.
328. Vogl S, Stopper M, Hof M, Wegscheider WA, Lorenzoni M. Immediate Occlusal versus Non-Occlusal Loading of Implants: A Randomized Clinical Pilot Study. Clin Implant Dent Relat Res. 2015 Jun;17(3):589-97.
329. Walton TR. The Up-to-14-Year Survival and Complication Burden of 256 TiUnite Implants Supporting One-Piece Cast Abutment/Metal-Ceramic Implant-Supported Single Crowns. Int J Oral Maxillofac Implants. 2016 Nov/Dec;31(6):1349-1358.
330. Wang X, Qin L, Lei C, Li Y, Li D. Effects of uncontrolled periodontitis on marginal bone alterations around implants: A case-control study. Clin Implant Dent Relat Res. 2017 Aug;19(4):654-662.
331. Wang YC, Kan JY, Rungcharassaeng K, Roe P, Lozada JL. Marginal bone response of implants with platform switching and non-platform switching abutments in posterior healed sites: a 1-year prospective study. Clin Oral Implants Res. 2015 Feb;26(2):220-7.
332. Weerapong K, Sirimongkolwattana S, Sastraruji T, Khongkhunthian P. Comparative study of immediate loading on short dental implants and conventional dental implants in the posterior mandible: A randomized clinical trial. Int J Oral Maxillofac Implants. 2019 January/February;34(1):141–149.
333. Weng D, Jacobson Z, Tarnow D, Hürzeler MB, Faehn O, Sanavi F, Barkvoll P, Stach RM. A prospective multicenter clinical trial of 3i machined-surface implants: results after 6 years of follow-up. Int J Oral Maxillofac Implants. 2003 May-Jun;18(3):417-23.
334. Wennerberg A, Jemt T. Complications in partially edentulous implant patients: a 5-year retrospective follow-up study of 133 patients supplied with unilateral maxillary prostheses. Clin Implant Dent Relat Res. 1999;1(1):49-56.
335. Widbom C, Söderfeldt B, Kronström M. A retrospective evaluation of treatments with implant-supported maxillary overdentures. Clin Implant Dent Relat Res. 2005;7(3):166-72.
336. Winkler S, Morris HF, Ochi S. Implant survival to 36 months as related to length and diameter. Ann Periodontol. 2000 Dec;5(1):22-31.
337. Wolfinger GJ, Balshi TJ, Wulc DA, Balshi SF. A retrospective analysis of 125 single molar crowns supported by two implants: long-term follow-up from 3 to 12 years. Int J Oral Maxillofac Implants. 2011 Jan-Feb;26(1):148-53.
338. Wu S, Wu X, Shrestha R, Lin J, Feng Z, Liu Y, Shi Y, Huang B, Li Z, Liu Q, Zhang X, Hu M, Chen Z. Clinical and Radiologic Outcomes of Submerged and Nonsubmerged Bone-Level Implants with Internal Hexagonal Connections in Immediate Implantation: A 5-Year Retrospective Study. J Prosthodont. 2018 Feb;27(2):101-107.
339. Wu X, Al-Abedalla K, Abi-Nader S, Daniel NG, Nicolau B, Tamimi F. Proton Pump Inhibitors and the Risk of Osseointegrated Dental Implant Failure: A Cohort Study. Clin Implant Dent Relat Res. 2017 Apr;19(2):222-232.
340. Wyatt CC, Zarb GA. Treatment outcomes of patients with implant-supported fixed partial prostheses. Int J Oral Maxillofac Implants. 1998 Mar-Apr;13(2):204-11.
341. Yamada J, Kori H, Tsukiyama Y, Matsushita Y, Kamo M, Koyano K. Immediate loading of complete-arch fixed prostheses for edentulous maxillae after flapless guided implant placement: a 1-year prospective clinical study. Int J Oral Maxillofac Implants. 2015 Jan-Feb;30(1):184-93.
342. Yang G, Chen L, Gao Y, Liu H, Dong H, Mou Y. Risk factors and reoperative survival rate of failed narrow-diameter implants in the maxillary anterior region. Clin Implant Dent Relat Res. 2020 Feb;22(1):29-41.
343. Yi YJ, Lee JY, Kim YK. Comparative clinical study of three-unit fixed partial prostheses supported by two or three implants. Int J Oral Maxillofac Implants. 2013 Jul-Aug;28(4):1110-5.
344. Yildiz P, Zortuk M, Kiliç E, Dinçel M, Albayrak H. Clinical Outcomes After Immediate and Late Implant Loading for a Single Missing Tooth in the Anterior Maxilla. Implant Dent. 2016 Aug;25(4):504-9.

345. Yu H, Wang X, Qiu L. Outcomes of 6.5-mm Hydrophilic Implants and Long Implants Placed with Lateral Sinus Floor Elevation in the Atrophic Posterior Maxilla: A Prospective, Randomized Controlled Clinical Comparison. Clin Implant Dent Relat Res. 2017 Feb;19(1):111-122.
346. Zadeh HH, Guljé F, Palmer PJ, Abrahamsson I, Chen S, Mahallati R, Stanford CM. Marginal bone level and survival of short and standard-length implants after 3 years: An Open Multi-Center Randomized Controlled Clinical Trial. Clin Oral Implants Res. 2018 Aug;29(8):894-906.
347. Zembić A, Glauser R, Khraisat A, Hämmerle CH. Immediate vs. early loading of dental implants: 3-year results of a randomized controlled clinical trial. Clin Oral Implants Res. 2010 May;21(5):481-9.
348. Zhang XM, Shi JY, Gu YX, Qiao SC, Mo JJ, Lai HC. Clinical Investigation and Patient Satisfaction of Short Implants Versus Longer Implants with Osteotome Sinus Floor Elevation in Atrophic Posterior Maxillae: A Pilot Randomized Trial. Clin Implant Dent Relat Res. 2017 Feb;19(1):161-166.
349. Zhou N, Dong H, Zhu Y, Liu H, Zhou N, Mou Y. Analysis of implant loss risk factors especially in maxillary molar location: A retrospective study of 6977 implants in Chinese individuals. Clin Implant Dent Relat Res. 2019 Feb;21(1):138-144.
350. Zill A, Precht C, Beck-Broichsitter B, Sehner S, Smeets R, Heiland M, Rendenbach C, Henningsen A. Implants inserted with graftless osteotome sinus floor elevation - A 5-year post-loading retrospective study. Eur J Oral Implantol. 2016;9(3):277-289.
351. Zumstein T, Billström C, Sennerby L. A 4- to 5-year retrospective clinical and radiographic study of Neoss implants placed with or without GBR procedures. Clin Implant Dent Relat Res. 2012 Aug;14(4):480-90.
352. Zumstein T, Sennerby L. A 1-Year Clinical and Radiographic Study on Hydrophilic Dental Implants Placed with and without Bone Augmentation Procedures. Clin Implant Dent Relat Res. 2016 Jun;18(3):498-506.
353. Zweers J, van Doornik A, Hogendorf EA, Quirynen M, Van der Weijden GA. Clinical and radiographic evaluation of narrow- vs. regular-diameter dental implants: a 3-year follow-up. A retrospective study. Clin Oral Implants Res. 2015 Feb;26(2):149-56.

Table S1. Detailed data of the included studies.

Study	Year	Study design	Country / Setting	Patients (male/ female) (n)	Patients' Age Range (mean) (years)	Loading	Implant location	Implant used
Adanez	2018	PS (unicenter)	Germany / University	10 (NM)	NM	Delayed (2 mo)	Md	Superline Fixture (Dentium, Seoul, Korea)
Al-Nawas	2015	PS (multicenter)	Spain / mainly Private practice	359 (126/233)	20-80	Several protocols	Mx, Md	Roxolid (Straumann, Basel, Switzerland)
Alayan	2019	PS (multicenter)	Australia / University + Private practice	53 (16/37)	NM (61)	Delayed (3 mo)	Mx	SLA (Straumann, Basel, Switzerland)
Alsaadi (1)	2007	RA (unicenter)	Belgium / University	2004 (792/1212)	NM	NM	Mx, Md	Machined and TiUnite (Brånemark, Nobel Biocare, Göteborg, Sweden)
Alsaadi (2)	2008	RA (unicenter)	Belgium / University	412 (172/240)	NM	NM	Mx, Md	Machined and TiUnite (Brånemark, Nobel Biocare, Göteborg, Sweden)
Amato	2020	RA (multicenter)	Italy / Private practice	55 (17/38)	30-86 (63)	Immediate	Mx, Md	T3 (Zimmer Biomet, Warsaw, USA)
Amorfini	2017	RCT (unicenter)	Italy / University	26 (8/18)	39-75 (55.7)	Immediate	Mx	Bone Level SLA active (Straumann, Basel, Switzerland)
Andersson	2015	RA (multicenter)	Sweden / Private practice	55 (27/28)	NM	Early (1-3 d)	MX, Md	Bimodal and Proactive (Neoss Ltd, Harrogate, UK)
Anitua (1)	2008	RA (multicenter)	Spain / Private practice	1060 (386/674)	17-91 (54)	Immediate, delayed (3 mo)	Mx, Md	BTI (BTI Biotechnology Institute, Vitoria-Gasteiz, Spain)
Anitua (2)	2016	RA (unicenter)	Spain / Private practice	20 (2/18)	27-71 (54.05)	Delayed (>3 mo)	Mx, Md	NM (BTI Biotechnology Institute, Vitoria-Gasteiz, Spain)
Anitua (3)	2019	RA (unicenter)	Spain / Private practice	16 (3/13)	NM (57)	Immediate	Mx, Md	NM (BTI Biotechnology Institute, Vitoria-Gasteiz, Spain)
Aparicio	1998	RA (unicenter)	Spain / Private	45 (26/19)	34-82 (51.3)	Delayed (4-6	Mx, Md	Brånemark (Nobel Biocare, Göteborg, Sweden)

			practice			mo)		
Arlin	2006	RA (unicenter)	Canada / Private practice	264 (131/133)	13.8-95.7 (55.5)	Delayed (3-5 mo)	Mx, Md	NM (Straumann, Basel, Switzerland)
Arora	2018	PS (unicenter)	Asutralia / Private practice	30 (13/17)	26-77 (52)	Delayed (3-4 mo)	Mx	NM (Straumann, Basel, Switzerland)
Arosio	2018	RA (multicenter)	Italy / Private practice	106 (56/50)	36-72 (58.4)	Delayed	Mx	Stone (IDI Evolution, Concorezzo, Italy)
Artzi	2010	RA (unicenter)	Israel / University	54 (23/31)	34-81 (57.5)	Immediate	Mx, Md	DFI, ITO, SPI (Alpha-Bio Tec, Israel)
Arunjaroen suk	2018	RCT (unicenter)	Thailand / University	60 (21/39)	21-78 (51.2)	Not loaded	Mx	Bone Level SLA active (Straumann, Basel, Switzerland)
Atieh	2014	PS (unicenter)	New Zealand / University	28 (10/18)	NM (50.3)	Immediate	Md	MAX (Southern Implants, Irene, South Africa)
Babbush	2012	RA (unicenter)	USA / Private Practice	293 (172/121)	NM (59)	Immediate, delayed	Mx, Md	NobelActive (Nobel Biocare, Göteborg, Sweden)
Bae	2015	RA (unicenter)	South Korea / Private practice	85 (42/43)	21-78 (58.1)	Delayed (4-6 mo)	Mx	Several (Brånemark, Astra, Osstem, Implantium Dentium)
Bahat (1)	1992	RA (unicenter)	USA / Private practice	45 (NM)	<80 (NM)	Delayed	Mx	Brånemark (Nobel Biocare, Göteborg, Sweden)
Bahat (2)	1996	RA (unicenter)	USA / Private practice	45 (NM)	NM	Delayed	Mx, Md	Brånemark (Nobel Biocare, Göteborg, Sweden)
Bahat (3)	2000	RA (unicenter)	USA / Private practice	202 (78/124)	18-81 (NM)	Delayed (6 mo)	Mx	Brånemark (Nobel Biocare, Göteborg, Sweden)
Bain	1993	RA (unicenter)	Scotland / Private practice	540 (229/311)	12-86 (55)	Delayed	Mx, Md	Brånemark (Nobel Biocare, Göteborg, Sweden)
Balleri (1)	2002	RA (unicenter)	Italy / University	14 (5/9)	NM (50.1)	NM	Mx, Md	Brånemark (Nobel Biocare, Göteborg, Sweden)
Balleri (2)	2010	PS (unicenter)	Italy / University	20 (6/14)	38-63 (51)	Delayed (6 mo)	Mx	Microthread and Osseospeed (Astra Tech, Mölndal, Sweden)
Balshi (1)	1997	RA	USA, Sweden, Belgium	71 (NM)	18-77 (54)	Delayed (3-5	Mx, Md	Brånemark (Nobel Biocare, Göteborg, Sweden)

		(multicenter)	/ Private practice			mo)		
Balshi (2)	1999	RA (unicenter)	USA / Private practice	189 (67/122)	28-91 (60)	Delayed	Mx	Brånemark (Nobel Biocare, Göteborg, Sweden)
Balshi (3)	2005	PS (unicenter)	USA / Private practice	55 (24/31)	25-86 (57.3)	Immediate	Mx	Brånemark (Nobel Biocare, Göteborg, Sweden)
Balshi (4)	2015	RA (unicenter)	USA / Private practice	470 (153/317)	11-86 (56.62)	Immediate, delayed	Md	Brånemark and TiUnite (Nobel Biocare, Göteborg, Sweden)
Bechara	2017	RCT (unicenter)	Lithuania / University	53 (19/34)	21-76 (48)	Delayed (4 mo)	Mx	AnyRidge (MegaGen Implant, Gyeongbuk, South Korea)
Becker (1)	2000	PS (multicenter)	NM / Private practice	83 (35/48)	18-80 (NM)	Delayed (3-6 mo)	Mx, Md	Brånemark MS (Nobel Biocare, Göteborg, Sweden) and TPS (ITI Straumann, Waldenburg, Switzerland)
Becker (2)	2005	PS (multicenter)	USA, Sweden, Israel / Private practice	57 (24/33)	24-86 (NM)	Delayed	Mx, Md	TiUnite (Nobel Biocare, Göteborg, Sweden)
Becker (3)	2013	PS (NM)	NM / NM	76 (38/38)	17-85 (NM)	Delayed (4 mo)	Mx, Md	Neoss (Neoss Ltd., Harrogate, UK)
Becktor (1)	2002	RA (multicenter)	USA, Sweden / Hospital	90 (31/59)	31-74 (57.4)	Delayed (4-12 mo)	Mx	NM (Nobel Biocare, Göteborg, Sweden)
Becktor (2)	2004	RA (unicenter)	Sweden / Hospital	182 (94/88)	31-79 (NM)	Delayed	Mx	Brånemark (Nobel Biocare, Göteborg, Sweden)
Becktor (3)	2007	RA (multicenter)	Sweden, Norway / NM	77 (34/46)	44-89 (64.5)	Delayed (3-4 mo)	Md	Brånemark (Nobel Biocare, Göteborg, Sweden)
Benlidayi	2018	RA (unicenter)	Turkey / University	38 (19/19)	30-71 (48.3)	Delayed (2-3 mo)	Mx, Md	NM (Nucleoss Implants, Izmir, Turkey)
Bergendal	1998	PS (multicenter)	Sweden / Private practice	49 (16/33)	7-82 (66)	Delayed	Mx, Md	Brånemark (Nobel Biocare, Göteborg, Sweden)
Bernard	2019	RCT (unicenter)	Belgium / University	60 (NM)	31-78 (NM)	Delayed (3 mo)	Mx, Md	Astra Tech TX (Dentsply Sirona, , Mölndal, Sweden)
Bernardi	2018	RCT (unicenter)	Italy / University	36 (18/18)	43-77 (62)	NM	Md	ConicalActive and IM Macon (Maco Dental Care,

								Salerno, Italy)
Bianco	2000	RA (multicenter)	Italy / Private practice	214 (100/114)	16-70 (NM)	Delayed	Mx, Md	Brånemark (Nobel Biocare, Göteborg, Sweden)
Bischof	2006	RA (unicenter)	Switzerland / Private practice	212 (91/121)	22-88 (49.9)	Delayed (mean 3.7 mo)	Mx, Md	wide neck ITI (ITI Straumann, Waldenburg, Switzerland)
Blomqvist	1998	PS (unicenter)	Sweden / Hospital	50 (17/33)	31-83 (59)	Delayed (6-10 mo)	Mx	Brånemark Mark II (Nobel Biocare, Göteborg, Sweden)
Blus	2006	RA (NM)	Italy / Private practice	57 (28/29)	23-82 (50.2)	Delayed (5-6 mo)	Mx, Md	Osseotite (Biomet 3i, Palm Beach Gardens, USA) and Leader (Milano, Italy)
Bogaerde (1)	2003	PS (multicenter)	Italy / Private practice	31 (13/18)	23-71 (51)	Immediate, early (within 20 days)	Mx, Md	Brånemark (Nobel Biocare, Göteborg, Sweden)
Bogaerde (2)	2004	PS (multicenter)	Italy / Private practice	31 (13/18)	36-78 (54)	Early (9-16 days)	Mx, Md	TiUnite (Nobel Biocare, Göteborg, Sweden)
Bogaerde (3)	2005	PS (unicenter)	Italy / Private practice	19 (5/14)	35-66 (55)	Immediate, early	Mx, Md	MK IV TiUnite (Nobel Biocare, Göteborg, Sweden)
Bolle	2018	RCT (multicenter)	France / University + Private practice	80 (38/42)	25-77 (NM)	Delayed (4 mo)	Mx, Md	TwinKon Universal SA2, Global-D
Boon	2020	PS (unicenter)	Belgium / University	153 (59/94)	NM (50.8)	Delayed (3 mo)	Mx, Md	NobelActive (Nobel Biocare, Göteborg, Sweden)
Borges	2020	RCT (unicenter)	Portugal / Private practice	33 (10/23)	NM (60.5)	Delayed (2 mo)	Md	OsseoSpeed EV (Astra Tech, Dentsply Implants, Mölndal, Sweden)
Bornstein	2008	RA (unicenter)	Switzerland / University	1206 (573/633)	18-92 (55.2)	Delayed (6-14 wk)	Mx, Md	SLA (Straumann, Basel, Switzerland)
Bouhy	2020	PS (unicenter)	Belgium / University	30 (17/13)	48-82 (66.4)	Delayed (3 mo)	Mx	Roxolid (Straumann, Basel, Switzerland)
Bressan	2014	PS (unicenter)	Italy / University	25 (10/15)	52-80 (69)	Immediate	Md	Ankylos (Dentsply Friadent, Mannheim, Germany)

Browaeys	2013	RA (unicenter)	Belgium / University	83 (51/32)	28-89 (58.2)	Immediate, early	Mx, Md	Osseotite (Biomet 3i, Palm Beach Gardens, USA)
Brügger	2015	RA (unicenter)	Switzerland / University	1568 (776/792)	17-92 (53.6)	Delayed (4-14 wk)	Mx, Md	SLA active (Straumann, Basel, Switzerland)
Brånemark	1995	RA (unicenter)	Sweden / NM	156 (56/100)	20-80 (NM)	Delayed (3-8 mo)	Mx, Md	Brånemark (Nobel Biocare, Göteborg, Sweden)
Buser	1997	PS (multicenter)	Switzerland, Germany / University	1003 (406/597)	15-91 (52)	Delayed (3-6 mo)	Mx, Md	Several (ITI Straumann, Waldenburg, Switzerland)
Caban	2017	RA (unicenter)	Sweden / University	25 (11/14)	44-84 (71)	Delayed (2-6 mo)	Mx	Microthread (Astra Tech, Mölndal, Sweden)
Calandriello	2011	PS (multicenter)	Italy / Private practice	33 (16/17)	27-72 (52)	Immediate	Md	Brånemark and TiUnite (Nobel Biocare, Göteborg, Sweden)
Calvo-Guirado	2016	PS (unicenter)	Spain / University	10 (4/6)	44-86 (64)	Delayed (3 mo)	Md	Tissue level SLActive and Roxolid (Straumann, Basel, Switzerland)
Campos	2019	PS (unicenter)	Brazil / University	34 (NM)	NM (51.33)	Not loaded	Md	NM (Neodent, Curitiba, Brazil)
Cannizzaro (1)	2012	RCT (unicenter)	Italy / Private practice	50 (24/26)	18-71 (38.8)	Immediate	Mx, Md	NanoTite (Biomet 3i, Palm Beach Gardens, USA)
Cannizzaro (2)	2013	RCT (unicenter)	Italy / Private practice	40 (19/21)	21-72 (50)	Delayed (1.5 mo)	Mx	Tapered Screw-Vent MP-1 HA Dual Transition Selective Surface (Zimmer Dental)
Cannizzaro (3)	2016	RCT (unicenter)	Italy / Private practice	40 (21/19)	33-78 (56)	Immediate	Mx, Md	Prama PF tapered (Sweden & Martina, Due Carrare, Italy)
Cannizzaro (4)	2018	RCT (unicenter)	Italy / Private practice	30 (15/15)	38-80 (60)	Immediate	Mx, Md	NanoTite (Biomet 3i, Palm Beach Gardens, USA)
Carr (1)	2003	RA (unicenter)	USA / Non-profit organization	308 (139/169)	15-95 (NM)	Delayed	Mx, Md	NM (Straumann, Basel, Switzerland)
Carr (2)	2019	RA (unicenter)	USA / Non-profit	2798 (NM)	>18	Immediate,	Mx, Md	NM

			organization			delayed		
Checchi	2010	PS (multicenter)	Italy / Private practice	15 (7/8)	28-68 (55)	Delayed (6 mo)	Mx	TSV Screw-Vent tapered and MTX microtextured (Zimmer Dental)
Cecchinato	2015	RCT (multicenter)	Italy, Switzerland, Spain/ University + Private practice	93 (48/45)	19-80 (51)	Delayed (4 mo)	Mx	Osseospeed (Dentsply Implants, Mölndal, Sweden)
Chiapasco	2014	RA (unicenter)	Italy / NM	50 (16/34)	19-69 (49.5)	Delayed (2-3 mo)	Mx, Md	Bone Level and Tissue Level (Straumann, Basel, Switzerland)
Clelland	2016	RCT (unicenter)	USA / NM	18 (9/9)	49-76 (56)	Delayed (3-5 mo)	Mx, Md	Osseospeed (Dentsply Implants, Mölndal, Sweden)
Cochran (1)	2002	PS (multicenter)	USA, Switzerland, Netherlands, Germany / University + Hospital	133 (57/76)	20-82 (NM)	Delayed (42-105 d)	Mx, Md	ITI and SLA (Straumann, Basel, Switzerland)
Cochran (2)	2007	PS (multicenter)	Several / Private practice	509 (230/278, 1NM)	14-90 (52.5)	Delayed (6-8 w)	Mx, Md	SLA (Straumann, Basel, Switzerland)
Collaert (1)	2002	NM (unicenter)	Belgium / Private practice	25 (5/20)	28-88 (NM)	Early (within 1 mo)	Md	TiOblast (Astra Tech, Mölndal, Sweden)
Collaert (2)	2011	PS (unicenter)	Belgium / University	25 (10/15)	39-78 (60.4)	Immediate	Md	Osseospeed (Dentsply Implants, Mölndal, Sweden)
Correia	2017	RA (unicenter)	Portugal / Private practice	202 (70/132)	23-73 (50)	Immediate, delayed	Mx, Md	Several (Straumann, Nobel Biocare, Biomet 3i, Neodent, Klockner, EuroTeknika)
Cosyn	2012	RA (unicenter)	Belgium / University	461 (216/245)	18-90 (51)	Immediate, early, delayed	Mx, Md	NM (Nobel Biocare, Göteborg, Sweden), NM (Straumann, Basel, Switzerland), NM (Dentsply Friadent , Mannheim, Germany), NM (Astra Tech Mölndal, Sweden), NM (Biomet 3i, Palm Beach Gardens, USA)

Cucchi	2019	PS (NM)	Italy / NM	20 (NM)	NM (67)	Early (3-5 d)	Mx, Md	NM
Dagorne	2015	RA (unicenter)	France / Private practice	59 (NM)	NM (57.6)	NM	Mx, Md	Nobel Speedy Groovy and Brånemerk Mk III (Nobel Biocare, Göteborg, Sweden)
Dahlin	2013	PS (multicenter)	Sweden / University + Private practice	177 (72/105)	<20 - >81 (NM)	Delayed	Mx, Md	Neoss (Neoss Ltd., Harrogate, UK)
Davarpanah	2002	PS (multicenter)	France / ”13 clinical centers”	528 (193/335)	16-86 (53.6)	Delayed (4-6 mo)	Mx, Md	Osseotite, ICE, and self-tapping (Biomet 3i, Palm Beach Gardens, USA)
De Bruyn (1)	1992	RA (unicenter)	NM / NM	57 (NM)	19-72 (53)	Delayed (3-6 mo)	Mx, Md	Screw Vent (Core-Vent Co., Encino, USA)<br>Brånemark (Nobel Biocare, Göteborg, Sweden)
De Bruyn (2)	2002	PS (NM)	NM / NM	36 (18/18)	63-81 (NM)	Immediate, early (<52 days)	Md	Machined Brånemark (Nobel Biocare, Göteborg, Sweden)
Degasperi	2014	RA (NM)	Italy / Private practice	49 (20/49)	29-79 (50.9)	Delayed (3-4 mo)	Mx, Md	Neoss Proactive (Neoss Ltd, Harrogate, UK)
Degidi (1)	2005	NM (NM)	Italy / NM	253 (106/147)	20-78 (53)	Immediate	Mx, Md	XiVE (Dentsply Friadent, Mannheim, Germany)
Degidi (2)	2006	NM (unicenter)	Italy / University	371 (180/191)	17-83 (53)	Immediate, delayed	Mx, Md	XiVE (Dentsply Friadent, Mannheim, Germany)
Degidi (3)	2009	NM (unicenter)	Italy / University	155 (71/84)	18-78 (54)	Immediate	Mx, Md	Maestro (BioHorizons, Birmingham, USA)
Degidi (4)	2012	RA (unicenter)	Italy / Private practice	1045 (368/677)	18-93 (NM)	Not loaded	Mx, Md	XiVE (Dentsply Friadent, Mannheim, Germany)
Deporter (1)	2001	PS (unicenter)	Canada / NM	50 (25/25)	25-76 (53.7)	Delayed	Mx	Endopore (Innova, Corportaion, Toronto, Ontario, Canada)
Deporter (2)	2014	PS (NM)	Canada / NM	52 (17/35)	NM (55.3)	Delayed (10 wk)	Md	SPS (Innova Corporation, Toronto, ON, Canada)
Diaz-	2017	CCT (unicenter)	Spain /University	27 (13/14)	NM (52.7)	Delayed (4 mo)	Mx, Md	Element Inicell (Thommen Medical AG,

Sanchez								Waldenburg, Germany)
Ducommun	2019	RA (unicenter)	Switzerland / University	1428 (700/728)	NM (57.2)	Delayed (1-4 mo)	Mx, Md	Several (Straumann, Thommen, Zeramex)
Eckert	2001	RA (unicenter)	USA / Non-profit organization	63 (35/28)	19-81 (52.2)	Delayed (3-4 mo)	Mx, Md	MK II (Nobel Biocare, Göteborg, Sweden)
Eliasson	2009	RA (unicenter)	Sweden / Public Service	109 (53/56)	47-89 (69)	Early, Delayed	Md	Brånemark MK III, TiUnite (Nobel Biocare, Göteborg, Sweden), TiOblast (Astra Tech, Mölndal, Sweden), ITI MonoType SLA (ITI Straumann, Waldenburg, Switzerland)
Engstrand	2003	PS (unicenter)	Sweden / Public Service	95 (53/42)	45-89 (68.5)	Immediate, early (1-40 days)	Md	Brånemark (Nobel Biocare, Göteborg, Sweden)
Esposito (1)	2015	RCT (multicenter)	Italy / Hospital	28 (16/12)	29-65 (54)	Delayed (4 mo)	Mx	ExFeel and Rescue (MegaGen Implant, Gyeongbuk, South Korea)
Esposito (2)	2016	RCT (multicenter)	Italy, South Korea / Private practice	120 (47/73)	20-79 (52)	Immediate, delayed	Mx, Md	EZ Plus (MegaGen Implant, Gyeongbuk, South Korea)
Esposito (3)	2017	RCT (unicenter)	Switzerland / Private practice	23 (11/12)	34-76 (54)	Delayed (3-6 mo)	Mx, Md	SPI Element (Thommen Medical, Waldenburg, Switzerland)
Esposito (4)	2019	RCT (multicenter)	Italy / Private practice	80 (25/55)	39-80 (57)	Delayed (4 mo)	Mx	ExFeel and Rescue (MegaGen Implant, Gyeongbuk, South Korea)
Evian	1996	RA (unicenter)	USA / Private practice	166 (57/109)	18-81 (NM)	Delayed (6 mo)	Mx, Md	Micro-Vent HA-coated and Swede vent (Dentsply, Encino, USA)
Felice (1)	2014	RCT (multicenter)	Italy / Private practice	64 (35/29)	19-80 (52)	Immediate, early, delayed	Mx, Md	Way Milano and Kentron (Geass srl, Pozzuolo Del Friuli, Udine, Italy)
Felice (2)	2015	RCT (multicenter)	Italy / Private practice	50 (25/25)	32-72 (52)	Immediate, delayed (4 mo)	Mx	XiVE S plus (Dentsply Friadent, Mannheim, Germany)
Felice (3)	2019	RCT	Italy / Private practice	30 (13/17)	37-70 (56)	Delayed (4 mo)	Mx, Md	EZ Plus and Rescue (MegaGen Implant,

		(multicenter)	+ hospital					Gyeongbuk, South Korea)
Felice (4)	2019	RCT (multicenter)	Italy / Private practice + hospital	40 (21/19)	42-80 (55)	Delayed (4 mo)	Mx, Md	NM (Southern implants, Irene, South Africa)
Feng	2014	RA (unicenter)	China / University	21 (12/9)	21-58 (45.9)	Delayed (4-9 mo)	Mx	Tissue Level (Straumann, Basel, Switzerland)
Fenlon	2002	PS (unicenter)	England / University	16 (7/9)	32-74 (NM)	Delayed (3 mo)	Md	Mark II Brånemark (Nobel Biocare, Göteborg, Sweden)
Fermergård	2012	RA (unicenter)	Sweden / Hospital	36 (NM)	NM (64)	Delayed (3-4 mo)	Mx	NM (Astra Tech Mölndal, Sweden)
Ferrigno (1)	2002	PS (multicenter)	Italy / University	233 (93/140)	35-79 (59.4)	Delayed (6wk-6mo)	Mx, Md	ITI (Straumann, Waldenburg, Switzerland)
Ferrigno (2)	2006	PS (unicenter)	Italy / University	323 (142/181)	34-73 (51.2)	Delayed (6wk-6mo)	Mx	ITI (Straumann, Waldenburg, Switzerland)
Fischer	2008	RCT (multicenter)	Sweden / Hospital	24 (8/16)	NM (64)	Early, Delayed	Mx	Esthetic Plus SLA (Straumann, Basel, Switzerland)
Fortin (1)	2002	RA (unicenter)	Canada / Private parctice	45 (15/30)	18-80 (NM)	Delayed	Mx	Brånemark (Nobel Biocare, Göteborg, Sweden)
Fortin (2)	2017	RA (unicenter)	Canada / Private practice	44 (NM)	NM	Delayed (5-6 mo)	Mx	NM
Friberg (1)	1992	PS (unicenter)	Sweden / Public service	30 (13/17)	32-82 (62)	Delayed (3-6 mo)	Mx, Md	Brånemark (Nobel Biocare, Göteborg, Sweden)
Friberg (2)	1997	PS (multicenter)	Sweden / Public service + University	103 (49/54)	33-83 (59)	Delayed (3-6 mo)	Mx, Md	MK II and standard Brånemark (Nobel Biocare, Göteborg, Sweden)
Friberg (3)	2002	RA (unicenter)	Sweden / Public service	98 (35/63)	NM (62)	Delayed (4-9 mo)	Mx, Md	Brånemark (Nobel Biocare, Göteborg, Sweden)
Friberg (4)	2003	PS	Sweden / Public	44 (15/29)	40-80 (65)	Delayed (3-6	Mx, Md	Standard Brånemark and MKIV (Nobel Biocare,

		(multicenter)	service			mo)		Göteborg, Sweden)
Friberg (5)	2005	PS (multicenter)	Sweden, Finland, Norway / Public service + hospital + University	187 (NM)	16-86 (53)	Immediate, Delayed	Mx, Md	TiUnite (Nobel Biocare, Göteborg, Sweden)
Friberg (6)	2008	RA (unicenter)	Sweden / Public service	75 (39/36)	42-87 (65.1)	Delayed	Mx	Brånemark (Nobel Biocare, Göteborg, Sweden)
Fugazzotto	2002	RA (unicenter)	USA / Private practice	103 (42/61)	31-72 (NM)	Delayed (6-12 wk)	Mx	Non self-taping (Straumann, Basel, Switzerland), Self-taping (Implant Innovations, West Palm Beach, USA)
Gallucci (1)	2004	RA (unicenter)	Switzerland / University	8 (4/4)	45-63 (NM)	Immediate	Mx, Md	ITI (Straumann, Waldenburg, Switzerland)
Gallucci (2)	2009	PS (multicenter)	USA / University	45 (19/26)	34-78 (59.5)	Delayed (3-5 mo)	Md	ITI (Straumann, Basel, Switzerland)
Garcez-Filho	2015	RS (unicenter)	Brazil / Private practice	21 (9/12)	33-78 (55.5)	Delayed (6-8 wk)	Mx	SLActive (Straumann, Basel, Switzerland)
Garlini	2003	RA (unicenter)	Italy / University	244 (106/138)	24-75 (NM)	Early (2 wk)	Mx, Md	Osseotite (Biomet 3i, Palm Beach Gardens, USA)
Gastaldi	2017	RCT (multicenter)	Italy / Private practice	60 (29/31)	25-81 (53)	Early (3 wk)	Mx	Xpeed (MegaGen Implant, Gyeongbuk, South Korea)
Geckili	2014	RA (unicenter)	Turkey / University	616 (269/347)	NM (52.7)	Delayed	Mx, Md	NM (Straumann, Waldenburg, Switzerland), NM (Astra Tech, Mölndal, Sweden), Biolok (Biohorizons, Birmighamn, USA), XiVe (Dentsply-Friadent, Mannheim, Germany)
Gholami	2014	RA (unicenter)	Switzerland / University	20 (10/10)	NM (62.1)	Delayed (3-4 mo)	Mx, Md	Element Inicell (Thommen Medical AG, Waldenburg, Germany)
Glauser (1)	2001	PS (NM)	NM / University	41 (19/22)	19-72 (52)	Immediate	Mx, Md	Mk IV Brånemark (Nobel Biocare, Göteborg, Sweden)

Glauser (2)	2003	PS (NM)	NM / University	38 (17/21)	19-77 (51)	Immediate	Mx, Md	Mk IV TiUnite Brånemark (Nobel Biocare, Göteborg, Sweden)
Glibert	2016	CCT (unicenter)	Belgium / University	48 (18/30)	>18	Immediate, delayed (10 wk)	Mx, Md	Osseotite 2 Certain (Biomet 3i, Palm Beach Gardens, USA)
Golab	2016	PS (multicenter)	NM / Private practice	272 (119/153)	18-76 (56.3)	Immediate	Mx, Md	Dr Nik OPS (S&S Biomat Ltd)
Gopalakrishnan	2014	PS (unicenter)	India / University	13 (8/5)	>18	Delayed (6 mo)	Mx, Md	Laser Lok (BioHorizons, Birmingham, USA)
Gotfredsen	1993	RA (unicenter)	Denmark / University	20 (5/15)	54-78 (65)	Delayed (4-9 mo)	Md	NM (Astra Tech, Mölndal, Sweden)
Goto	2002	RA (unicenter)	Japan / University	36 (26/10)	20-83 (52.9)	NM	Mx, Md	NM
Grunder	1999	PS (multicenter)	Several / Private practice	143 (68/75)	NM (44)	Delayed	Mx, Md	NM (Nobel Biocare, Göteborg, Sweden)
Guarnieri (1)	2014	RA (multicenter)	Italy / Private practice	46 (24/22)	26-60 (45.5)	Immediate	Mx	Laser Lok (BioHorizons, Birmingham, USA)
Guarnieri (2)	2016	RA (NM)	Italy / Private practice	25 (15/10)	NM (42)	Immediate	Mx	Laser Lok (BioHorizons, Birmingham, USA)
Guarnieri (3)	2019	PS (unicenter)	Italy / University	30 (16/14)	21-82 (51)	Delayed (4-6 mo)	Mx, Md	Laser Lok (BioHorizons, Birmingham, USA)
Guarnieri (4)	2019	RCT (unicenter)	Italy / University	20 (12/8)	36-64 (49.7)	Delayed (4-6 mo)	Mx, Md	Laser Lok (BioHorizons, Birmingham, USA)
Guida	2020	RCT (multicenter)	Italy / Private practice + hospital + University	30 (17/13)	NM (63)	Delayed (3 mo)	Md	OsseoSpeed TX (Astra Tech, Dentsply Sirona, Mölndal, Sweden)
Gulje (1)	2013	RCT (multicenter)	Several / University + Private practice	95 (48/47)	26-70 (54)	Delayed (42-48 d)	Mx, Md	Osseospeed (Astra Tech, Mölndal, Sweden)

Gulje (2)	2019	RCT (multicenter)	Netherlands / Private practice	38 (18/20)	29-72 (49)	Delayed (3 mo)	Mx	OsseoSpeed TX (Astra Tech, Dentsply Sirona, Mölndal, Sweden)
Haas	1996	RA (unicenter)	Austria / University	607 (246/361)	21-86 (51)	Delayed (3-6 mo)	Mx, Md	IMZ (Friatec, Friedrichseld, Germany)
Hallman (1)	2001	PS (unicenter)	Sweden / Hospital	40 (15/25)	19-86 (57)	Delayed (3-6 mo)	Mx	TPS (ITI, Straumann, Waldenburg, Switzerland)
Hallman (2)	2004	RA (unicenter)	Sweden / Hospital	50 (22/28)	23-82 (61)	Early, Delayed (10d-10 mo)	Mx	SLA (Straumann, Waldenburg, Switzerland)
Hallman (3)	2005	RA (unicenter)	Sweden / Hospital	22 (NM)	41-77 (60)	Delayed (6 mo)	Mx	Brånemark (Nobel Biocare, Göteborg, Sweden), TiOblast (Astra Tech, Mölndal, Sweden)
Han	2014	RA (unicenter)	South Korea / University	879 (NM)	NM	Immediate, delayed	Mx, Md	Several (Nobel Biocare, Straumann, Biolok International)
Hatano	2011	RA (unicenter)	Japan / Private practice	132 (65/67)	35-85 (62.6)	Immediate	Md	Brånemark machined and TiUnite (Nobel Biocare, Göteborg, Sweden)
Hattingh	2019	RA (unicenter)	United Kingdom / Private practice	85 (43/42)	45-87 (65)	Delayed (4 mo)	Mx, Md	MAX (Southern Implants, Irene, South Africa)
Hayacibara	2013	RA (unicenter)	Brazil / Private practice	71 (31/40)	22-85 (53)	Delayed (12 wk)	Md	NM (Straumann, Waldenburg, Switzerland)
He	2015	RA (unicenter)	China / University	1377 (704/673)	18-83 (44.9)	Immediate, delayed	Mx, Md	Several (Straumann, Bego, Dentium, Anthogyr, Biomet 3i, Lifecore, Osstem, BLB)
Hentenaar	2020	RA (unicenter)	Netherlands / University	64 (29/38)	NM	NM	Mx, Md	NM (Biomet 3i, Palm Beach Gardens, USA) and NM (Astra Tech, Mölndal, Sweden)
Hentschel	2015	RA (unicenter)	Germany / Private practice	416 (156/260)	20-84 (58)	Delayed (6-8 wk)	Mx, Md	SLActive (Straumann, Basel, Switzerland)
Herrero-Climent	2014	RCT (unicenter)	Spain / University	25 (NM)	NM	Delayed (2 mo)	Mx, Md	Essential Cone (Klockner Implant System)

Herrman	2005	RA (multicenter)	Sweden / University	487 (216/271)	NM (51.3)	Delayed	Mx, Md	Brånemark (Nobel Biocare, Göteborg, Sweden)
Higuchi	1995	PS (multicenter)	NM / NM	139 (59/80)	18-70 (NM)	Delayed	Mx, Md	NM (Nobel Biocare, Göteborg, Sweden)
Hoffmann	2006	RA (NM)	NM / NM	7 (3/4)	38-62 (NM)	Delayed	Mx, Md	ITI (Straumann, Waldenburg, Switzerland)
Hopp	2017	RA (unicenter)	Portugal / Private practice	891 (364/527)	20-85 (56)	Immediate	Mx	Brånemark MIII or MKIV, NobelSpeedy (Nobel Biocare, Göteborg, Sweden)
Horikawa	2017	RA (multicenter)	Japan / Private practice	92 (38/54)	20-78 (54.3)	NM	Mx, Md	TPS-type and Bonefit S-type (Straumann, Basel, Switzerland)
Hu	2020	RA (unicenter)	Singapore / Public Service	200 (100/100)	28-84 (57.7)	NM	Mx, Md	NM (Straumann, Basel, Switzerland)
Hussaini	2010	RA (multicenter)	UAE / Private practice	249 (115/134)	20-71 (45)	Delayed (2-5 mo)	Mx, Md	Several (Biohorizon, BioLok, Paragon, Astra, Nobel Biocare)
Imburgia	2015	RA (unicenter)	Italy / Private practice	41 (12/29)	19-79 (52.6)	Immediate, delayed (4-6 mo)	Mx, Md	Brånemark MIII TiUnite (Nobel Biocare, Göteborg, Sweden)
Ivanoff	1999	RA (unicenter)	Sweden / Public service	67 (30/37)	16-86 (59)	Delayed	Mx, Md	Brånemark (Nobel Biocare, Göteborg, Sweden)
Jang	2011	RA (unicenter)	South Korea / Hospital	3755 (3120/635)	40-79 (65)	Immediate, Delayed (>3 mo)	Mx, Md	Several (Astra Tech, BioHorizons, Altatec, Osstem, Zimmer Dental, Nobel Biocare)
Jemt (1)	1991	PS (multicenter)	Several / NM	92 (45/47)	14-70 (NM)	Delayed	Mx, Md	Turned Brånemark (Nobel Biocare, Göteborg, Sweden)
Jemt (2)	2006	RA (unicenter)	Sweden / Public service	76 (48/28)	32-75 (60.1)	Delayed (6-8 mo)	Mx	Turned Brånemark (Nobel Biocare, Göteborg, Sweden)

Jonker	2018	RCT (multicenter)	Netherlands / University + hospital	52 (30/22)	NM (47)	Delayed (2 mo)	Mx	Bone Level (Straumann, Basel, Switzerland)
Kahnberg	2008	PS (unicenter)	Sweden / University	36 (14/23)	NM (59)	Delayed (6 mo)	Mx	TiOblast ST (Astra Tech, Mölndal, Sweden)
Kaneda	2019	RA (unicenter)	Japan / University	52 (34/18)	NM (60.5)	Immediate	Md	NobelSpeedy Groovy, Brånemark System Mark III or Mark IV, Nobel Replace tapered (Nobel Biocare, Göteborg, Sweden)
Karlsson	1998	PS (multicenter)	Norway, Denmark, Sweden, Finland / University + Public service	50 (25/25)	NM (53)	Delayed (3-7 mo)	Mx, Md	Machined and TiOblast (Astra Tech, Mölndal, Sweden)
Keller	1999	RA (unicenter)	USA / Non-profit organization	62 (42/20)	15-73 (48)	Delayed	Mx	Brånemark (Nobel Biocare, Göteborg, Sweden)
Kennedy	2013	PS (unicenter)	USA / University	18 (9/9)	49-76 (62.4)	Delayed (3-5 mo)	Mx, Md	Osseospeed (Dentsply Implants, Mölndal, Sweden)
Khayat	2001	RA (unicenter)	NM / Private practice	71 (NM)	17-78 (57.8)	Delayed (3-6 mo)	Mx, Md	Screw-vent (Paragon, Encino, USA)
Khouly	2017	RA (unicenter)	Spain / Private practice	67 (30/37)	NM (56)	Delayed (6-9 mo)	Mx	BTI Externa (BTI, Biotechnology Institute, Vitoria-Gasteiz, Spain) and Brånemark Mk IV TiUnite (Nobel Biocare, Göteborg, Sweden)
Kim (1)	2015	RA (unicenter)	USA / Community practice	85 (43/42)	17-83 (55)	Immediate, delayed	Mx, Md	Nobel Replace Select (Nobel Biocare, Göteborg, Sweden)
Kim (2)	2018	RA (unicenter)	South Korea / University	881 (496/385)	17-90 (51.9)	Delayed	Mx, Md	Tissue Level (Straumann, Basel, Switzerland)
Kinsel	2007	RA (unicenter)	USA / Private practice	43 (12/31)	35-80 (58)	Immediate	Mx, Md	TPS and SLA (Straumann, Basel, Switzerland)
Kokovic	2014	RCT (unicenter)	Serbia / University	12 (3/9)	20-62 (49)	Immediate, early	Md	SLA TE (Straumann, Basel, Switzerland)
Koo	2010	RA (unicenter)	South Korea / Hospital	489	23-91 (47)	Delayed (3-6	Mx, Md	Mark III and TiUnite (Nobel Biocare, Göteborg,

				(298/191)		mo)		Sweden)
Kovacs	2000	RA (unicenter)	Germany / University	76 (56/20)	35-81 (52.39)	Delayed (3-6 mo)	Mx, Md	Bone-Lock (Howmedica Leibinger, Freiburg, Germany)
Lago	2018	RCT (unicenter)	Spain / University	100 (54/46)	25-70 (50.5)	Delayed (>2 mo)	Mx, Md	Standard Plus Type and Bone Level (Straumann, Basel, Switzerland)
Lai (1)	2008	RA (unicenter)	China / University	12 (8/4)	40-73 (56.3)	Early	Mx	ITI (Straumann, Waldenburg, Switzerland)
Lai (2)	2008	PS (unicenter)	China / University	32 (15/17)	20-65 (45.4)	Not loaded	Mx	SLA ITI (Straumann, Waldenburg, Switzerland)
Lai (3)	2010	RA (unicenter)	China / University	202 (92/110)	20-68 (47)	Delayed (3-4 mo)	Mx	NM (Straumann, Waldenburg, Switzerland)
Lee (1)	2013	RA (unicenter)	South Korea / University	338 (154/184)	20-85 (52.5)	NM	Mx, Md	Several (Brånemark, Replace, Straumann, Implantium, XiVe)
Lee (2)	2014	RA (unicenter)	South Korea / University	76 (37/39)	25-83 (56.3)	Delayed (3-6 mo)	Mx, Md	Osseospeed and ST (Astra Tech, Dentsply Implants, Mölndal, Sweden)
Lee (3)	2018	RA (unicenter)	Taiwan / Private practice	60 (27/33)	23-90 (53)	Immediate, delayed	Mx, Md	IDEOSS (IDEOSS Biotech, Taipei, Taiwan)
Lekholm	1999	PS (multicenter)	Australia, Belgium, Belgium, USA / NM	127 (55/72)	18-70 (50)	Delayed	Mx, Md	Brånemark (Nobel Biocare, Göteborg, Sweden)
Levine (1)	1997	RA (multicenter)	USA / NM	129 (44/85)	13-84 (52)	Delayed	Mx, Md	ITI (Straumann, Waldenburg, Switzerland)
Levine (2)	2007	RA (multicenter)	USA / Private practice	410 (182/228)	18-78 (NM)	Delayed (6wk-6 mo)	Mx, Md	Wide-neck (Straumann, Basel, Switzerland)
Lin	2018	RA (multicenter)	China / University + hospital	18199 (9027/9172)	17-91 (47.4)	Delayed (3-6 mo)	Mx, Md	Several (Straumann, Nobel Biocare, Ankylos, Bego, Osstem, Biconcept, SPI)
Lindgren	2012	PS (unicenter)	Sweden / Public service	11 (5/6)	50-79 (67)	Delayed (3-7 mo)	Mx	SLAactive (Straumann, Basel, Switzerland)

Lini	2019	RA (unicenter)	Italy / University	67 (45/22)	NM (65)	Delayed (2-6 mo)	Mx, Md	Frialit-2 (Friadent)
Lobato	2020	RCT (unicenter)	Brazil / University	44 (NM)	25-77 (50.8)	Delayed (4-6 mo)	Mx, Md	Alvim, Drive, and Titamax (Neodent, Curitiba, Brazil)
Lopez-Cedrun	2011	RA (unicenter)	Spain / Hospital	23 (4/19)	36-72 (NM)	Delayed (2-3 mo)	Md	ITI SLA (Straumann, Basel, Switzerland)
Lops	2013	PS (multicenter)	Italy / University	21 (NM)	23-50 (42)	Delayed (4 mo)	Mx	Bone-Level SLActive (Straumann, Basel, Switzerland)
Ma	2013	RA (unicenter)	China /University	514 (NM)	NM	NM	Mx, Md	NM (Straumann, Waldenburg, Switzerland), Replace (Nobel Biocare, Göteborg, Sweden)
Makkonen	1997	PS (unicenter)	Finland / University	33 (13/20)	39-75 (55)	Delayed (3-4 mo)	Md	Machined (Astra Tech, Mölndal, Sweden)
Malchiodi (1)	2011	RA (unicenter)	Italy / University	81 (NM)	NM	Immediate	Mx	FBR Pitt-easy (Oraltronics)<br>Osseotite Certain (Biomet 3i, Palm Beach Gardens, USA)
Malchiodi (2)	2017	PS (NM)	Italy / NM	43 (19/24)	41-79 (NM)	Delayed (5 mo)	Mx, Md	WINSIX (BioSAFin Srl, Italy)
Malmstrom	2016	PS (unicenter)	USA / University	30 (11/19)	22-80 (53.6)	Delayed (>3 mo)	Mx, Md	Osseospeed (Dentsply Implants, Mölndal, Sweden)
Malo (1)	2015	RA (unicenter)	Portugal / Private clinic	199 (81/118)	26-84 (53)	Immediate	Mx, Md	Machined or TiUnite (Nobel Biocare, Göteborg, Sweden)
Malo (2)	2015	RA (unicenter)	Portugal / Private practice	332 (143/189)	16-82 (47)	Immediate	Mx, Md	Mk II, Mk III, Mk IV, TiUnite, NobelSpeedy Groovy, NobelDirect, NobelReplace Tapered (Nobel Biocare, Göteborg, Sweden)
Mangano (1)	2014	PS (unicenter)	Italy / Private practice	642 (356/286)	20-82 (NM)	Delayed (3-4 mo)	Mx, Md	Leone (Sesto Fiorentino, Firenze, Italy)

Mangano (2)	2014	PS (unicenter)	Italy / Private practice	279 (159/120)	25-73 (48.1)	Delayed (3-4 mo)	Mx, Md	Sandblasted and acid-etched (Leone Implant System)
Manni	2020	RCT (unicenter)	Belgium / University	34 (10/24)	21-66 (47)	Delayed (4 mo)	Mx	C1 and V3 (MIS Implants Technologies Ltd)
Mattsson	1999	RA (NM)	Sweden / NM	15 (4/11)	44-75 (59)	Delayed	Mx	Brånemark (Nobel Biocare, Göteborg, Sweden)
McGlumphy	2003	PS (unicenter)	USA / University	121 (63/58)	18-79 (49)	Delayed (3-6 mo)	Mx, Md	NM
Mei	2017	PS (unicenter)	China / University	60 (18/42)	21-65 (46.9)	Delayed (2-4 mo)	Mx, Md	NM (Wego Jericom Biomaterials Co., Weihai, China)
Meloni	2018	RCT (unicenter)	Italy / University	20 (8/12)	28-70 (46)	Immediate, delayed	Md	NobelReplace Tapered Groovy (Nobel Biocare, Göteborg, Sweden)
Mendonca (1)	2014	RA (unicenter)	Brazil / Private practice	198 (86/112)	45-81 (60)	Delayed (12-18 wk)	Mx, Md	NM
Mendonca (2)	2017	RA (unicenter)	Brazil / Private practice	138 (54/84)	NM (57.6)	Delayed (12-18 wk)	Mx, Md	MK III (Nobel Biocare, Göteborg, Sweden)
Mendonca (3)	2017	RA (unicenter)	Brazil / Private practice	46 (16/30)	32-79 (57.8)	Delayed (12-18 wk)	Mx, Md	MK III (Nobel Biocare, Göteborg, Sweden)
Mericske-Stern	2002	RA (unicenter)	Switzerland / University	41 (17/24)	40-89 (61.2)	Delayed (4-6 mo)	Mx	NM (Nobel Biocare, Göteborg, Sweden)<br>ITI (Straumann, Waldenburg, Switzerland)
Merli	2008	RCT (unicenter)	Italy / Private practice	60 (22/38)	19-72 (49)	Immediate, early	Mx, Md	SPI Element (Thommen Medical, Waldenburg, Switzerland)
Mertens (1)	2011	PS (unicenter)	Germany / University	17 (5/12)	40-83 (61)	Early, Immediate	Mx, Md	Osseospeed (Astra Tech, Mölndal, Sweden
Mertens (2)	2012	PS (unicenter)	Germany / University	15 (5/10)	41-69 (55.3)	Delayed (6 mo)	Mx	TiOblast (Astra Tech, Mölndal, Sweden)
Mijiritsky	2013	RA (multicenter)	Israel / Private practice	787 (NM)	18-86 (53.7)	Immediate, delayed	Mx, Md	NM (Adin Dental Implants, Alon Tavor, Israel)

Mo	2015	RA (unicenter)	Norway / Private practice	12 (6/6)	(69)	Delayed (3-4 mo)	Mx	Osseospeed (Astra Tech, Mölndal, Sweden), Osstem (Osstem, Seoul, Korea), Straumann SLA tissue level and bone level (Straumann, Basel, Switzerland)
Mongardini	2020	RA (multicenter)	Italy / Private practice	263 (NM)	NM (60)	Delayed (3 mo)	Mx, Md	Way Milano (Geass srl, Pozzuolo Del Friuli, Udine, Italy)
Mozzati	2015	RA (unicenter)	Italy / Private practice	90 (41/49)	21-82 (55.9)	Immediate, delayed	Mx, Md	Brånemark MKIII and Mk IV TiUnite (Nobel Biocare, Göteborg, Sweden)
Mumcu	2019	RA (unicenter)	Turkey / University	57 (36/21)	NM (59.2)	Delayed (3 mo)	Md	Osseospeed TX (Astra Tech, Mölndal, Sweden)
Munakata	2016	RA (unicenter)	Japan / University	5 (3/2)	56-61 (58.2)	Delayed (7-9 mo)	Mx	NM
Nedir	2004	CCT (unicenter)	Switzerland / Private practice	236 (91/145)	18-89 (NM)	Delayed	Mx, Md	TPS, SLA (ITI Straumann, Basel, Switzerland)
Nevins	1993	RA (multicenter)	USA / Private practice	338 (NM)	18-89 (NM)	Delayed	Mx, Md	Brånemark (Nobel, Biocare, Göteborg, Sweden)
Niedemaier	2017	RA (unicenter)	Germany / Private practice	380 (188/192)	23-92 (61.9)	Immediate	Mx, Md	NanoTite (Biomet 3i, Palm Beach Gardens, USA), Nobel Active, Nobel Speedy, Nobel Speedy Replace and Nobel Replace CC (Nobel Biocare, Göteborg, Sweden)
Niimi	1998	RA (multicenter)	Japan, USA / University + non-profit organization	44 (NM)	NM	Delayed	Mx, Md	Brånemark (Nobel, Biocare, Göteborg, Sweden)
Nogueira	2018	PS (unicenter)	Brazil / University	45 (11/34)	48-80 (63.4)	Delayed (3 mo)	Md	Titamax (Neodent, Curitiba, Brazil)
Norton	2017	PS (unicenter)	United Kingdom / Private practice	22 (10/12)	22-79 (NM)	Immediate	Mx, Md	Atra Tech AV (Dentsply, Mölndal, Sweden)
Olate	2010	RA (unicenter)	Brazil / University	650 (221/429)	13-84 (42.7)	Delayed	Mx, Md	NM (Neodent, Curitiba, Brazil), NM (Conexao, São Paulo, Brazil), NM (SIN, São Paulo, Brazil)

Olson	2000	RA (multicenter)	USA / Veteran Medical Centers	89 (89/0)	40-78 (62.7)	Delayed (6-26 mo)	Mx, Md	NM (Paragon Implant, Encion, USA),<br>NM (Nobel Biocare, Göteborg, Sweden),<br>TPS (Interpore Corporation, Irvine, USA)
Olsson (1)	1995	CCT (multicenter)	Sweden / Public service + hospital + University	103 (49/54)	33-83 (59)	Delayed	Mx, Md	MK II and Standard Brånemark (Nobel Biocare, Göteborg, Sweden)
Olsson (2)	2003	NM (unicenter)	Denmark / Private practice	10 (4/6)	50-86 (59)	Early (1-9 d)	Mx	TiUnite Brånemark (Nobel Biocare, Göteborg, Sweden)
Ormianer (1)	2006	PS (unicenter)	Israel / Private practice	60 (23/37)	22-78 (55.4)	Immediate, Delayed (3-4 mo)	Mx, Md	Tapered screw-Vent MTX (Zimmer Dental Inc, Carlsbad, USA)
Ormianer (2)	2008	RA (multicenter)	Israel, USA / Private practice	60 (26/34)	18-78 (53)	Immediate, Delayed	Mx, Md	Tapered screw-Vent MTX (Zimmer Dental Inc, Carlsbad, USA)
Palmqvist	1994	RA (unicenter)	Sweden / Public service	25 (10/15)	39-77 (57.8)	Delayed	Mx	Brånemark (Nobel Biocare, Göteborg, Sweden)
Payne	2017	PS (multicenter)	New Zealand, Colombia, Netherlands / University + hospital	48 (21/27)	46-85 (NM)	Delayed (3 mo)	Md	SLA RN (Straumann, Basel, Switzerland)
Penarrocha -Diago	2008	RA (unicenter)	Spain / University	100 (45/55)	20-76 (47.5)	Delayed	Mx, Md	Impladent (Sentmenat, Barcelona, Spain)
Penarrocha -Oltra (1)	2012	RA (unicenter)	Spain / University	70 (32/38)	34-75 (54)	Delayed (8-10 wk)	Mx	Phybo Avantblast TSA (Impladent)
Penarrocha -Oltra (2)	2014	RA (unicenter)	Spain / University	37 (12/25)	27-60 (48.4)	Delayed (5-8 mo)	Md	TSA (Phibo Dental Solutions)
Penarrocha -Oltra (3)	2015	CCT (unicenter)	Spain / University	34 (15/19)	28-73 (54.6)	Immediate, delayed (2 mo)	Md	Kohno SP (Sweden & Martina)

Pettersson	2015	RA (unicenter)	Sweden / Private practice	88 (32/56)	NM (65)	Delayed (3-4 mo)	Mx, Md	Replace Select TiUnite (Nobel Biocare, Göteborg, Sweden)
Piano	2016	PS (unicenter)	Italy / Private practice	22 (9/13)	56-81 (66)	Immediate	Mx	SLActive Bone Level (Straumann, Basel, Switzerland)
Pico	2019	RCT (unicenter)	Spain / University	33 (NM)	40-76 (54)	Delayed (3 mo)	Mx, Md	BioniQ (LASAK)
Pieri	2016	RA (multicenter)	Italy / Private practice	101 (29/72)	NM (58)	Delayed (3-4 mo)	Mx	Osseospeed (Astra Tech, Mölndal, Sweden)
Pinholt	2003	RA (unicenter)	Denmark / Hospital	25 (7/18)	20-78 (52)	Delayed (8 mo)	Mx	Brånemark (Nobel Biocare, Göteborg, Sweden) and ITI (Straumann, Basel, Switzerland)
Pistilli (1)	2013	RCT (multicenter)	Italy / Private practice + hospital	40 (10/30)	39-80 (55)	Delayed (4 mo)	Mx, Md	Rescue and ExFeel (MegaGen Implant, Gyeongbuk, South Korea)
Pistilli (2)	2013	RCT (multicenter)	Italy / Private practice + hospital + University	40 (21/19)	42-80 (55)	Delayed (4 mo)	Mx, Md	NM (Southern, Irene, South Africa)
Pjetursson	2009	PS (unicenter)	Switzerland / University	181 (NM)	17-90 (54.9)	Delayed (4-6 mo)	Mx	NM (Straumann, Basel, Switzerland)
Polizzi	2000	PS/RA (multicenter)	Sweden, Italy / Private practice, public service	51 (NM)	29-79 (NM)	Delayed (4-9 mo)	Mx, Md	Wide Platform Brånemark (Nobel Biocare, Göteborg, Sweden)
Pozzi (1)	2014	RA (unicenter)	Italy / Private practice	73 (32/41)	19-76 (50.59)	Immediate, delayed	Mx, Md	MK III, MK IV, Nobel Replace Select Tapered (Nobel Biocare, Göteborg, Sweden)
Pozzi (2)	2014	RCT (multicenter)	Italy / Private practice	51 (29/22)	28-84 (63)	Immediate	Mx, Md	Nobel Såeedy Groovy (Nobel Biocare, Göteborg, Sweden)
Priest	1999	RA (unicenter)	USA / Private practice	99 (41/58)	15-76 (42)	Delayed (3-6 mo)	Mx, Md	NM (Biomet 3i, Palm Beach Gardens, USA), Brånemark (Nobel Biocare, Göteborg, Sweden), Steri-Oss (Steri-Oss, Yorba Linda, USA), NM (Friatec, Irvine, USA)
Prosper	2010	RA (unicenter)	Italy / University	71 (35/36)	26-72 (58.3)	Immediate, Delayed (3 mo)	Md	NM (Bioactive Covering, Winsix)

Qian	2020	RCT (unicenter)	China / University	45 (NM)	NM	Delayed (6 mo)	Mx	SLA (Straumann, Basel, Switzerland)
Queiroz	2015	PS (unicenter)	Brazil / University	23 (5/18)	42-69 (53)	Delayed (3 mo)	Md	Master Porous (Conexão Sistemas de Prótese Ltda, São Paulo, Brazil)
Queridinha	2016	RA (unicenter)	Portugal / Private practice	60 (21/39)	NM (64.1)	Immediate	Mx	Mk II, Mk III, Mk IV, TiUnite, NobelSpeedy Groovy, NobelReplace Tapered (Nobel Biocare, Göteborg, Sweden)
Rammelsberg	2014	RA (unicenter)	Germany / University	61 (39/22)	43-86 (65.4)	Delayed (3-9 mo)	Mx, Md	NM (Straumann, Basel, Switzerland)
Renouard	1999	RA (NM)	NM / NM	74 (30/44)	NM (54)	Delayed	Mx, Md	Brånemark (Nobel Biocare, Göteborg, Sweden)
Riben	2016	RA (unicenter)	Sweden / University	36 (17/19)	28-81 (65)	Delayed (6 mo)	Mx	TiOblast and Osseospeed (Dentsply implants, Mölndal, Sweden)
Rocci (1)	2003	RA (NM)	Italy / Private practice	46 (20/26)	24-77 (51)	Immediate	Mx	Mk IV and Machined Brånemark (Nobel Biocare, Göteborg, Sweden)
Rocci (2)	2003	RCT (NM)	Italy / Private practice	44 (30/14)	20-69 (51)	Immediate	Md	TiUnite and Machined Brånemark (Nobel Biocare, Göteborg, Sweden)
Roccuzzo (1)	2001	CCT (unicenter)	Italy / Private practice	32 (9/23)	26-59 (NM)	Early	Mx, Md	SLA and TPS ITI (Straumann, Waldenburg, Switzerland)
Roccuzzo (2)	2002	PS (unicenter)	Italy / Private practice	19 (7/12)	33-65 (NM)	Early (43 d)	Mx	SLA (Straumann, Waldenburg, Switzerland)
Romanos (1)	2000	RA (unicenter)	Germany / University	51 (29/22)	NM (45.1)	Delayed	Mx, Md	Ankylos (Degussa-Huls, Hanau, Germany)
Romanos (2)	2012	RA (NM)	NM / NM	55 (20/35)	40-84 (63.51)	immediate	Md	Ankylos (Dentsply)
Romanos (3)	2014	RA (unicenter)	USA / University	27 (15/12)	NM (59)	Immediate	Mx, Md	Ankylos (Dentsply implants, Mölndal, Sweden)
Romeo (1)	2004	PS (unicenter)	Italy / University	250	20-67 (NM)	Delayed (3-	Mx, Md	ITI (Straumann, Waldenburg, Switzerland)

				(106/144)		6mo)		
Romeo (2)	2009	PS (multicenter)	Italy / University + Private practice	45 (18/27)	42-100 (63)	Delayed (3 mo)	Mx, Md	ITI (Straumann, Waldenburg, Switzerland)
Roos	1997	RA (unicenter)	Sweden / Public service	169 (NM)	20-85 (58)	Delayed	Mx, Md	Brånemark (Nobel Biocare, Göteborg, Sweden)
Rosenberg	2004	RA (unicenter)	USA / Private practice	334 (136/198)	NM (54)	Delayed (5-9 mo)	Mx, Md	Several (Nobel Biocare, Straumann, Biomet, 3i, Paragon)
Rossi	2016	RCT (multicenter)	Italy / Private practice	45 (24/21)	30-74 (48)	Delayed (6 wk)	Mx, Md	SLA (Straumann, Basel, Switzerland)
Salvi	2004	RCT (unicenter)	NM / NM	27 (11/16)	29-72 (48.3)	Early (2-6 wk)	Md	ITI, SLA (Straumann, Waldenburg, Switzerland)
Santis	2016	PS (multicenter)	Italy / University + Private practice	62 (27/35)	32-74 (57)	Delayed (3-4 mo)	Mx, Md	NobelActive (Nobel Biocare, Göteborg, Sweden)
Sbordone	2015	RA (unicenter)	Italy / NM	17 (9/8)	37-74 (53.4)	Delayed (6 mo)	Mx, Md	NM
Schincaglia (1)	2007	RCT (unicenter)	Italy / University	10 (6/4)	37-74 (61.3)	Immediate	Md	Machined and TiUnite (Nobel Biocare, Göteborg, Sweden)
Schincaglia (2)	2008	RCT (unicenter)	Italy / University	30 (9/21)	31-75 (50)	Immediate, delayed (3 mo)	Md	MK III TiUnite (Nobel Biocare, Göteborg, Sweden)
Schnitman (1)	1997	NM (NM)	USA / NM	10 (6/4)	48-78 (NM)	Immediate	Md	Brånemark (Nobel Biocare, Göteborg, Sweden)
Schnitman (2)	2011	RA (NM)	USA / NM	18 (NM)	NM	Immediate, Delayed	Md	Osseotite (Biomet 3i, Palm Beach Gardens, USA)<br>MK III (Nobel Biocare, Göteborg, Sweden)<br>SLA Standard Plus (Straumann, Basel, Switzerland)
Scurria	1998	RA (unicenter)	USA / University	384 (112/272)	23-84 (60.5)	Delayed	Mx, Md	Brånemark (Nobel Biocare, Göteborg, Sweden), IMZ-plasma sprayed and HA-coated (Interpore Int., Irvine, USA)

Sener-Yamaner	2017	RA (unicenter)	Turkey / University	55 (34/21)	20-65 (50)	Delayed (3-8 wk)	Mx, Md	SLA and SLActive (Straumann, Basel, Switzerland)
Sennerby	2012	PS (multicenter)	NM / Private practice	90 (39/51)	22-82 (50.6)	Delayed (3-4 mo)	Mx, Md	Neoss Bimodal (Neoss, Harrogate, UK)
Sethi	2000	PS (unicenter)	United Kingdom / Private practice	150 (72/78)	NM	Delayed (6 mo)	Mx	NM
Shi (1)	2017	RA (unicenter)	China / Hospital	237 (131/106)	34-81 (57)	Delayed	Mx, Md	NM
Shi (2)	2019	RCT (unicenter)	China / Hospital	225 (96/129)	NM (41)	Delayed (3 mo)	Mx	Standard Plus (Straumann, Basel, Switzerland)
Shigehara	2015	RA (multicenter)	Japan / Private practice	27 (15/12)	NM (65)	Immediate	Mx, Md	Standard Plus and Tapered Effect SLA (Straumann, Basel, Switzerland)
Si	2016	RA (unicenter)	China / University	80 (43/37)	25-70 (48.8)	Delayed (3-4 mo)	Mx	NM (Straumann, Basel, Switzerland)
Smedberg	1991	RA (unicenter)	Sweden / Public service	20 (7/13)	43-78 (NM)	Delayed	Md	Brånemark (Nobel Biocare, Göteborg, Sweden)
Sohn	2010	RA (multicenter)	South Korea / University + Private practice	43 (NM)	NM	NM	Md	Endopore (Innova Life Science, Toronto, Canada)
Souza (1)	2018	RCT (unicenter)	Brazil / University	22 (12/10)	NM (59.2)	Delayed (6 wk)	Mx, Md	Standard Plus SLA Tissue Level (Straumann, Basel, Switzerland)
Souza (2)	2019	RA (unicenter)	Brazil / NM	10 (4/6)	49-70 (60)	Delayed (6 mo)	Mx	Master Porous (Conexão Sistemas de Prótese Ltda, São Paulo, Brazil)
Stanford	2016	RCT (multicenter)	Several / University + Private practice	120 (57/63)	21-75 (52)	Delayed (5-6 wk)	Mx, Md	Osseospeed EV and TX (Astra Tech, Mölndal, Sweden)
Steveling	2001	RA (unicenter)	Germany / University	17 (11/6)	27-72 (46.8)	Delayed (3 mo)	Mx	NM (Astra Tech, Mölndal, Sweden)
Storelli	2018	RCT	Italy / Private practice	24 (12/12)	32-75 (54.2)	Delayed (6 wk)	Mx, Md	SLA (Straumann, Basel, Switzerland)

		(multicenter)						
Tallarico (1)	2017	RCT (unicenter)	Italy / Private practice	24 (8/16)	37-67 (53)	Delayed (4 mo)	Mx, Md	Ultra-Wide (Osstem, Seoul, South Korea)
Tallarico (2)	2018	RCT (unicenter)	Italy / Private practice	20 (10/10)	28-84 (64)	Immediate	Mx, Md	Nobel Speedy Groovy (Nobel Biocare, Göteborg, Sweden)
Tang	2015	RA (unicenter)	China / University	157 (92/65)	17-74 (36.2)	Delayed (4-6 mo)	Mx, Md	NM (Nobel Biocare, Göteborg, Sweden)<br>NM (Straumann, Basel, Switzerland)
Taschieri (1)	2014	PS (multicenter)	Italy / University + Private practice	23 (15/8)	37-61 (55.8)	Delayed (5 mo)	Mx	NM (BTI Biotechnology Institute, Vitoria-Gasteiz, Spain)
Taschieri (2)	2018	RCT (unicenter)	Italy / University	52 (22/30)	31-77 (52)	Delayed (5-7 mo)	Mx	Interna, Universal Platform and Universal Plus Platform (BTI Biotechnology Institute, Vitoria-Gasteiz, Spain)
Tawil (1)	2001	RA (unicenter)	Lebanon / University	29 (20/9)	38-75 (56)	Delayed (6-9 mo)	Mx	Brånemark (Nobel Biocare, Göteborg, Sweden)
Tawil (2)	2002	RA (unicenter)	Lebanon / NM	60 (32/28)	24-81 (54)	Delayed (4-6 mo)	Mx, Md	RP (Nobel Biocare, Göteborg, Sweden)
Tawil (3)	2003	RA (NM)	Lebanon / NM	11 (45/66)	22-80 (53.6)	Delayed (4-6 mo)	Mx, Md	Machined (Nobel Biocare, Göteborg, Sweden)
Temmerman	2015	PS (unicenter)	Belgium / University	28 (3/25)	42-76 (63)	Delayed (mean 3.6 mo)	Mx, Md	NM (Astra Tech, Dentsply Implants, Mölndal, Sweden)
Testori (1)	2001	PS (multicenter)	Italy, Canada, Ireland, USA / Private Practice	181 (76/105)	18-86 (55.4)	Delayed (4-6 mo)	Mx, Md	Osseotite (Biomet 3i, Palm Beach Gardens, USA)
Testori (2)	2002	PS (multicenter)	Italy, USA, Brazil, Spain / University + Private practice	175 (79/96)	20-86 (53.5)	Delayed (2 mo)	Mx, Md	Osseotite (Biomet 3i, Palm Beach Gardens, USA)
Testori (3)	2004	RA (unicenter)	Italy / University	19 (10/9)	NM	Immediate, early (1 d)	Md	Osseotite (Biomet 3i, Palm Beach Gardens, USA)

Testori (4)	2017	RA (multicenter)	Italy / NM	27 (8/17)	39-69 (57)	Immediate	Mx	NM (Biomet 3i, Palm Beach Gardens, USA)
Thoma	2018	RCT (multicenter)	Switzerland, Poland, Austria, Spain, USA / University + Private practice	101 (49/52)	20-75 (50.5)	Delayed (5 mo)	Mx	Osseospeed (Astra Tech, Mölndal, Sweden)
Thome	2020	RA (unicenter)	Brazil / Private practice	101 (48/53)	NM (59.2)	Immediate, delayed	Mx, Md	Helix Acqua GM (Neodent, Curitiba, Brazil)
Thone-Muhling	2020	RA (unicenter)	Germany / University	39 (17/22)	NM	Delayed (3-6 mo)	Mx, Md	MK II and MK III (Nobel Biocare, Göteborg, Sweden)
Todisco	2018	RCT (unicenter)	Italy / Private practice	32 (16/14)	47-84 (62)	Immediate	Mx, Md	NobelActive (Nobel Biocare, Göteborg, Sweden)
Trbakovic	2018	RA (multicenter)	Sweden / University + Private practice	27 (10/17)	22-83 (57.6)	NM	Mx, Md	MK III (Nobel Biocare, Göteborg, Sweden), Standard Plus (Straumann, Basel, Switzerland), Osseospeed (Astra Tech, Mölndal, Sweden), Osseotite (Biomet 3i, Palm Beach Gardens, USA)
Uraz	2020	RCT (unicenter)	Turkey / University	70 (34/36)	NM (50)	Delayed (3 mo)	Mx, Md	Ankylos and XiVE (Dentsply implants, Mölndal, Sweden)
Vandewegh e	2011	RA (multicenter)	Belgium / Private practice	329 (141/188)	18-84 (54)	NM	Mx, Md	NM (Southern Implants, Irene, South Africa)
Vasak	2014	PS (multicenter)	Switzerland, Austria, Germany / University + hospital	30 (15/15)	31-80 (NM)	Immediate, Delayed (2-3 mo)	Mx, Md	NobelReplace, Tapered, Groovy (Nobel Biocare, Göteborg, Sweden)
Veltri	2008	PS (unicenter)	Italy / University	12 (4/8)	42-74 (58)	Delayed (6 mo)	Mx	TiOblasted Micro Thread (Astra Tech, Mölndal, Sweden)
Vercruysse n	2010	RA (multicenter)	Belgium / University + hospital	495 (152/343)	NM (60.8)	Delayed (3-5 mo)	Md	Brånemark (Nobel Biocare, Göteborg, Sweden)
Verdugo	2017	RA (unicenter)	NM / NM	30 (10/20)	NM (64.5)	Delayed (3-4 mo)	Mx	Standard Plus SLA (Straumann, Basel, Switzerland), Osseotite (Biomet 3i, Palm Beach

								Gardens, USA)
Vervaeke (1)	2012	RA (unicenter)	Belgium / Private Practice	300 (114/186)	17-82 (56)	Delayed (>3 mo)	Mx, Md	Osseospeed (Astra Tech, Mölndal, Sweden)
Vervaeke (2)	2013	RA (unicenter)	Belgium / University	55 (24/31)	19-77 (57.5)	Immediate	Mx	Osseospeed (Astra Tech, Mölndal, Sweden)
Vervaeke (3)	2015	RA (unicenter)	Belgium / Private Practice	376 (166/210)	17-82 (56)	Immediate, delayed	Mx, Md	Osseospeed (Astra Tech, Mölndal, Sweden)
Vigolo	2004	RA (unicenter)	Italy / Private Practice	165 (64/101)	NM (39)	Delayed (3-6 mo)	Mx, Md	NM (Biomet 3i, Palm Beach Gardens, USA)
Villa	2005	NM (unicenter)	Italy / Private Practice	20 (10/10)	49-70 (NM)	Early (<3 d)	Md	Brånemark (Nobel Biocare, Göteborg, Sweden)
Vogl	2015	PS (unicenter)	Austria / University	20 (7/13)	33-70 (54)	Immediate	Md	XiVE (Dentsply Friadent, Mannheim, Germany)
Walton	2016	PS (unicenter)	Australia / Private practice	184 (82/102)	15-79 (46.2)	Immediate, delayed (>3 mo)	Mx, Md	MK III, MK IV, Replace Select, NobelActive, Speedy Groovy TiUnite (Nobel Biocare, Göteborg, Sweden)
Wang (1)	2015	RCT (unicenter)	USA / University	19 (9/10)	23-76 (55.4)	Delayed (3 mo)	Mx, Md	Superline (DentiumUSA, Cypress, USA)
Wang (2)	2017	RA (unicenter)	China / University	60 (46/14)	NM (49.9)	Delayed (3-6 mo)	Mx, Md	SLA (Straumann, Basel, Switzerland)
Weerapong	2019	RCT (unicenter)	Thailand / University	46 (16/30)	20-64 (51)	Immediate	Md	NM (PW+ Dental Implant System)
Weng	2003	PS (multicenter)	Europe, USA / University + Private Practice	493 (240/253)	NM (45.1)	Delayed (4-6 mo)	MX, Md	Machined (Biomet 3i, Palm Beach Gardens, USA)
Wennerberg	1999	RA (unicenter)	Sweden / Public service	137 (58/79)	NM (60.8)	Delayed (5-6 mo)	Mx	Brånemark (Nobel Biocare, Göteborg, Sweden)
Widbom	2005	RA (unicenter)	Sweden / Hospital	27 (14/13)	NM (63.3)	Delayed	Mx	Brånemark (Nobel Biocare, Göteborg, Sweden)
Winkler	2000	RA (unicenter)	USA / Depart. Veterans Affairs	NM	NM	NM	Mx, Md	NM

Wolfinger	2011	RA (unicenter)	USA / Private Practice	105 (NM)	18-82 (54)	Immediate, delayed	Mx, Md	Several (Nobel Biocare, Göteborg, Sweden)
Wu (1)	2017	RA (unicenter)	Canada / University	799 (369/430)	18-93 (56.6)	NM	Mx, Md	TiUnite (Nobel Biocare, Göteborg, Sweden)
Wu (2)	2018	RA (unicenter)	China / University	72 (42/30)	NM (47)	Delayed (3-6 mo)	Mx, Md	XiVE S Plus (Dentsply implants, Mölndal, Sweden)
Wyatt	1998	PS (unicenter)	Canada / University	77 (30/47)	NM (45.14)	Delayed (3-26 mo)	Mx, Md	Brånemark (Nobel Biocare, Göteborg, Sweden)
Yamada	2015	PS (unicenter)	Japan / University	48 (26/22)	NM (56)	Immediate	Mx	NobleActive TiUnite (Nobel Biocare, Göteborg, Sweden)
Yang	2020	RA (unicenter)	China / University	835 (578/517)	18-80 (39.1)	Delayed (4-6 mo)	Mx	NM
Yi	2013	RA (unicenter)	South Korea / University	63 (31/32)	NM (60)	NM	Mx, Md	Several (Osseotite, Biomet 3i; TiUnite, Nobel Biocare; USII, USIII, GSII, GSIII, SSII, Osstem; Sinus Quick, Neobiotech; Implantium and Superline, Dentium)
Yildiz	2016	RA (unicenter)	Turkey / University	33 (10/23)	22-68 (NM)	Immediate, delayed (3 mo)	Mx	Bone Level SLA (Straumann, Basel, Switzerland)
Yu	2017	RCT (unicenter)	China / University	37 (20/17)	NM (49)	Delayed (2 mo)	Mx	Standard and Inicell (Thommen Medical AG, Grenchen, Switzerland)
Zadeh	2018	RCT (multicenter)	Sweden, USA, Netherlands, Australia / University + Private practice	95 (48/47)	26-70 (54)	Delayed (6 wk)	Mx, Md	Osseospeed (Astra Tech, Mölndal, Sweden)
Zembic	2010	RCT (unicenter)	Switzerland / University	11 (8/3)	37-68 (54.8)	Immediate, Early	Md	Brånemark MK IV, TiUnite (Nobel Biocare, Göteborg, Sweden)
Zhang	2017	RCT (unicenter)	China / University	56 (32/24)	NM (35.5-42.6)	Delayed (3 mo)	Mx	Standard Plus (Straumann, Basel, Switzerland)

Zhou	2019	RA (unicenter)	China / University	4338 (2063/2275)	18-87 (44.9)	Delayed (3-6 mo)	Mx	Several (NM)
Zill	2016	RA (unicenter)	Germany / Private practice	113 (NM)	31-84 (54.9)	Delayed (3 mo)	Mx	SLA (Straumann, Basel, Switzerland)
Zumstein (1)	2012	RA (unicenter)	United Kingdom / Private Practice	50 (19/31)	NM (57)	Delayed (3-6 mo)	Mx, Md	Neoss (Neoss Ltd. Harrogate, UK)
Zumstein (2)	2016	RA (unicenter)	Switzerland / Private practice	50 (22/28)	NM (58.6)	Immediate, delayed	Mx, Md	Proactive (Neoss Ltd, Harrogate, UK)
Zweers	2015	RA (unicenter)	Netherlands /Private Practice	119 (49/70)	34-93 (69)	Delayed (2 mo)	Md	SLA Standard (Straumann, Basel, Switzerland)
van Steenberghe	1990	PS (multicenter)	NM / NM	159 (67/92)	18-70 (NM)	NM	Mx, Md	Brånemark (Nobel Biocare, Göteborg, Sweden)
Örtorp	2008	RA (unicenter)	Sweden / Private Practice	104 (41/63)	27-78 (58)	Delayed (3-4 mo)	Md	Turned Brånemark (Nobel Biocare, Göteborg, Sweden)
Östman (1)	2008	PS (unicenter)	Sweden / NM	77 (38/39)	33-82 (NM)	Immediate	Md	TiUnite and turned Brånemark (Nobel Biocare, Göteborg, Sweden)
Östman (2)	2012	PS (unicenter)	Sweden / NM	46 (18/28)	NM	Immediate, delayed	Mx, Md	TiUnite MK III and MK IV (Nobel Biocare, Göteborg, Sweden)

NM – not mentioned; RA – retrospective analysis; PS – prospective study; CCT – controlled clinical trial; RCT – randomized controlled trial; d – days; wk – weeks; mo – months; Mx – maxilla; Md – mandible

Table S2. Quality assessment tool, according to the National Institutes of Health (NIH).

Items of the table:

1. Was the study question or objective clearly stated?
2. Was the study population clearly and fully described, including a case definition?
3. Were the cases consecutive?
4. Were the subjects comparable?
5. Was the intervention clearly described?
6. Were the outcome measures clearly defined, valid, reliable, and implemented consistently across all study participants?
7. Was the length of follow-up adequate?
8. Were the statistical methods well-described?
9. Were the results well-described?

Study	Year	1	2	3	4	5	6	7	8	9	Total (n/9)
Adanez	2018	1	0	0	1	1	1	1	1	1	7/9
Al-Nawas	2015	1	1	0	1	1	1	1	1	1	8/9
Alayan	2019	1	1	1	1	1	1	1	1	1	9/9
Alsaadi (1)	2007	1	1	1	1	1	1	1	1	1	9/9
Alsaadi (2)	2008	1	1	0	1	1	1	1	1	1	8/9
Amato	2020	1	1	0	1	1	1	1	0	1	7/9
Amorfini	2017	1	1	1	1	1	1	1	1	0	8/9
Andersson	2015	1	0	1	1	1	1	1	1	1	8/9
Anitua (1)	2008	1	1	1	1	1	1	1	1	1	9/9
Anitua (2)	2016	1	1	0	1	1	1	1	1	1	8/9
Anitua (3)	2019	1	1	0	1	1	1	1	1	1	8/9
Aparicio	1998	1	1	0	1	1	1	1	0	1	7/9
Arlin	2006	1	1	0	1	1	1	1	1	1	8/9
Arora	2018	1	1	0	1	1	1	1	1	1	8/9
Arosio	2018	1	1	0	1	1	1	1	1	0	7/9
Artzi	2010	1	1	0	1	1	1	1	1	1	8/9
Arunjaroensuk	2018	1	1	1	1	1	1	1	1	1	9/9
Atieh	2014	1	0	1	1	1	1	1	1	1	8/9
Babbush	2012	1	1	1	1	1	1	1	1	1	9/9
Bae	2015	1	1	0	1	1	1	1	1	0	7/9
Bahat (1)	1992	1	0	1	1	1	1	1	0	1	7/9
Bahat (2)	1996	1	0	0	1	1	1	1	0	1	6/9
Bahat (3)	2000	1	1	0	1	1	1	1	0	1	7/9
Bain	1993	1	1	0	1	1	1	1	0	1	7/9
Balleri (1)	2002	1	1	0	1	1	1	1	1	1	8/9
Balleri (2)	2010	1	1	0	1	1	1	1	1	1	8/9
Balshi (1)	1997	1	1	1	1	1	1	1	0	1	8/9
Balshi (2)	1999	1	1	0	1	1	1	1	0	1	7/9
Balshi (3)	2005	1	1	1	1	1	1	1	0	1	8/9
Balshi (4)	2015	1	1	0	1	1	1	1	1	1	8/9
Bechara	2017	1	1	1	1	1	1	1	1	1	9/9
Becker (1)	2000	1	1	0	1	1	1	1	0	1	7/9
Becker (2)	2005	1	1	0	1	1	1	1	1	1	8/9
Becker (3)	2013	1	1	1	1	1	1	1	1	1	9/9
Becktor (1)	2002	1	1	1	1	1	1	1	1	1	9/9
Becktor (2)	2004	1	1	1	1	1	1	1	1	1	9/9
Becktor (3)	2007	1	1	1	1	1	1	1	1	1	9/9
Benlidayi	2018	1	1	1	1	1	1	1	1	0	8/9
Bergendal	1998	1	1	0	1	1	1	1	1	1	8/9
Bernard	2019	1	1	1	1	1	1	1	1	1	9/9
Bernardi	2018	1	1	1	1	1	1	1	1	1	9/9

Bianco	2000	1	1	1	1	1	1	1	0	1	8/9
Bischof	2006	1	1	1	1	1	1	1	1	1	9/9
Blomqvist	1998	1	1	1	1	1	1	1	0	1	8/9
Blus	2006	1	1	1	1	1	1	1	0	1	8/9
Bogaerde (1)	2003	1	1	1	1	1	1	1	1	1	9/9
Bogaerde (2)	2004	1	1	1	1	1	1	1	1	1	9/9
Bogaerde (3)	2005	1	1	1	1	1	1	1	1	1	9/9
Bolle	2018	1	1	1	1	1	1	1	1	1	9/9
Boon	2020	1	1	1	1	1	1	1	1	0	8/9
Borges	2020	1	1	1	1	1	1	1	1	1	9/9
Bornstein	2008	1	1	1	1	1	1	1	1	1	9/9
Bouhy	2020	1	1	1	1	1	1	1	1	1	9/9
Bressan	2014	1	1	1	1	1	1	1	1	1	9/9
Browaeys	2013	1	1	0	1	1	1	1	0	1	7/9
Brügger	2015	1	1	1	1	1	1	1	1	1	9/9
Brånemark	1995	1	1	0	1	1	1	1	1	1	8/9
Buser	1997	1	1	1	1	1	1	1	1	1	9/9
Caban	2017	1	1	1	1	1	1	1	1	0	8/9
Calandriello	2011	1	1	1	1	1	1	1	1	1	9/9
Calvo-Guirado	2016	1	1	1	1	1	1	1	1	1	9/9
Campos	2019	1	1	0	1	1	1	1	1	0	7/9
Cannizzaro (1)	2012	1	1	1	1	1	1	1	1	1	9/9
Cannizzaro (2)	2013	1	1	1	1	1	1	1	1	1	9/9
Cannizzaro (3)	2016	1	1	1	1	1	1	1	1	1	9/9
Cannizzaro (4)	2018	1	1	1	1	1	1	1	1	1	9/9
Carr (1)	2003	1	1	0	1	1	1	1	1	1	8/9
Carr (2)	2019	1	1	1	1	1	1	1	1	1	9/9
Checchi	2010	1	1	1	1	1	1	1	1	1	9/9
Cecchinato	2015	1	1	0	1	1	1	1	1	0	7/9
Chiapasco	2014	1	1	0	1	1	1	1	0	1	7/9
Clelland	2016	1	1	0	1	1	1	1	1	1	8/9
Cochran (1)	2002	1	1	1	1	1	1	1	1	1	9/9
Cochran (2)	2007	1	1	0	1	1	1	1	1	1	8/9
Collaert (1)	2002	1	1	1	1	1	1	1	0	1	8/9
Collaert (2)	2011	1	1	1	1	1	1	1	1	1	9/9
Correia	2017	1	1	1	1	1	1	1	1	1	9/9
Cosyn	2012	1	1	1	1	1	1	1	1	1	9/9
Cucchi	2019	1	1	0	1	1	1	1	1	1	8/9
Dagorne	2015	1	1	0	1	1	1	1	1	0	7/9
Dahlin	2013	1	1	1	1	1	1	1	1	1	9/9
Davarpanah	2002	1	1	0	1	1	1	1	0	1	7/9
De Bruyn (1)	1992	1	1	1	1	1	1	1	1	1	9/9
De Bruyn (2)	2002	1	1	1	1	1	1	1	1	1	9/9
Degasperi	2014	1	1	0	1	1	1	1	0	1	7/9
Degidi (1)	2005	1	1	1	1	1	1	1	1	1	9/9
Degidi (2)	2006	1	1	1	1	1	1	1	1	1	9/9
Degidi (3)	2009	1	1	1	1	1	1	1	0	1	8/9
Degidi (4)	2012	1	1	0	1	1	1	1	1	1	8/9
Deporter (1)	2001	1	1	0	1	1	1	1	1	1	8/9
Deporter (2)	2014	1	1	0	1	1	1	1	1	1	8/9
Diaz-Sanchez	2017	1	1	0	1	1	1	1	1	1	8/9
Ducommun	2019	1	1	1	1	1	1	1	1	1	9/9
Eckert	2001	1	1	0	1	1	1	1	1	1	8/9
Eliasson	2009	1	1	1	1	1	1	1	1	1	9/9
Engstrand	2003	1	1	1	1	1	1	1	1	1	9/9
Esposito (1)	2013	1	1	1	1	1	1	1	1	1	9/9
Esposito (2)	2015	1	1	1	1	1	1	1	1	1	9/9
Esposito (3)	2016	1	1	0	1	1	1	1	1	1	8/9
Esposito (4)	2017	1	1	1	1	1	1	1	1	1	9/9
Esposito (5)	2019	1	1	1	1	1	1	1	1	1	9/9

Evian	1996	1	1	0	1	1	1	1	0	1	7/9
Felice (1)	2014	1	1	0	1	1	1	1	1	1	8/9
Felice (2)	2015	1	1	1	1	1	1	1	1	1	9/9
Felice (3)	2019	1	1	1	1	1	1	1	1	1	9/9
Felice (4)	2019	1	1	1	1	1	1	1	1	1	9/9
Feng	2014	1	1	1	1	1	1	1	1	1	9/9
Fenlon	2002	1	1	0	1	1	1	1	1	1	8/9
Fermergård	2012	1	1	1	1	1	1	1	0	1	8/9
Ferrigno (1)	2002	1	1	1	1	1	1	1	1	1	9/9
Ferrigno (2)	2006	1	1	1	1	1	1	1	1	1	9/9
Fischer	2008	1	1	0	1	1	1	1	1	1	8/9
Fortin (1)	2002	1	1	1	1	1	1	1	0	1	8/9
Fortin (2)	2017	1	0	1	1	1	1	1	0	1	7/9
Friberg (1)	1992	1	1	0	1	1	1	1	1	1	8/9
Friberg (2)	1997	1	1	1	1	1	1	1	1	1	9/9
Friberg (3)	2002	1	1	1	1	1	1	1	1	1	9/9
Friberg (4)	2003	1	1	1	1	1	1	1	1	1	9/9
Friberg (5)	2005	1	1	0	1	1	1	1	1	1	8/9
Friberg (6)	2008	1	1	1	1	1	1	1	1	1	9/9
Fugazzotto	2002	1	1	0	1	1	1	1	0	1	7/9
Gallucci	2004	1	1	0	1	1	1	1	0	1	7/9
Gallucci	2009	1	1	0	1	1	1	1	1	1	8/9
Garcez-Filho	2015	1	1	0	1	1	1	1	1	1	8/9
Garlini	2003	1	1	0	1	1	1	1	0	1	7/9
Gastaldi	2017	1	1	1	1	1	1	1	1	1	9/9
Geckili	2014	1	1	0	1	1	1	1	1	1	8/9
Gholami	2014	1	1	0	1	1	1	1	1	1	8/9
Glauser (1)	2001	1	1	1	1	1	1	1	1	1	9/9
Glauser (2)	2003	1	1	1	1	1	1	1	1	1	9/9
Glibert	2016	1	0	1	1	1	1	1	1	1	8/9
Golab	2016	1	1	0	1	1	1	1	1	1	8/9
Gopalakrishnan	2014	1	1	1	1	1	1	1	1	1	9/9
Gotfredsen	1993	1	1	0	1	1	1	1	1	1	8/9
Goto	2002	1	1	0	1	1	1	1	1	1	8/9
Grunder	1999	1	1	0	1	1	1	1	1	1	8/9
Guarnieri (1)	2014	1	1	0	1	1	1	1	1	0	7/9
Guarnieri (2)	2016	1	1	0	1	1	1	1	1	1	8/9
Guarnieri (3)	2019	1	1	1	1	1	1	1	1	1	9/9
Guarnieri (4)	2019	1	1	0	1	1	1	1	1	1	8/9
Guida	2020	1	1	0	1	1	1	1	1	1	8/9
Gulje (1)	2013	1	1	0	1	1	1	1	1	1	8/9
Gulje (2)	2019	1	1	1	1	1	1	1	1	1	9/9
Haas	1996	1	1	1	1	1	1	1	1	1	9/9
Hallman (1)	2001	1	1	1	1	1	1	1	1	1	9/9
Hallman (2)	2004	1	1	1	1	1	1	1	1	1	9/9
Hallman (3)	2005	1	1	1	1	1	1	1	1	1	9/9
Han	2014	1	1	1	1	1	1	1	1	1	9/9
Hatano	2011	1	1	1	1	1	1	1	0	1	8/9
Hattingh	2019	1	1	0	1	1	1	1	1	1	8/9
Hayacibara	2013	1	1	1	1	1	1	1	1	1	9/9
He	2015	1	1	1	1	1	1	1	1	1	9/9
Hentenaar	2020	1	1	0	1	1	1	1	1	1	8/9
Hentschel	2015	1	1	1	1	1	1	1	1	1	9/9
Herrero-Climent	2014	1	0	0	1	1	1	1	1	1	7/9
Herrman	2005	1	1	1	1	1	1	1	1	1	9/9
Higuchi	1995	1	1	0	1	1	1	1	0	1	7/9
Hoffmann	2006	1	1	1	1	1	1	1	0	1	8/9
Hopp	2017	1	1	0	1	1	1	1	1	1	8/9
Horikawa	2017	1	1	0	1	1	1	1	1	0	7/9
Hu	2020	1	1	1	1	1	1	1	1	0	8/9

Hussaini	2010	1	1	1	1	1	1	1	1	1	9/9
Imburgia	2015	1	1	1	1	1	1	1	1	1	9/9
Ivanoff	1999	1	1	1	1	1	1	1	1	1	9/9
Jang	2011	1	1	0	1	1	1	1	1	1	8/9
Jemt (1)	1991	1	1	0	1	1	1	1	0	1	7/9
Jemt (2)	2006	1	1	1	1	1	1	1	1	1	9/9
Jonker	2018	1	1	0	1	1	1	1	1	0	7/9
Kahnberg	2008	1	1	1	1	1	1	1	0	1	8/9
Kaneda	2019	1	1	0	1	1	1	1	1	0	7/9
Karlsson	1998	1	1	1	1	1	1	1	1	1	9/9
Keller	1999	1	1	1	1	1	1	1	0	1	8/9
Kennedy	2013	1	0	0	1	1	1	1	1	1	7/9
Khayat	2001	1	1	1	1	1	1	1	0	1	8/9
Khouly	2017	1	1	1	1	1	1	1	1	0	8/9
Kim (1)	2015	1	1	0	1	1	1	1	1	1	8/9
Kim (2)	2018	1	1	1	1	1	1	1	1	1	9/9
Kinsel	2007	1	1	1	1	1	1	1	1	1	9/9
Kokovic	2014	1	1	1	1	1	1	1	1	1	9/9
Koo	2010	1	1	0	1	1	1	1	1	1	8/9
Kovacs	2000	1	1	0	1	1	1	1	0	1	7/9
Lago	2018	1	1	0	1	1	1	1	1	0	7/9
Lai (1)	2008	1	1	0	1	1	1	1	1	1	8/9
Lai (2)	2008	1	1	0	1	1	1	1	1	1	8/9
Lai (3)	2010	1	1	0	1	1	1	1	1	1	8/9
Lee (1)	2013	1	1	1	1	1	1	1	1	1	9/9
Lee (2)	2014	1	1	0	1	1	1	1	1	1	8/9
Lee (3)	2018	1	1	0	1	1	1	1	1	0	7/9
Lekholm	1999	1	1	0	1	1	1	1	1	1	8/9
Levine (1)	1997	1	1	0	1	1	1	1	0	1	7/9
Levine (2)	2007	1	1	0	1	1	1	1	0	1	7/9
Lin	2018	1	1	1	1	1	1	1	1	1	9/9
Lindgren	2012	1	1	0	1	1	1	1	1	1	8/9
Lini	2019	1	1	1	1	1	1	1	1	0	8/9
Lobato	2020	1	1	0	1	1	1	1	1	0	7/9
Lopez-Cedrun	2011	1	0	1	1	1	1	1	0	1	7/9
Lops	2013	1	1	0	1	1	1	1	1	1	8/9
Ma	2013	1	0	1	1	1	1	1	1	1	9/9
Makkonen	1997	1	1	1	1	1	1	1	1	1	9/9
Malchiodi (1)	2011	1	0	1	1	1	1	1	1	1	8/9
Malchiodi (2)	2017	1	1	1	1	1	1	1	1	1	9/9
Malmstrom	2016	1	1	1	1	1	1	1	1	1	9/9
Malo (1)	2015	1	1	1	1	1	1	1	1	1	9/9
Malo (2)	2015	1	1	1	1	1	1	1	1	1	9/9
Mangano (1)	2014	1	1	0	1	1	1	1	1	1	8/9
Mangano (2)	2014	1	1	0	1	1	1	1	1	1	8/9
Manni	2020	1	1	0	1	1	1	1	1	1	8/9
Mattsson	1999	1	1	1	1	1	1	1	0	1	8/9
McGlumphy	2003	1	1	0	1	1	1	1	0	1	7/9
Mei	2017	1	1	0	1	1	1	1	1	1	8/9
Meloni	2018	1	1	1	1	1	1	1	1	1	9/9
Mendonca (1)	2014	1	1	0	1	1	1	1	1	1	8/9
Mendonca (2)	2017	1	1	0	1	1	1	1	1	1	8/9
Mendonca (3)	2017	1	1	0	1	1	1	1	1	1	8/9
Mericske-Stern	2002	1	1	1	1	1	1	1	1	1	9/9
Merli	2008	1	1	1	1	1	1	1	1	1	9/9
Mertens (1)	2011	1	1	0	1	1	1	1	1	1	8/9
Mertens (2)	2012	1	1	1	1	1	1	1	1	1	9/9
Mijiritsky	2013	1	1	1	1	1	1	1	0	1	8/9
Mo	2015	1	1	1	1	1	1	1	1	1	9/9
Mongardini	2020	1	1	0	1	1	1	1	1	0	8/9

Mozzati	2015	1	1	1	1	1	1	1	1	1	9/9
Mumcu	2019	1	1	0	1	1	1	1	1	1	8/9
Munakata	2016	1	1	0	1	1	1	1	1	1	8/9
Nedir	2004	1	1	1	1	1	1	1	0	1	8/9
Nevins	1993	1	1	0	1	1	1	1	0	1	7/9
Niedemaier	2017	1	1	0	1	1	1	1	1	1	8/9
Niimi	1998	1	0	0	1	1	1	1	1	1	7/9
Nogueira	2018	1	1	1	1	1	1	1	1	1	9/9
Norton	2017	1	1	1	1	1	1	1	1	1	9/9
Olate	2010	1	1	0	1	1	1	1	1	1	8/9
Olson	2000	1	1	0	1	1	1	1	1	1	8/9
Olsson (1)	1995	1	1	1	1	1	1	1	1	1	9/9
Olsson (2)	2003	1	1	1	1	1	1	1	1	1	9/9
Ormianer (1)	2006	1	1	1	1	1	1	1	1	1	9/9
Ormianer (2)	2008	1	1	0	1	1	1	1	1	1	8/9
Palmqvist	1994	1	1	1	1	1	1	1	1	1	9/9
Payne	2017	1	1	1	1	1	1	1	1	1	9/9
Penarrocha-Diago	2008	1	1	0	1	1	1	1	1	1	8/9
Penarrocha-Oltra (1)	2012	1	1	0	1	1	1	1	1	1	8/9
Penarrocha-Oltra (2)	2014	1	1	1	1	1	1	1	1	0	8/9
Penarrocha-Oltra (3)	2015	1	1	1	1	1	1	1	1	0	8/9
Petterson	2015	1	1	1	1	1	1	1	0	1	8/9
Piano	2016	1	1	1	1	1	1	1	1	1	9/9
Pico	2019	1	1	1	1	1	1	1	1	1	9/9
Pieri	2016	1	1	1	1	1	1	1	1	1	9/9
Pinholt	2003	1	1	1	1	1	1	1	1	1	9/9
Pistilli (1)	2013	1	1	1	1	1	1	1	1	1	9/9
Pistilli (2)	2013	1	1	1	1	1	1	1	1	1	9/9
Pjetursson	2009	1	1	0	1	1	1	1	1	1	8/9
Pohl	2017	1	1	0	1	1	1	1	1	1	8/9
Polizzi	2000	1	0	1	1	1	1	1	0	1	7/9
Pozzi (1)	2014	1	1	1	1	1	1	1	1	1	9/9
Pozzi (2)	2014	1	1	1	1	1	1	1	1	1	9/9
Priest	1999	1	1	1	1	1	1	1	0	1	8/9
Prosper	2010	1	1	0	1	1	1	1	1	1	8/9
Qian	2020	1	0	1	1	1	1	1	1	1	8/9
Queiroz	2015	1	1	0	1	1	1	1	1	1	8/9
Queridinha	2016	1	1	0	1	1	1	1	1	1	8/9
Rammelsberg	2014	1	1	0	1	1	1	1	1	0	7/9
Renouard	1999	1	1	1	1	1	1	1	0	1	8/9
Riben	2016	1	1	0	1	1	1	1	1	1	8/9
Rocci (1)	2003	1	1	1	1	1	1	1	1	1	9/9
Rocci (2)	2003	1	1	1	1	1	1	1	1	1	9/9
Roccuzzo (1)	2001	1	1	0	1	1	1	1	1	1	8/9
Roccuzzo (2)	2002	1	1	0	1	1	1	1	1	1	8/9
Romanos (1)	2000	1	1	0	1	1	1	1	0	1	7/9
Romanos (2)	2012	1	1	0	1	1	1	1	0	1	7/9
Romanos (3)	2014	1	1	0	1	1	1	1	0	1	7/9
Romeo (1)	2004	1	1	1	1	1	1	1	1	1	9/9
Romeo (2)	2009	1	1	1	1	1	1	1	1	1	9/9
Roos	1997	1	0	0	1	1	1	1	1	1	7/9
Rosenberg	2004	1	1	0	1	1	1	1	0	1	7/9
Rossi	2016	1	1	1	1	1	1	1	1	1	9/9
Salvi	2004	1	1	1	1	1	1	1	1	1	9/9
Santis	2016	1	1	0	1	1	1	1	1	1	8/9
Sbordone	2015	1	1	0	1	1	1	1	1	1	8/9
Schincaglia (1)	2007	1	1	1	1	1	1	1	1	1	9/9
Schincaglia (2)	2008	1	1	1	1	1	1	1	1	1	9/9
Schincaglia (3)	2015	1	0	0	1	1	1	1	1	1	7/9
Schnitman (1)	1997	1	1	0	1	1	1	1	0	1	7/9

Schnitman (2)	2011	1	0	1	1	1	1	1	0	1	7/9
Scurria	1998	1	1	1	1	1	1	1	1	1	9/9
Sener-Yamaner	2017	1	1	0	1	1	1	1	1	1	8/9
Sennerby	2012	1	1	1	1	1	1	1	1	1	9/9
Sethi	2000	1	0	0	1	1	1	1	1	1	7/9
Shi (1)	2017	1	1	0	1	1	1	1	1	0	8/9
Shi (2)	2019	1	1	0	1	1	1	1	1	1	8/9
Shigehara	2015	1	1	0	1	1	1	1	1	1	8/9
Si	2016	1	1	1	1	1	1	1	1	1	9/9
Smedberg	1991	1	1	1	1	1	1	1	0	1	8/9
Sohn	2010	1	0	0	1	1	1	1	1	1	7/9
Souza (1)	2018	1	1	0	1	1	1	1	1	1	8/9
Souza (2)	2019	1	1	0	1	1	1	1	0	1	7/9
Stanford	2016	1	1	0	1	1	1	1	1	0	8/9
Steveling	2001	1	1	1	1	1	1	1	0	1	8/9
Storelli	2018	1	1	0	1	1	1	1	1	1	8/9
Tallarico (1)	2017	1	1	1	1	1	1	1	1	1	9/9
Tallarico (2)	2018	1	1	1	1	1	1	1	1	0	9/9
Tang	2015	1	1	1	1	1	1	1	1	1	9/9
Taschieri (1)	2014	1	1	0	1	1	1	1	1	1	8/9
Taschieri (2)	2018	1	1	0	1	1	1	1	1	1	8/9
Tawil (1)	2001	1	1	0	1	1	1	1	0	1	7/9
Tawil (2)	2002	1	1	1	1	1	1	1	1	1	9/9
Tawil (3)	2003	1	1	1	1	1	1	1	1	1	9/9
Temmerman	2015	1	1	0	1	1	1	1	1	1	8/9
Testori (1)	2001	1	1	1	1	1	1	1	0	1	8/9
Testori (2)	2002	1	1	1	1	1	1	1	1	1	9/9
Testori (3)	2004	1	1	0	1	1	1	1	0	1	7/9
Testori (4)	2017	1	1	0	1	1	1	1	1	1	8/9
Thoma	2018	1	1	0	1	1	1	1	1	1	8/9
Thome	2020	1	1	1	1	1	1	1	1	1	9/9
Thone-Muhling	2020	1	1	0	1	1	1	1	1	0	8/9
Todisco	2018	1	1	1	1	1	1	1	1	1	9/9
Trbakovic	2018	1	1	0	1	1	1	1	1	1	8/9
Uraz	2020	1	1	0	1	1	1	1	1	1	8/9
Vandeweghe	2011	1	1	0	1	1	1	1	1	1	8/9
Vasak	2014	1	1	0	1	1	1	1	1	1	8/9
Veltri	2008	1	1	1	1	1	1	1	1	1	9/9
Vercruyssen	2010	1	1	1	1	1	1	1	1	1	9/9
Verdugo	2017	1	1	1	1	1	1	1	1	1	9/9
Vervaeke (1)	2012	1	1	1	1	1	1	1	1	1	9/9
Vervaeke (2)	2013	1	1	1	1	1	1	1	1	1	9/9
Vervaeke (3)	2015	1	1	1	1	1	1	1	1	1	9/9
Vigolo	2004	1	1	1	1	1	1	1	0	1	8/9
Villa	2005	1	1	1	1	1	1	1	0	1	8/9
Vogl	2015	1	1	0	1	1	1	1	1	1	8/9
Walton	2016	1	1	1	1	1	1	1	1	0	9/9
Wang (1)	2015	1	1	0	1	1	1	1	1	1	8/9
Wang (2)	2017	1	1	1	1	1	1	1	1	1	9/9
Weerapong	2019	1	1	0	1	1	1	1	1	1	8/9
Weng	2003	1	1	0	1	1	1	1	0	1	7/9
Wennerberg	1999	1	1	1	1	1	1	1	1	1	9/9
Widbom	2005	1	1	1	1	1	1	1	1	1	9/9
Winkler	2000	1	0	0	1	1	1	1	1	1	7/9
Wolfinger	2011	1	1	1	1	1	1	1	0	1	8/9
Wu (1)	2017	1	1	0	1	1	1	1	1	1	8/9
Wu (2)	2018	1	1	0	1	1	1	1	1	1	8/9
Wyatt	1998	1	1	1	1	1	1	1	0	1	8/9
Yamada	2015	1	1	1	1	1	1	1	1	1	9/9
Yang	2020	1	1	0	1	1	1	1	1	1	8/9

Yi	2013	1	1	0	1	1	1	1	1	1	8/9
Yildiz	2016	1	1	0	1	1	1	1	1	1	8/9
Yu	2017	1	1	1	1	1	1	1	1	1	9/9
Zadeh	2018	1	1	0	1	1	1	1	1	1	8/9
Zembic	2010	1	1	1	1	1	1	1	1	1	9/9
Zhang	2017	1	1	0	1	1	1	1	1	1	8/9
Zhou	2019	1	1	1	1	1	1	1	1	1	9/9
Zill	2016	1	0	1	1	1	1	1	1	1	8/9
Zumstein (1)	2012	1	1	1	1	1	1	1	1	1	9/9
Zumstein (2)	2016	1	1	1	1	1	1	1	1	1	9/9
Zweers	2015	1	1	1	1	1	1	1	1	1	9/9
van Steenberghe	1990	1	1	1	1	1	1	1	0	1	8/9
Örtorp	2008	1	1	1	1	1	1	1	1	1	9/9
Östman (1)	2008	1	1	1	1	1	1	1	0	1	8/9
Östman (2)	2012	1	0	1	1	1	1	1	0	1	7/9

Figure S1. Forest plot for the event 'implant failure'.

Studies	Estimate (95% C.I.)			Ev/Trt	Ev/Ctrl
Adanez 2018	1.000	(0.021,	48.086)	0/20	0/20
Al-Nawas 2015	2.106	(0.280,	15.856)	1/21	9/398
Alayan 2019	8.167	(0.178,	375.113)	0/5	0/48
Alsaadi (1) 2007	1.851	(1.272,	2.693)	29/456	223/6490
Alsaadi (2) 2008	1.679	(0.967,	2.915)	13/120	88/1364
Amato 2020	2.692	(0.111,	65.020)	1/77	0/69
Amorfini 2017	3.095	(0.139,	68.997)	0/6	1/64
Andersson 2015	2.725	(0.430,	17.291)	1/6	17/278
Anitua (1) 2008	1.217	(0.424,	3.497)	4/697	24/5090
Anitua (2) 2016	1.551	(0.146,	16.472)	2/49	1/38
Anitua (3) 2019	1.889	(0.041,	87.509)	0/8	0/16
Aparicio 1998	0.435	(0.058,	3.280)	1/21	8/73
Arlin 2006	0.553	(0.161,	1.900)	3/176	14/454
Arora 2018	15.000	(0.390,	576.690)	0/1	0/29
Arosio 2018	7.636	(0.533,	109.378)	0/2	5/251
Artzi 2010	7.718	(2.856,	20.857)	4/20	17/656
Arunjaroensuk 2018	30.000	(0.774,	1163.530)	0/1	0/59
Atieh 2014	0.360	(0.049,	2.668)	1/10	5/18
Babbush 2012	9.949	(0.874,	113.245)	0/1	24/974
Bae 2015	6.200	(0.352,	109.183)	0/3	2/123
Bahat (1) 1992	2.750	(0.363,	20.848)	1/6	4/66
Bahat (2) 1996	0.574	(0.055,	5.986)	1/27	2/31
Bahat (3) 2000	2.511	(1.346,	4.683)	13/103	28/557
Bain 1993	2.962	(2.005,	4.375)	28/185	102/1996
Balleri (1) 2002	2.917	(0.061,	139.094)	0/11	0/34
Balleri (2) 2010	2.818	(0.059,	133.646)	0/10	0/30
Balshi (1) 1997	1.434	(0.213,	9.637)	1/11	26/410
Balshi (2) 1999	1.392	(0.109,	17.758)	0/2	42/354
Balshi (3) 2005	19.846	(1.598,	246.432)	0/1	6/515
Balshi (4) 2017	5.800	(1.097,	30.663)	2/10	3/87
Bechara 2017	0.200	(0.010,	4.052)	0/45	2/45
Becker (1) 2000	0.498	(0.029,	8.478)	0/27	7/208
Becker (2) 2005	8.667	(0.438,	171.596)	0/2	1/77
Becker (3) 2013	1.750	(0.417,	7.346)	3/30	4/70
Becktor (1) 2002	1.845	(0.815,	4.178)	4/12	114/631
Becktor (2) 2004	1.726	(0.888,	3.355)	6/18	212/1098
Becktor (3) 2007	1.839	(0.128,	26.371)	0/4	20/376
Benlidayi 2018	2.011	(0.214,	18.875)	3/88	1/59
Bergendal 1998	3.533	(1.181,	10.574)	3/9	10/106
Bernard 2019	8.576	(0.173,	425.069)	0/32	0/282
Bernardi 2018	0.376	(0.140,	1.008)	5/86	13/84
Bianco 2000	9.195	(2.742,	30.830)	3/11	7/236
Bischof 2006	2.397	(0.407,	14.101)	3/102	2/163
Blomqvist 1998	1.968	(0.869,	4.453)	4/11	56/303
Blus 2006	75.667	(1.778,	3219.972)	0/2	0/226
Bogaerde (1) 2003	4.556	(0.310,	66.971)	0/2	4/122
Bogaerde (2) 2004	9.444	(0.477,	187.123)	0/2	1/84
Bogaerde (3) 2005	25.000	(0.646,	967.912)	0/1	0/49
Bolle 2018	0.666	(0.234,	1.896)	6/94	7/73
Boon 2020	1.394	(0.080,	24.232)	0/17	5/275
Borges 2020	13.000	(0.339,	498.450)	0/1	0/25
Bornstein 2008	0.337	(0.020,	5.649)	0/179	13/1638
Bouhy 2020	1.698	(0.687,	4.195)	8/43	8/73
Bressan 2014	0.159	(0.003,	7.697)	0/87	0/13
Browaeys 2013	6.563	(1.616,	26.655)	2/17	14/781
Brånemark 1995	1.335	(0.733,	2.429)	9/38	96/541
Brügger 2015	4.937	(1.627,	14.976)	5/256	8/2022
Buser 1997	1.679	(0.880,	3.205)	12/389	35/1905
Caban 2017	2.900	(0.320,	26.300)	1/5	2/29
Calandriello 2011	20.000	(3.434,	116.499)	1/1	1/39
Calvo-Guirado 2016	1.537	(0.065,	36.113)	1/40	0/20
Campos 2019	5.400	(0.325,	89.622)	0/2	2/80
Cannizzaro (1) 2012	0.552	(0.034,	9.011)	0/10	7/90
Cannizzaro (2) 2013	0.232	(0.028,	1.896)	1/38	5/44
Cannizzaro (3) 2016	9.200	(0.193,	438.967)	0/9	0/91
Cannizzaro (4) 2018	1.490	(0.429,	5.175)	6/152	4/151
Carr (1) 2003	0.244	(0.015,	3.977)	0/60	20/608
Carr (2) 2019	1.768	(1.053,	2.967)	14/190	266/6382
Cecchi 2010	0.739	(0.015,	35.417)	0/22	0/16
Cecchinato 2015	7.583	(0.363,	158.524)	0/3	1/90
Chiapasco 2014	12.857	(0.265,	623.858)	0/13	0/179
Clelland 2016	0.736	(0.032,	17.045)	1/52	0/12
Cochran (1) 2002	2.000	(0.241,	16.612)	2/6	1/6
Cochran (2) 2007	1.130	(0.127,	10.049)	1/179	4/809
Collaert (1) 2002	26.200	(0.576,	1191.053)	0/4	0/130
Collaert (2) 2011	44.667	(1.051,	1897.802)	0/2	0/133
Correia 2017	0.889	(0.471,	1.677)	13/231	29/458
Cosyn 2012	2.019	(0.648,	6.287)	3/44	38/1125
Cucchi 2019	2.690	(0.373,	19.381)	1/7	6/113
Dagorne 2015	2.800	(0.798,	9.819)	4/32	5/112
Dahlin 2013	2.146	(0.490,	9.393)	2/46	11/543
Davarpanah 2002	1.708	(0.957,	3.049)	15/285	40/1298
De Bruyn (1) 1992	5.083	(1.257,	20.563)	2/9	8/183
De Bruyn (2) 2002	2.107	(0.317,	14.018)	1/7	12/177
Degasperi 2014	1.087	(0.046,	25.783)	0/22	1/74
Degidi (1) 2005	0.780	(0.044,	13.861)	0/40	5/351
Degidi (2) 2006	0.397	(0.023,	6.769)	0/117	9/888
Degidi (3) 2009	3.312	(0.176,	62.162)	0/21	3/509

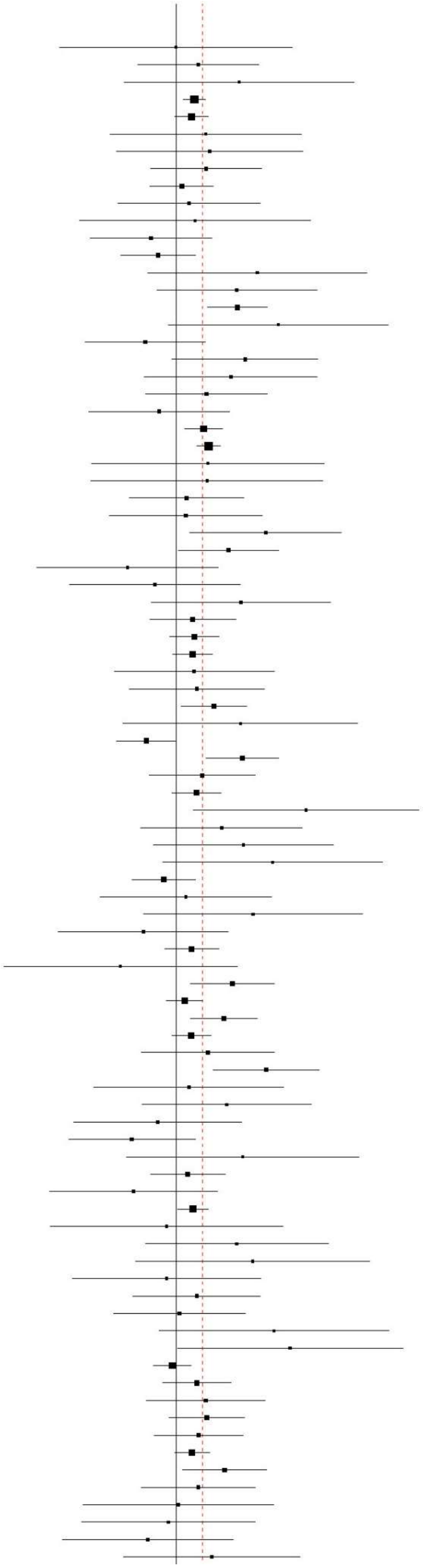

Degidi (4) 2012	0.383	(0.091, 1.609)	2/692	26/3443
Deporter (1) 2001	0.042	(0.002, 0.983)	0/135	1/16
Deporter (2) 2014	0.768	(0.391, 1.506)	16/99	12/57
Diaz−Sanchez 2017	1.556	(0.032, 76.084)	0/26	0/41
Ducommun 2019	2.188	(0.678, 7.068)	4/366	9/1802
Eckert 2001	0.993	(0.273, 3.616)	2/9	17/76
Eliasson 2009	1.553	(0.107, 22.647)	0/5	26/493
Engstrand 2003	1.000	(0.531, 1.882)	18/285	18/285
Esposito (1) 2015	1.912	(0.177, 20.710)	2/91	1/87
Esposito (2) 2016	0.694	(0.037, 13.135)	0/34	3/169
Esposito (3) 2017	3.000	(0.063, 143.124)	0/11	0/35
Esposito (4) 2019	0.667	(0.115, 3.865)	2/68	3/68
Evian 1996	6.433	(1.098, 37.709)	1/4	15/386
Felice (1) 2014	3.500	(0.334, 36.667)	1/18	2/126
Felice (2) 2015	0.667	(0.034, 12.956)	0/11	2/39
Felice (3) 2019	2.833	(0.571, 14.071)	5/60	2/68
Felice (4) 2019	1.517	(0.350, 6.573)	4/80	3/91
Feng 2014	0.769	(0.017, 35.513)	0/12	0/9
Fenlon 2002	0.577	(0.040, 8.419)	0/3	6/29
Fermergård 2012	3.818	(0.259, 56.312)	1/11	1/42
Ferrigno (1) 2002	1.955	(0.776, 4.930)	5/101	30/1185
Ferrigno (2) 2006	1.345	(0.452, 4.004)	4/103	14/485
Fischer 2008	1.704	(0.349, 8.316)	2/27	5/115
Fortin (1) 2002	13.371	(3.114, 57.424)	2/7	5/234
Fortin (2) 2017	5.852	(1.256, 27.275)	7/61	2/102
Friberg (1) 1992	12.769	(0.846, 192.628)	1/13	1/166
Friberg (2) 1997	1.878	(0.473, 7.455)	2/23	25/540
Friberg (3) 2002	1.720	(0.697, 4.242)	7/96	12/283
Friberg (4) 2003	1.431	(0.369, 5.553)	3/26	5/62
Friberg (5) 2005	10.614	(1.289, 87.384)	1/11	4/467
Friberg (6) 2008	5.212	(2.589, 10.491)	13/72	15/433
Fugazzotto 2002	0.611	(0.039, 9.524)	1/72	1/44
Gallucci 2004	75.000	(4.134, 1360.710)	2/4	0/74
Galluci 2009	10.950	(0.223, 537.237)	0/19	0/218
Garcez−Filho 2015	2.778	(0.143, 54.146)	2/26	0/14
Garlini 2003	1.666	(0.100, 27.811)	0/18	8/537
Gastaldi 2017	1.118	(0.023, 53.312)	0/16	0/18
Geckili 2014	1.886	(0.594, 5.986)	3/72	35/1584
Gholami 2014	3.167	(0.144, 69.461)	0/5	1/56
Glauser (1) 2001	1.404	(0.579, 3.401)	5/22	17/105
Glauser (2) 2003	2.333	(0.135, 40.186)	0/5	3/97
Glibert 2016	8.750	(0.182, 421.245)	0/11	0/104
Golab 2016	5.146	(1.664, 15.910)	7/114	5/419
Gopalakrishnan 2014	10.000	(0.262, 381.098)	0/1	0/19
Gotfredsen 1993	4.333	(0.221, 85.092)	0/2	1/38
Goto 2002	5.354	(1.622, 17.671)	2/5	13/174
Grunder 1999	15.877	(7.983, 31.576)	4/5	13/258
Guarnieri (1) 2014	0.760	(0.040, 14.599)	0/9	2/37
Guarnieri (2) 2016	5.750	(0.132, 250.282)	0/3	0/22
Guarnieri (3) 2019	1.213	(0.052, 28.470)	1/46	0/18
Guarnieri (4) 2019	1.100	(0.023, 52.888)	0/19	0/21
Guida 2020	1.000	(0.020, 49.750)	0/75	0/75
Gulje (1) 2013	2.832	(0.299, 26.781)	3/107	1/101
Gulje (2) 2019	2.864	(0.123, 66.443)	1/21	0/20
Haas 1996	3.005	(1.455, 6.203)	7/55	79/1865
Hallman (1) 2001	21.000	(0.882, 500.281)	1/22	0/160
Hallman (2) 2004	4.864	(0.810, 29.198)	1/4	11/214
Hallman (3) 2005	1.669	(0.421, 6.606)	2/13	13/141
Han 2014	0.790	(0.320, 1.950)	5/271	59/2525
Hatano 2011	2.911	(0.199, 42.514)	0/4	13/392
Hattingh 2019	1.042	(0.021, 51.454)	0/47	0/49
Hayacibara 2013	4.846	(0.101, 233.312)	0/12	0/62
He 2015	1.106	(0.592, 2.067)	14/763	31/1868
Hentenaar 2020	0.229	(0.005, 10.930)	0/47	0/10
Hentschel 2015	8.565	(0.444, 165.201)	3/312	0/382
Herreo−Climent 2014	2.773	(0.057, 135.549)	0/21	0/60
Herrman 2005	3.965	(2.093, 7.511)	12/55	23/418
Higuchi 1995	4.420	(1.372, 14.235)	6/120	5/442
Hoffmann 2006	21.333	(0.504, 902.628)	0/2	0/63
Hopp 2017	2.497	(0.819, 7.615)	3/48	88/3516
Horikawa 2014	3.579	(1.279, 10.018)	4/19	12/204
Hu 2020	1.093	(0.053, 22.397)	0/44	2/245
Hussaini 2010	3.031	(1.102, 8.341)	4/32	20/485
Imburgia 2015	1.083	(0.066, 17.864)	0/11	7/194
Ivanoff 1999	3.676	(1.594, 8.478)	19/127	7/172
Jang 2011	2.200	(1.084, 4.465)	8/224	100/6161
Jemt (1) 1991	5.000	(0.628, 39.792)	1/5	3/75
Jemt (2) 2006	2.893	(1.575, 5.314)	18/111	19/339
Jonker 2018	5.667	(0.287, 111.708)	0/2	1/50
Kahnberg 2008	76.500	(1.962, 2982.814)	0/1	0/152
Kaneda 2019	1.138	(0.075, 17.276)	0/6	13/214
Karlsson 1998	1.244	(0.062, 24.878)	0/17	2/111
Keller 1999	5.269	(2.218, 12.515)	2/3	31/245
Kennedy 2013	2.746	(0.167, 45.209)	7/70	0/12
Khayat 2001	1.517	(0.293, 7.858)	2/29	4/88
Khouly 2017	0.492	(0.070, 3.479)	1/20	20/197
Kim (1) 2015	36.500	(0.823, 1618.570)	0/3	0/145
Kim (2) 2018	3.294	(1.344, 8.071)	7/223	14/1469
Kinsel 2007	13.567	(4.930, 37.330)	11/48	5/296
Kokovic 2014	0.947	(0.019, 46.491)	0/37	0/35

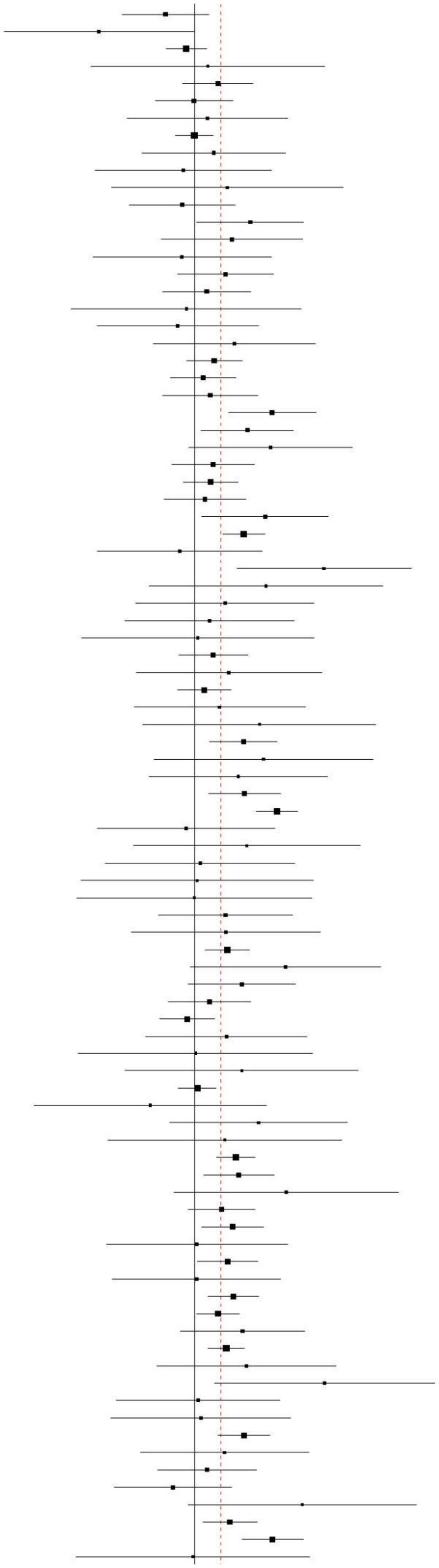

Koo 2010	0.105	(0.006, 1.741)	0/122	15/399
Kovacs 2000	7.404	(3.951, 13.876)	4/6	47/522
Lago 2018	6.269	(1.084, 36.249)	3/39	2/163
Lai (1) 2008	4.833	(0.220, 106.418)	0/5	1/86
Lai (2) 2008	0.900	(0.048, 16.992)	0/7	2/35
Lai (3) 2010	1.105	(0.365, 3.344)	6/133	6/147
Lee (1) 2013	1.376	(0.083, 22.827)	0/19	9/522
Lee (2) 2014	1.297	(0.026, 63.860)	0/36	0/47
Lee (3) 2018	1.041	(0.279, 3.881)	3/56	7/136
Lekholm 1999	0.764	(0.325, 1.794)	6/101	28/360
Levine (1) 1997	2.826	(0.057, 140.383)	0/45	0/129
Levine (2) 2007	2.697	(0.549, 13.234)	6/267	2/240
Lin 2018	1.075	(0.743, 1.554)	34/4938	160/24976
Lindgren 2012	6.000	(0.407, 88.368)	0/1	2/59
Lini 2019	1.458	(0.107, 19.935)	0/3	29/343
Lobato 2020	0.360	(0.015, 8.435)	0/24	1/26
Lopez−Cedrun 2011	8.429	(0.181, 392.467)	0/6	0/58
Lops 2013	4.750	(0.110, 205.793)	0/3	0/18
Ma 2013	0.411	(0.037, 4.525)	1/945	2/777
Makkonen 1997	5.458	(0.353, 84.316)	1/24	1/131
Malchiodi (1) 2011	4.667	(0.341, 63.780)	0/2	8/237
Malchiodi (2) 2017	1.581	(0.066, 37.907)	1/73	0/38
Malmstrom 2016	2.348	(0.099, 55.938)	1/45	0/35
Malo (1) 2015	4.908	(0.814, 29.584)	1/5	24/589
Malo (2) 2015	8.574	(1.858, 39.575)	2/18	6/463
Mangano (1) 2014	0.783	(0.106, 5.804)	1/99	18/1395
Mangano (2) 2014	8.944	(0.572, 139.960)	0/3	4/321
Manni 2020	2.600	(0.055, 122.364)	0/9	0/25
Mattsson 1999	1.211	(0.051, 28.535)	0/18	1/68
McGlumphy 2003	5.671	(2.053, 15.671)	4/20	14/397
Mei 2017	2.647	(0.055, 128.183)	0/16	0/44
Meloni 2018	7.400	(0.164, 333.133)	0/4	0/36
Mendonca (1) 2014	1.047	(0.178, 6.137)	3/129	2/90
Mendonca (2) 2017	2.078	(0.578, 7.472)	3/37	8/205
Mendonca (3) 2017	0.848	(0.113, 6.370)	1/11	6/56
Mericske−Stern 2002	1.794	(0.416, 7.732)	3/51	4/122
Merli 2008	5.000	(0.104, 239.860)	0/11	0/59
Mertens (1) 2011	9.750	(0.424, 224.034)	1/11	0/38
Mertens (2) 2012	0.429	(0.023, 8.002)	0/23	3/71
Mijiritsky 2013	2.372	(0.585, 9.629)	2/66	38/2975
Mo 2015	8.500	(0.194, 372.638)	0/3	0/33
Mongardini 2020	2.527	(0.539, 11.839)	2/51	7/451
Mozzati 2015	5.333	(0.380, 74.813)	0/2	6/207
Mumcu 2019	2.742	(0.056, 135.228)	0/30	0/84
Munakata 2016	5.500	(0.147, 205.115)	0/1	0/10
Nedir 2004	0.533	(0.028, 10.246)	0/111	3/417
Nevins 1993	1.012	(0.238, 4.309)	2/119	18/1084
Niedemaier 2017	1.011	(0.142, 7.201)	1/49	41/2031
Niimi 1998	4.083	(1.444, 11.551)	4/12	8/98
Nogueira 2018	4.625	(0.761, 28.115)	2/8	2/37
Norton 2017	6.750	(0.155, 294.773)	0/3	0/26
Olate 2010	3.074	(1.713, 5.517)	13/131	49/1518
Olson 2000	2.683	(0.716, 10.054)	2/9	14/169
Olsson (1) 1995	1.957	(0.492, 7.783)	2/23	24/540
Olsson (2) 2003	3.389	(0.262, 43.832)	0/1	4/60
Ormianer (1) 2006	2.893	(0.218, 38.448)	0/2	12/216
Ormianer (2) 2008	9.852	(0.668, 145.284)	0/2	4/265
Palmqvist 1994	3.220	(1.938, 5.348)	5/6	22/85
Payne 2017	0.423	(0.009, 20.654)	0/51	0/21
Penarrocha−Diago 2008	3.022	(0.587, 15.563)	2/23	4/139
Penarrocha−Oltra (1) 2012	3.371	(0.507, 22.419)	1/5	7/118
Penarrocha−Oltra (2) 2014	0.333	(0.032, 3.525)	1/48	2/32
Penarrocha−Oltra (3) 2015	1.248	(0.067, 23.256)	0/18	3/165
Petterson 2015	12.667	(0.564, 284.391)	0/6	1/265
Piano 2016	20.500	(0.464, 906.601)	0/3	0/81
Pico 2019	0.360	(0.007, 17.480)	0/49	0/17
Pieri 2016	0.411	(0.081, 2.074)	2/109	5/112
Pinholt 2003	1.500	(0.236, 9.519)	1/6	16/144
Pistilli (1) 2013	0.500	(0.046, 5.385)	1/68	2/68
Pistilli (2) 2013	0.162	(0.009, 3.094)	0/80	3/91
Pjetursson 2009	2.098	(0.238, 18.476)	4/164	1/86
Polizzi 2000	1.154	(0.131, 10.181)	1/13	3/45
Pozzi (1) 2014	52.636	(2.273, 1219.042)	1/10	0/192
Pozzi (2) 2014	27.167	(0.587, 1256.786)	0/5	0/162
Priest 1999	4.071	(0.250, 66.315)	0/3	3/113
Prosper 2010	0.843	(0.094, 7.552)	1/17	3/43
Qian 2020	1.810	(0.178, 18.392)	2/21	1/19
Queiroz 2015	11.408	(0.662, 196.658)	6/48	0/42
Queridinha 2016	4.185	(0.183, 95.520)	0/8	1/112
Rammelsberg 2014	2.140	(0.263, 17.433)	1/20	5/214
Renouard 1999	1.523	(0.325, 7.137)	6/65	2/33
Riben 2016	2.455	(0.172, 34.945)	0/2	5/80
Rocci (1) 2003	5.937	(1.277, 27.596)	1/2	8/95
Rocci (2) 2003	0.271	(0.017, 4.391)	0/16	11/105
Roccuzzo (1) 2001	6.778	(0.143, 320.729)	0/8	0/60
Roccuzzo (2) 2002	0.933	(0.041, 21.097)	0/9	1/27
Romanos (1) 2000	1.300	(0.068, 24.682)	0/7	2/51
Romanos (2) 2012	3.857	(0.237, 62.803)	0/3	3/107
Romanos (3) 2014	10.429	(0.224, 486.385)	0/6	0/72
Romeo (1) 2004	2.385	(1.008, 5.643)	6/72	24/687

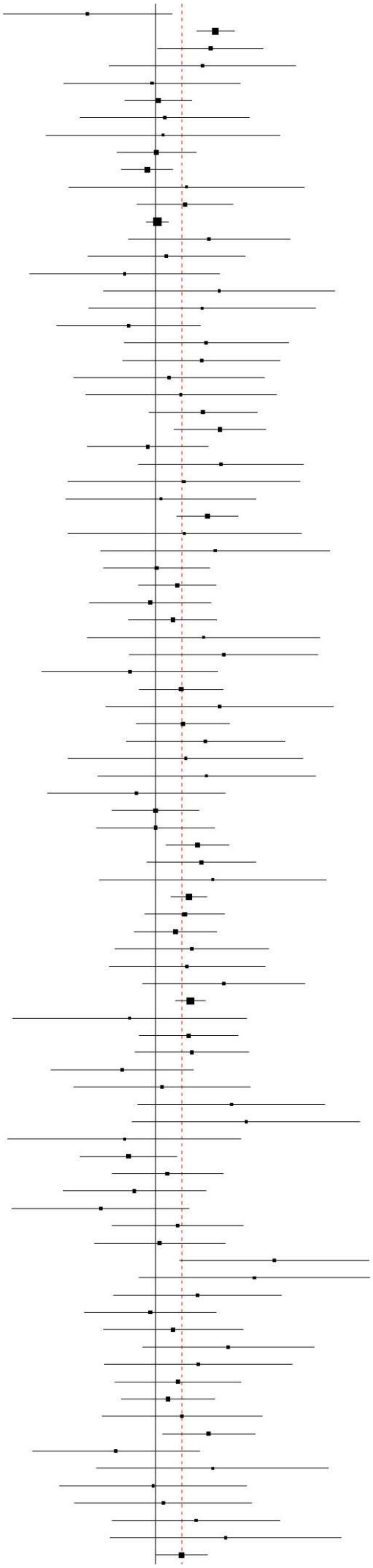

Romeo (2) 2009	7.429	(0.153,	359.728)	0/13	0/103
Roos 1997	12.304	(5.748,	26.340)	21/150	9/791
Rosenberg 2004	1.503	(0.948,	2.384)	19/163	104/1341
Rossi 2016	4.000	(0.474,	33.729)	4/30	1/30
Salvi 2004	16.250	(0.368,	717.486)	0/3	0/64
Santis 2016	1.422	(0.071,	28.452)	0/17	2/127
Sbordone 2015	18.833	(3.177,	111.642)	1/2	3/113
Schincaglia (1) 2007	2.733	(0.166,	44.991)	0/2	2/40
Schincaglia (2) 2008	0.852	(0.038,	19.032)	0/8	1/22
Schnitman (1) 1997	5.333	(0.859,	33.118)	2/9	2/48
Schnitman (2) 2011	0.356	(0.022,	5.690)	0/8	7/47
Scurria 1998	1.632	(0.617,	4.315)	4/29	30/355
Sener-Yamaner 2017	6.375	(0.130,	313.758)	0/23	0/152
Sennerby 2012	0.973	(0.090,	10.555)	1/74	2/144
Sethi 2000	4.470	(0.316,	63.254)	0/3	12/446
Shi (1) 2017	2.580	(0.476,	13.980)	4/269	2/347
Shi (2) 2019	3.622	(0.190,	69.200)	3/142	0/73
Shigehara 2015	10.333	(0.218,	490.355)	0/8	0/92
Si 2016	1.073	(0.307,	3.750)	4/41	5/55
Smedberg 1991	24.375	(5.607,	105.965)	5/8	2/78
Sohn 2010	0.253	(0.024,	2.710)	1/81	2/41
Souza (1) 2018	2.520	(0.108,	58.665)	1/24	0/20
Souza (2) 2019	11.000	(0.621,	194.957)	0/1	1/65
Stanford 2016	0.105	(0.006,	1.864)	0/63	5/73
Steveling 2001	4.111	(0.087,	193.512)	0/8	0/36
Storelli 2018	3.000	(0.129,	69.515)	1/20	0/20
Tallarico (1) 2017	4.200	(0.094,	187.119)	0/4	0/20
Tallarico (2) 2018	6.750	(0.467,	97.519)	1/8	1/54
Tang 2015	37.000	(0.799,	1713.094)	0/5	0/221
Taschieri (1) 2014	1.385	(0.028,	67.543)	0/25	0/35
Taschieri (2) 2018	1.372	(0.028,	67.797)	0/42	0/58
Tawil (1) 2001	5.382	(1.952,	14.844)	1/1	8/60
Tawil (2) 2002	1.172	(0.111,	12.427)	1/29	2/68
Tawil (3) 2003	2.308	(0.692,	7.698)	7/116	4/153
Temmerman 2015	8.273	(0.173,	396.486)	0/10	0/90
Testori (1) 2001	2.929	(0.353,	24.304)	1/31	5/454
Testori (2) 2002	0.967	(0.135,	6.917)	1/22	18/383
Testori (3) 2004	3.229	(0.192,	54.413)	0/4	3/112
Testori (4) 2017	4.800	(0.394,	58.449)	0/1	7/143
Thoma 2018	3.197	(0.133,	76.985)	1/60	0/64
Thome 2020	5.487	(0.275,	109.681)	0/15	2/438
Thone-Muhling 2020	1.450	(0.201,	10.460)	1/10	8/116
Todisco 2018	64.000	(1.643,	2493.756)	0/1	0/127
Trbakovic 2018	15.000	(0.390,	576.690)	0/1	0/29
Uraz 2020	1.880	(0.038,	91.933)	0/24	0/46
Vandeweghe 2011	1.202	(0.073,	19.683)	0/22	12/690
Vasak 2014	15.300	(1.031,	226.972)	1/10	1/153
Veltri 2008	9.714	(0.208,	452.843)	0/6	0/67
Vercruyssen 2010	0.441	(0.028,	6.990)	0/27	41/1024
Verdugo 2017	2.200	(0.047,	103.178)	0/9	0/21
Vervaeke (1) 2012	4.203	(1.691,	10.446)	9/192	9/807
Vervaeke (2) 2013	2.171	(0.090,	52.391)	0/40	1/266
Vervaeke (3) 2015	3.797	(1.630,	8.842)	10/255	11/1065
Vigolo 2004	0.876	(0.054,	14.085)	0/10	9/182
Villa 2005	48.500	(1.246,	1887.326)	0/1	0/96
Vogl 2015	27.500	(0.710,	1065.721)	0/1	0/54
Walton 2016	2.692	(0.147,	49.432)	0/12	3/244
Wang (1) 2015	7.000	(0.160,	305.897)	0/3	0/27
Wang (2) 2017	3.083	(0.063,	151.231)	0/23	0/73
Weerapong 2019	2.000	(0.195,	20.553)	2/23	1/23
Weng 2003	2.278	(1.405,	3.694)	20/97	43/475
Wennerberg 1999	2.878	(1.326,	6.245)	9/69	16/353
Widbom 2005	2.267	(0.199,	25.842)	0/1	21/194
Winkler 2000	25.786	(14.445,	46.031)	29/181	17/2736
Wolfinger 2011	7.545	(0.527,	108.072)	0/2	5/248
Wu (1) 2017	0.937	(0.481,	1.824)	10/298	51/1424
Wu (2) 2018	5.182	(0.411,	65.385)	0/1	5/113
Wyatt 1998	4.552	(1.445,	14.340)	3/13	11/217
Yamada 2015	7.667	(0.490,	119.922)	0/3	4/275
Yang 2020	1.115	(0.467,	2.661)	6/182	27/913
Yi 2013	3.333	(0.067,	165.456)	0/41	0/139
Yildiz 2016	3.300	(0.226,	48.130)	0/1	2/32
Yu 2017	0.359	(0.015,	8.553)	0/38	1/41
Zadeh 2018	3.741	(0.425,	32.908)	4/108	1/101
Zembic 2010	6.833	(0.841,	55.510)	1/3	2/41
Zhang 2017	0.706	(0.015,	34.351)	0/33	0/23
Zhou 2019	1.473	(1.029,	2.109)	40/1412	107/5565
Zill 2016	2.544	(0.163,	39.625)	0/5	7/228
Zumstein (1) 2012	7.543	(1.617,	35.196)	7/58	2/125
Zumstein (2) 2016	8.879	(0.434,	181.815)	2/57	0/102
Zweers 2015	39.333	(0.926,	1670.333)	0/2	0/117
van Steenberghe 1990	0.548	(0.165,	1.812)	3/120	20/438
Örtorp 2008	6.029	(2.058,	17.657)	16/140	4/211
Östman (1) 2008	6.788	(0.990,	46.559)	2/33	2/224
Östman (2) 2012	5.143	(0.230,	115.076)	0/6	1/107
**Overall (I^2=37.02 % , P< 0.001)**	2.437	(2.179,	2.725)	941/25490	4185/159435

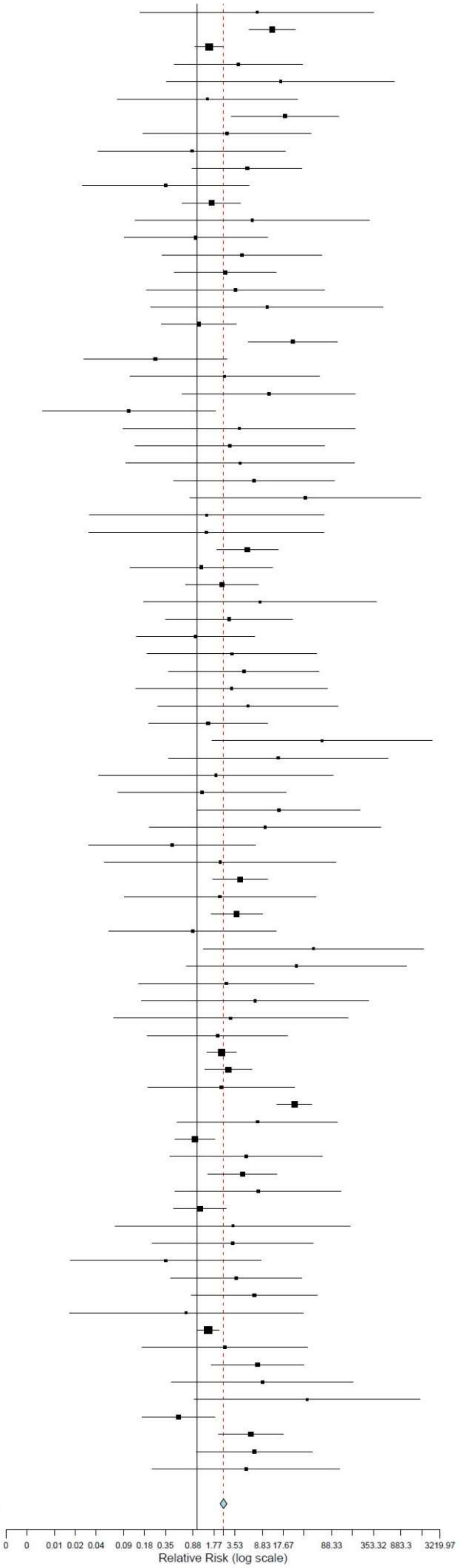

Supplement: Supplementary file 1 [file materials-14-03972-s001.zip › materials-1284199-supplementary.pdf]
